# Repressed hypoxia inducible factor‐1 in diabetes aggravates pulmonary aspergillus fumigatus infection through modulation of inflammatory responses

**DOI:** 10.1002/ctm2.273

**Published:** 2021-01-01

**Authors:** Yao Ye, Yu Chen, Jianjun Sun, Hanyin Zhang, Wenyang Li, Wei Wang, Xiaowei Zheng, Sergiu‐Bogdan Catrina

**Affiliations:** ^1^ Institute of Respiratory Disease The First Hospital of China Medical University Shenyang China; ^2^ Department of Respiratory Medicine Zhejiang Provincial People's Hospital People's Hospital of Hangzhou Medical College Hangzhou China; ^3^ Department of Pulmonary and Critical Care Medicine The Shengjing Hospital of China Medical University Shenyang China; ^4^ Department of Molecular Medicine and Surgery Karolinska Institutet Stockholm Sweden; ^5^ Center for Diabetes Academic Specialist Centrum Stockholm Sweden; ^6^ Department of Endocrinology and Diabetes Karolinska University Hospital Stockholm Sweden

Dear Editor,

Patients with diabetes have increased risk for infection, develop more severe infections, and have higher mortality compared with the general population.^1^ *Aspergillus fumigatus* (*A. fumigatus*) is the most common opportunistic aerial fungal pathogen that causes fatal invasive pulmonary aspergillosis (IPA) in immunocompromised patients.^2^ It has been suggested that diabetes is an independent risk factor for invasive aspergillosis in non‐immunocompromised patients.^3^ However, the underlying mechanism for the increased susceptibility of diabetic patients to *A. fumigatus* infection is still unclear.

The results from this study showed that diabetes was an independent risk factor for long‐term hospital stay of fungal pneumonia, indicating that the fungal pneumonia has a poor prognosis in patients with diabetes (Tables S1 and S2). We further investigated the influence of diabetes on pulmonary *A. fumigatus* infection using a streptozotocin‐induced mouse model of diabetes. We found a more severe course of the pulmonary *A. fumigatus* infection in diabetic mouse demonstrated by significantly reduced survival rate and clearance of *A. fumigatus* (Figures 1A‐1D), in addition to the reported increased fungal burden 24 hours post‐pulmonary *A. fumigatus* infection.^4^

**FIGURE 1:**
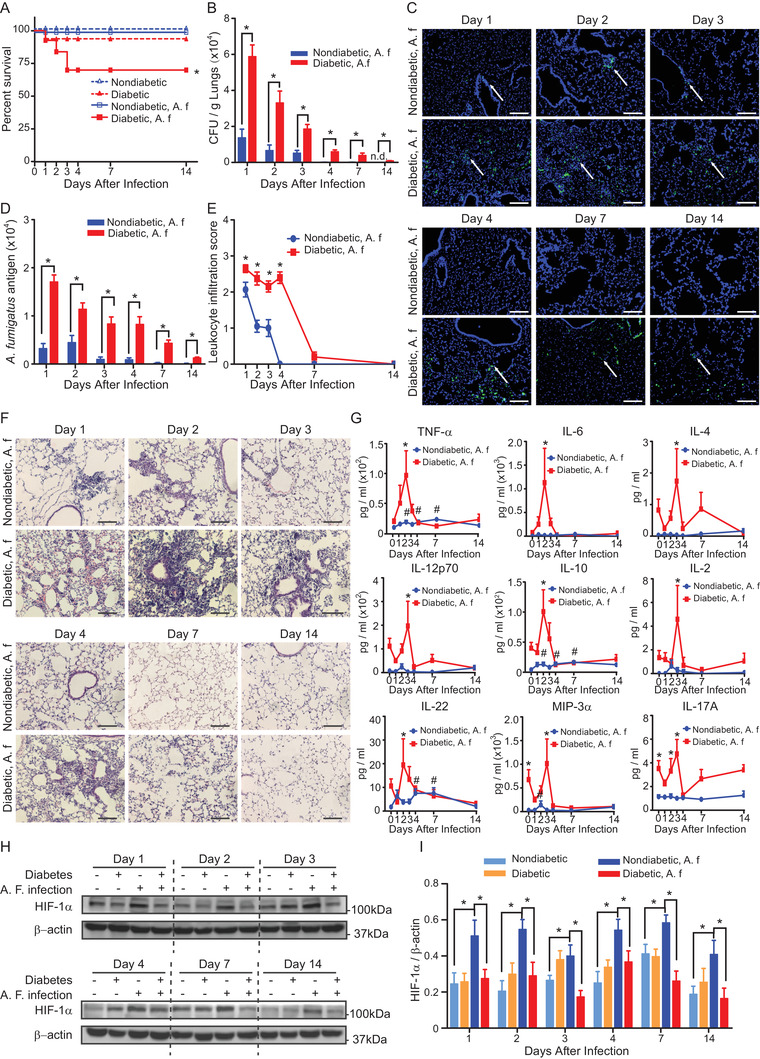
Diabetes reduces the survival rate and clearance of *A. fumigatus*, increases inflammatory responses and inhibits HIF‐1α induction in pulmonary *A. fumigatus* infection. Diabetic or nondiabetic mice were mock‐infected or received 5 × 10^8^ *A. fumigatus* conidia (A. f) intratracheally. A, Survival rate of the mice (n = 5, log‐rank test). B, Colony forming unit (CFU) counts per gram of lung tissue on indicated days post *A. fumigatus* challenge. N.d. denotes *A. fumigatus* was not detected (n = 5). C, Representative images of immunofluorescent staining of *A. fumigatus* (green) and DAPI (blue) in lung sections on indicated days post inoculation. Scale bars = 100µm. White arrows indicate positive staining of *A. fumigatus* conidia and hyphae. D, Quantification of the green fluorescence intensity of *A. fumigatus* (n = 5). B and D, *P < .05 analyzed using unpaired Student's *t*‐test. E, Quantification of leukocyte infiltration in panel F. F, Representative images of H&E staining of lung tissues harvested on indicated days post A. f inoculation. Scale bars = 100µm. G, Cytokine levels in serum on indicated days before (Day 0) and after *A. fumigatus* inoculation (n = 4 ‐ 5). E and G, *P < .05 compared between nondiabetic and diabetic groups analyzed using two‐way ANOVA followed by Bonferroni post‐hoc test. #, *P *< .05 compared with Day 0 in nondiabetic group using One‐way ANOVA followed by Fisher's LSD test. H, Representative images of HIF‐1α and β‐actin western blots. I, Quantification of HIF‐1α protein expression normalized to β‐actin (n = 5). *P < .05 analyzed for each day using RM One‐way ANOVA followed by Holm‐Sidak multiple comparisons test. Data are shown as mean ± SEM

The inflammatory and immune responses are both critical for the host defense against pulmonary *A. fumigatus* infection.^5^ A proper inflammatory response is fundamental for the clearance of the fungal infection. However, an overactive immune response with abrupt and massive release of cytokines referred to as hypercytokinemia or cytokine storm, can be more toxic than the invading pathogens themselves.^6^ In diabetic mice, pulmonary *A. fumigatus* infection induced overactivated inflammatory responses demonstrated by significantly increased and persistent leukocyte infiltration in the lung (Figures 1E and 1F) and remarkably elevated expression of plasma cytokines (Figure 1G). The abnormal response was most obvious in the early stage of infection. Transcriptome analysis of the lung tissue on the second day post‐infection revealed that the most enriched biological processes activated in diabetes were related with inflammatory and immune responses, such as cytokine‐cytokine receptor interaction, tumor necrosis factor (TNF) signaling pathway, nucleotide binding oligomerization domain‐like (NOD‐like) receptor, and Toll‐like receptor (TLR) signaling pathways (Figures 2J‐2L and 2N). Taken together, these results show that in diabetes there is a rapid overactive inflammatory response following pulmonary *A. fumigatus* infection which contributes to an increased lethality.

**FIGURE 2:**
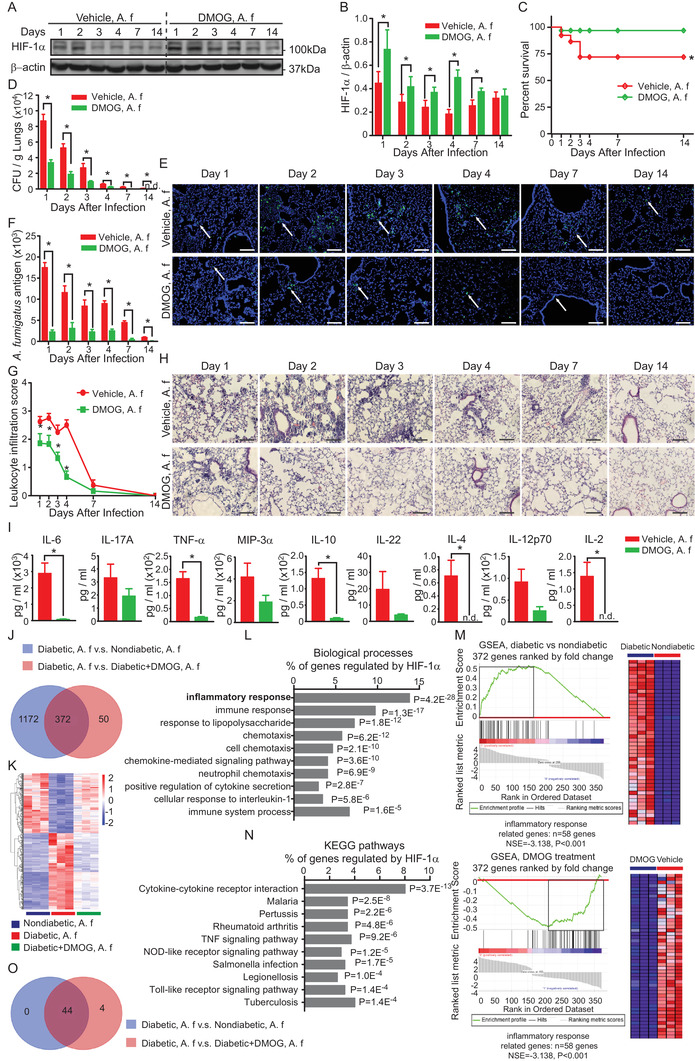
HIF‐1α induction attenuates the pulmonary infection and the inflammatory responses after pulmonary *A. fumigatus* infection in diabetic mice. Diabetic mice were injected with DMOG (300 mg/kg, *i.p*.) or vehicle every other day for 1 week before and 2 weeks after inoculation of 5 × 10^8^ *A. fumigatus* conidia *i.t*. (A. f). A, Representative images of HIF‐1α and β‐actin western blots. B, Quantification of HIF‐1α protein expression normalized to β‐actin (n = 5). *P < .05 analyzed for each day using paired Student's *t*‐test. C, Survival rate of vehicle‐ or DMOG‐treated diabetic mice with pulmonary *A. fumigatus* infection (log‐rank test). D, Colony forming unit (CFU) counts per gram of lung tissue on indicated days post *A. fumigatus* challenge. N.d. denotes *A. fumigatus* were not detected (n = 5). E, Representative images of immunofluorescent staining of *A. fumigatus* (green) and DAPI (blue) in lung sections on indicated days post‐inoculation. Scale bars = 100 µm. White arrows indicate positive staining of *A. fumigatus* conidia and hyphae. F, Quantification of the green fluorescence intensity of *A. fumigatus* (n = 5). D and F, *P < .05 analyzed using unpaired Student's *t*‐test. G, Quantification of leukocyte infiltration (n = 5). *P < .05 compared between nondiabetic and diabetic groups analyzed using two‐way ANOVA followed by Bonferroni post‐hoc test. H, Representative images of H&E staining of lungs harvested on indicated days post‐A. f inoculation. Scale bars = 100µm. I, Cytokine levels in serum 2 days after *A. fumigatus* inoculation (n = 3 ‐ 5). *P < .05 analyzed using unpaired Student's *t*‐test. B‐I, Data are shown as mean ± SEM. J‐O, RNA purified from lung tissues harvested 2 days after *A. fumigatus* inoculation was used for global transcriptome analysis (n = 3). J, Venn diagram showing the number of deferentially expressed genes (log2 fold change > 2 or < −2, *P *< .01) in different groups, among which 372 genes that were regulated by DMOG were also differentially expressed in diabetes after *A. fumigatus* infection. K, Heatmap diagram showing the relative expression of the 372 genes that were dysregulated by diabetes and reversed by DMOG treatment. L and N, The top 10 gene ontology biological processes and KEGG pathway in which the 372 genes are involved. *P*‐values were determined by Fisher's exact test. M, Gene set enrichment analysis (GSEA) showing the enrichment of the genes involved in the inflammatory responses that were affected by diabetes (upper panel) and by DMOG (lower panel). O, venn diagrams showing the leading‐edge subset genes in panel M Abbreviation: NES, normalized enrichment score.

Hypoxia is characteristically present in the lung of murine models of IPA.^7^ Hypoxia inducible factor‐1 (HIF‐1) is the key regulator of the cellular adaptive responses to hypoxia.^8^ Emerging evidence has shown that HIF‐1 plays an important role in regulating immunity and inflammation.^7^ HIF‐1 signaling is inhibited in diabetes secondary to the hyperglycemia‐induced HIF‐1α destabilization and functional repression.^9^ In this study, we found that in nondiabetic mice HIF‐1α expression was not only elevated immediately on day 1 after pulmonary *A. fumigatus* infection as shown previously, but also persisted until day 14 post‐infection.^4^ However, in diabetic mice, the induction of HIF‐1α was blunted at all the time points examined during the course of infection (Figures 1H and 1I) despite the even more hypoxic microenvironment in lung of diabetic mice after *A. fumigatus* inoculation (Figure S1).

To test the functional relevance of HIF‐1α repression for pulmonary *A. fumigatus* infection in diabetes, we investigated the effect of dimethyloxalylglycine (DMOG), a prolyl hydroxylase inhibitor known to induce HIF‐1α stabilization even in the presence of hyperglycemia.^10^ DMOG was given *i.p*. every other day starting from 1 week before *A. fumigatus* inoculation until day 14 post‐*A. fumigatus* inoculation. Indeed, pharmacological induction of HIF‐1α by DMOG (Figures 2A and 2B) was followed by significant improvement of pulmonary *A. fumigatus* infection (Figures 2C‐2F) and attenuated inflammatory responses (Figures 2G‐2I) in diabetic mice despite the same blood glucose levels (Figure S2), confirming a crucial role of HIF‐1 in host defense against *A. fumigatus* infection in diabetes. However, the application of DMOG after *A. fumigatus* inoculation lost the protective effect (Figure S3). Protective effect of alleviating pulmonary *A. fumigatus* infection in diabetes was also observed using specific PHD inhibitor FG‐4592 (Figure S4). Transcriptome analysis revealed that the HIF‐1 induction by DMOG reversed the dysregulated signaling pathways controlling inflammatory responses in diabetes, including cytokine‐cytokine receptor interaction, TNF signaling, NOD‐like receptor, and TLR signaling pathways (Figures 2K, 2M, and 2O; Figure S5). Thus, our results suggest that in diabetes a restoration of HIF‐1 function is not only essential for an effective immune response to control and clear *A. fumigatus*, but also crucial to dampen the overactive inflammatory responses which have fatal consequences.

In conclusion, our results have shown that the repression of HIF‐1 activation by diabetes during pulmonary *A. fumigatus* infection is persistent and contributes to an overactive inflammatory response with fatal consequences. Early and long‐term induction of HIF‐1 dampens the inflammation responses and protects diabetic mice against pulmonary *A. fumigatus* infection, indicating a promising future therapeutic strategy.

## CONFLICT OF INTEREST

The authors declare that there is no conflict of interest that could be perceived as prejudicing the impartiality of the research reported.

## ETHICS APPROVAL

All animal experiments were carried out in accordance with the National Institute of Health guide for the care and use of Laboratory animals, and experimental protocols were approved by the Institutional Animal Care and Use Committee of China Medical University. The clinical study was approved by the Ethical Review Board of the First Hospital of China Medical University.

## AUTHOR CONTRIBUTIONS

Study conception and design: Ye, Chen, Sun, Zhang, Li, Wang, Zheng, and Catrina. Laboratory experiments: Ye, Sun, Zhang, and Li. Analysis and interpretation of data: Ye, Wang, Zheng, and Catrina. Drafting of the manuscript: Ye, Wang, Zheng, and Catrina. Revision of the manuscript and final approval: Ye, Chen, Sun, Zhang, Li, Wang, Zheng, and Catrina

## Supporting information

Supporting Information

## Data Availability

Data presented in this manuscript are available upon request from the authors.
