# Supplementary material for: Repressed hypoxia inducible factor‐1 in diabetes aggravates pulmonary aspergillus fumigatus infection through modulation of inflammatory responses
Source: Clin Transl Med. 2021 Jan 1;11(1):e273. doi: 10.1002/ctm2.273 (PMC7775987; doi:10.1002/ctm2.273)
Supplement: Supplementary file 1 — Supporting Information [file CTM2-11-e273-s001.pdf]

Supplemental information for

Repressed Hypoxia inducible factor-1 in diabetes aggravates pulmonary aspergillus fumigatus infection through modulation of inflammatory responses

Yao Ye[1,2], Yu Chen[3], Jianjun Sun[1], Hanyin Zhang[1], Wenyang Li[1], Wei Wang[1#], Xiaowei Zheng[4]*, Sergiu-Bogdan Catrina[4,5,6]*

This document includes:

1. Detailed methods
2. Supplemental tables
3. Supplemental figures and figure legends
4. Gene lists

1. Detailed methods

Clinical retrospective analysis

Patients with fungal pneumonia, presenting at the First Hospital of China Medical University between March 2008 and July 2018, were identified by retrospectively reviewing medical records. The patients were categorized as with or without diabetes according to the 1999 WHO diabetes diagnostic criteria [1]. Patients with HIV infection, autoimmune hepatitis, autoimmune thyroiditis, idiopathic thrombocytopenia, systemic lupus erythematosus, drug-induced liver injury, or syphilis are enrolled in immune deficiency group. Patients were excluded if they had a malignant tumor. Age, sex, pathogen type, hospital stays, leukocyte counting and prognosis were extracted from each patient's medical records. This study was approved by the Ethical Review Board of the First Hospital of China Medical University

Fungal culture and growth conditions

Aspergillus fumigatus strain ATCC46645 was used in all experiments, and was grown on Sabouraud Dextrose Agar (SDA) at 37 ºC. Conidia were dislodged from plates with a cell scrapper, re-suspended in normal saline, and filtered through sterile gauze.

Animals

Male C57BL/6 mice (6 weeks old) were purchased from Liaoning Changsheng biotechnology co. LTD (Shenyang, China). Animals were housed in controlled temperature under 12/12h light/dark cycle, with standard chow and water *ad libitum*. All animal experiments were carried out in accordance with the National Institute of Health guide for the care and use of Laboratory animals, and experimental protocols were approved by the Institutional Animal Care and Use Committee of China Medical University.

Murine models of STZ-induced diabetes and pulmonary aspergillosis model

Diabetes was induced by a single dose intraperitoneal injection of streptozotocin (STZ, 180 mg / Kg, Sigma, USA) after a 12 hrs. fasting period [2,3]. In the subsequent days, blood was obtained by puncturing the tail vein, and blood glucose levels were checked with a glucometer (Accu-chek Performa, Roche, Switzerland). The diabetic state was confirmed after two sequential blood glucose measurements above 16.7 mmol / L one week after STZ injection. The mice were maintained in a diabetic state for one week prior to *A. fumigatus* inoculation. For infection, mice were lightly anesthetized and immobilized in an upright position using rubber bands attached to a cardboard for oropharyngeal aspiration. A blunt 24G needle attached to a 1ml syringe was advanced into the trachea to deliver 5 x 10^8 conidia in a volume of 0.05 ml saline.

DMOG treatment

Diabetic mice were randomized into two treatment groups: one group received dimethyloxalylglycine (DMOG) *i.p.* (300mg / Kg in PBS, Frontier Scientific, cat #: D1070, USA) every second day during the study period starting one week before infection. The diabetic control group was injected with the same volume of PBS at the same intervals.

FG-4592 treatment

Diabetic mice were randomized into two treatment groups: one group received FG-4592 *i.p.* (20mg / Kg, dissolved in DMSO and further diluted with PBS, Selleck Chemicals, cat #: S1007, USA) every second day during the study period starting one week before infection. The diabetic control group was injected with the same volume of vehicle at the same intervals.

Analysis of *A. fumigatus* infection in mice

To analyze *A. fumigatus* infection, the mice were sacrificed on day 1, 2, 3, 4, 7 and 14 post inoculation, and the left lung of each mouse was harvested and fixed in formaldehyde solution. The paraffin-embedded tissues were sliced with a rotary microtome (LEICA RM2245) for 4 μm thickness, then mounted on glass slides. After the procedures of dehydration, the sections were stained with hematoxylin and eosin (H & E) and observed using Zeiss Axioscope 2-plus microscope. Lung specimens were scored by two blinded investigators for the intensity of leukocyte infiltration. Semi-quantitative numeric values were assigned for these scores according to the scale: 0, no infiltrates; 1, little; 2, moderately large; and 3, large infiltrates [4].

To assess the fungal burden in lungs, the left lung sections were stained with a rabbit polyclonal antibody to Aspergillus (1:100, Abcam, cat #: ab20419, UK) at 4 °C overnight followed by washing 3 times with PBS for 5 min each. The sections were then incubated with AlexaFluor488-conjugated goat anti-rabbit IgG (1:100, Zsbio, cat #: ZF-0511, China) at room temperature for 1h. After washing 3 times with PBS for 5 minutes each, prolong Gold antifade reagent with DAPI (Solarbio, cat #: S2110, China) was added to each section. Microscopic examinations were performed on a Zeiss Axioscope 2-plus microscope and imaging system (Zeiss, Jena, Germany). Quantification of *A. fumigatus* was performed using Image-Pro Plus (U.S. Media Cybernetics, Silver spring, MD, USA) and integrated optical density (IOD) was measured.

In Colony Forming Unit (CFU) assays, the right upper lobe was harvested and homogenized with glass beads on a Mini- Bead beater (Service bio, KZ-II, China), and serially diluted onto SDA plates in duplicate, and CFU was determined after 24 hours.

Analysis of hypoxia level in mice

To analyze hypoxia level in lung tissue, Hypoxyprobe (60mg / Kg in PBS, Natural Pharmacia International, Inc., cat #: HP3-100Kit, USA) was injected intraperitoneally 2 hours before sacrifice. The mice were sacrificed on day 1 and day 3 post inoculation, and the left lung of each mouse was harvested and fixed in formaldehyde solution. The paraffin-embedded tissues were sliced with a rotary microtome (LEICA RM2245) for 4 μm thickness, then mounted on glass slides. After dewaxing and hydration, the left lung sections were blocked by animal non-immune serum and then stained with a rabbit anti-pimonidazole antibody (1:100, Natural Pharmacia International, Inc., cat #: PAb2627AP, USA) at 4 °C overnight followed by washing 3 times with PBS for 5 min each. The sections were then incubated with HRP-conjugated goat anti-rabbit IgG (1:100, MX biotechnologies, cat #: KIT-7710, China) at room temperature for 1h. After washing 3 times with PBS for 5 minutes each, DAB (Zsbio, cat #: ZLI-9018, China) was added to each section for 10 minutes. One minute after hematoxylin counterstaining, the sections were dehydrated, fixed and mounted. Microscopic examinations were performed on a Zeiss Axioscope 2-plus microscope and imaging system (Zeiss, Jena, Germany). Quantification of *A. fumigatus* was performed using Image-Pro Plus (U.S. Media Cybernetics, Silver spring, MD, USA) and integrated optical density (IOD) was measured.

Cytokine analysis

Selected cytokine levels in the serum were analyzed using Meso Scale Discovery (Gaithersburg, MD, USA) mouse Cytokine Assay (detecting IL-2, IL-4, IL-6, IL-10, IL-12p70, IL-17A, IL-22, MIP-3α and TNF-α) and measured using the QuickPlex SQ120 according to the manufacture's instruction.

Protein extraction and western blotting analysis

Lung tissues were homogenized in RIPA Lysis buffer at 4ºC to obtain whole tissue lysate. The lysates were then cleared by centrifugation for 30 min at 20,000 g at 4ºC and the protein concentration was determined using bicinchoninic acid method (ThermoFisher, cat #: 23227, USA). Fifty μg protein samples were separated by SDS-PAGE and transferred to PVDF membranes (Bio-Rad). After blocking in 5% non-fat milk in Tris-buffered saline (TBS) buffer [50 mM Tris (pH 7.4), 150 mM NaCl] supplemented with 0.1% Tween 20 (TBS-T) for 1.5 hours, the membranes were incubated with anti-HIF-1α (1:1000, Novus Bio, cat #: NB100-479, USA) or anti-β-actin (1:1000, Zsbio, cat #: TA-09, China) antibodies in blocking buffer. After several washes with TBS-T, the membranes were incubated with anti-rabbit or anti-mouse IgG-horseradish peroxidase conjugate (1:5000, Zsbio, cat #: ZB-2301, cat #: ZB-2305, China) in TBS buffer. After several washes, HIF-1α and β-actin were visualized using an enhanced chemiluminescence reagent (Bio-Rad, cat #: 1705062, France). Immunoreactive bands were photographed using a Gel-Pro-Analyzer (DNR Bio, MicroChemi 4.2, Israel) and quantified using Image J. Relative band density for HIF-1α was normalized to β-actin.

RNA sequencing and RNA-Seq data analysis

RNA from lung tissues of three mice in each group were stabilized with RNALater reagent (ThermoFisher Scientific) and extracted using RNeasy Mini Kit (Qiagen) according to the manufacturer's instructions. RNA integrity was assessed using the RNA Nano 6000 Assay Kit of the Bioanalyzer 2100 system (Agilent Technologies, CA, USA). Sequencing libraries were generated using NEBNext® UltraTM RNA Library Prep Kit for Illumina® (NEB, USA). The featureCounts v1.5.0-p3 was used to count the reads numbers mapped to each gene. The *P* values were adjusted using the Benjamin & Hochberg method. Corrected *P*-value of 0.01 and absolute log2 of fold change of 2 were set as the threshold for significantly differential expression.

Statistical analysis

All data were expressed as the mean ± standard error of mean and analyzed with GraphPad Prism 5.0 software (San Diego, CA, USA). Chi-square test, Student's t-test and Fisher's exact test were used to analyze the clinical data. And multivariate logistic regression was used to further confirm the risk factors. Multiple-group comparisons were analyzed using one-way or two-way ANOVA followed by multiple comparisons post-test testing. Unpaired Student's t-test was used for the comparison between two groups. Survival curves were analyzed with Mantel-Cox log-rank tests. $P < 0.05$ was considered statistically significant.

1. Gabir MM, Hanson RL, Dabelea D, et al. The 1997 American Diabetes Association and 1999 World Health Organization criteria for hyperglycemia in the diagnosis and prediction of diabetes. *Diabetes Care.* 2000; 23(8): 1108-12.
2. De Carvalho AK, Da Silva S, Serafini E, et al. Prior Exercise Training Prevent Hyperglycemia in STZ Mice by Increasing Hepatic Glycogen and Mitochondrial Function on Skeletal Muscle. *J Cell Biochem.* 2017; 118(4): 678-85.
3. Erener S, Mojibian M, Fox JK, Denroche HC, Kieffer TJ. Circulating miR-375 as a biomarker of beta-cell death and diabetes in mice. *Endocrinology.* 2013; 154(2): 603-8.
4. Mirkov I, El-Muzghi AA, Djokic J, et al. Pulmonary immune responses to Aspergillus fumigatus in rats. *Biomed Environ Sci.* 2014; 27(9): 684-94.

2. Supplemental tables

Table S1. Univariate analysis of risk factors for the length of hospital stay.

Factor	Short (n = 47)	Long (n = 43)	P value
Age (Mean ± SEM years)	51.62±2.089	54.28±2.008	0.294*
Diabetes status			0.039†
Yes	14 (38.9)	22 (61.1)	
No	33 (61.1)	21 (38.9)	
Thoracic surgery history			0.252‡
Yes	2 (28.6)	5 (71.4)	
No	45 (54.2)	38 (45.8)	
Immune deficiency			0.022†
Yes	12 (36.4)	21 (63.6)	
No	35 (61.4)	22 (38.6)	
Kidney disease			1.000‡
Yes	3 (50)	3 (50)	
No	44 (52.4)	40 (47.6)	

< 20 days of stay in hospital was defined as a short-term stay and ≥ 20 days of stay was defined as a long-term stay. The data are presented as number (%).

* Student's t test, † Chi-square test, ‡ Fisher's exact test

Table S2. The multivariate logistic regression analysis.

Variable	B value	OR (95% Confidence Interval)	P value
Diabetes status	1.038	2.823 (1.136-7.014)	0.025
Immune deficiency	1.149	3.156 (1.2748-7.985)	0.015

3. Supplemental figures and figure legends

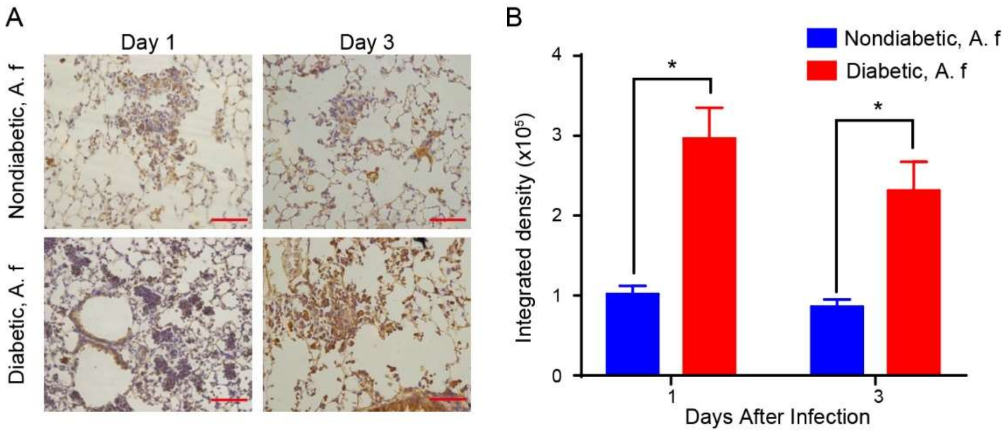

Figure S1. Lung tissues in diabetic mice are more hypoxic than nondiabetic mice after pulmonary *A. fumigatus* infection. Diabetic or nondiabetic mice were received 5 x 10^8 *A. fumigatus* conidia (A. f) intratracheally. Hypoxyprobe was injected intraperitoneally 2 hours before sacrifice. A, Representative images of immunohistochemical staining of Hypoxyprobe in lung sections on indicated days post inoculation. Scale bars = 100µm. B, Quantification of the immunohistochemical staining intensity of Hypoxyprobe (n = 5). Data are presented as mean ± SEM. *, $P < 0.05$ analyzed using Two-way ANOVA followed by multiple comparisons test.

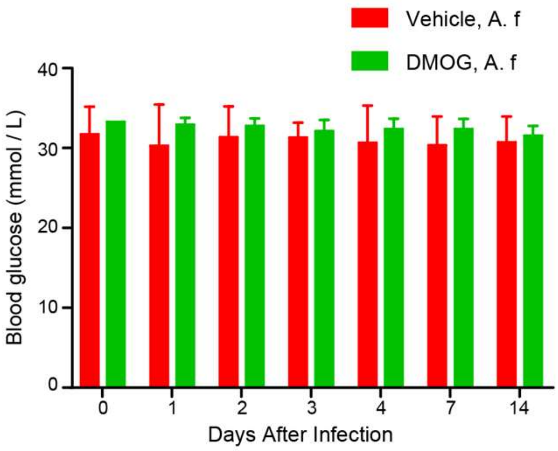

Figure S2. Diabetic mice were administered DMOG (300 mg/Kg, *i.p.*) or vehicle every other day one week before and two weeks after *A. fumigatus* infection (A. f). Blood glucose was measured on the indicated days before (Day 0) and after the inoculation (n=5).

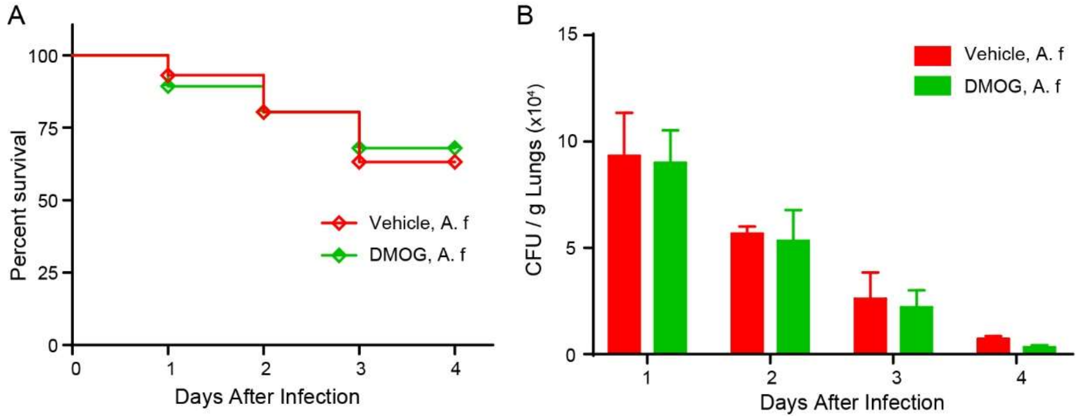

Figure S3. Diabetic mice were injected with DMOG (300 mg / Kg, *i.p.*) or vehicle every other day for 4 days after inoculation of 5 x 10^8 *A. fumigatus* conidia *i.t.* (A. f). A, Survival rate of the mice (n = 5, Log-Rank Test). B, Colony forming unit (CFU) counts per gram of lung tissue on indicated days post *A. fumigatus* challenge (n = 3 - 5).

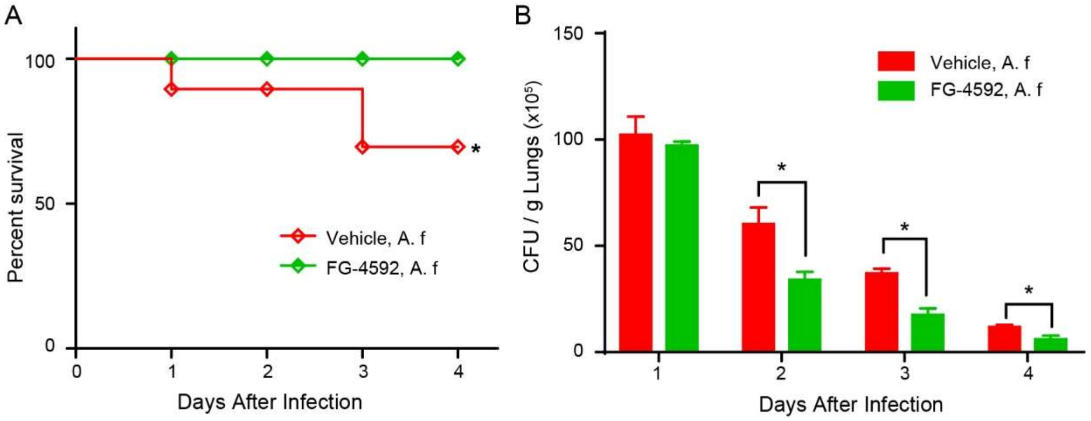

Figure S4. Diabetic mice were injected with FG-4592 (20 mg / Kg, *i.p.*) or vehicle every other day for one week before and 4 days after inoculation of 5 x 10^8 *A. fumigatus* conidia *i.t.* (A. f). A, Survival rate of the mice (n = 5, Log-Rank Test). B, Colony forming unit (CFU) counts per gram of lung tissue on indicated days post *A. fumigatus* challenge (n = 3 - 5). *, $P < 0.05$ analyzed using unpaired Student's t-test.

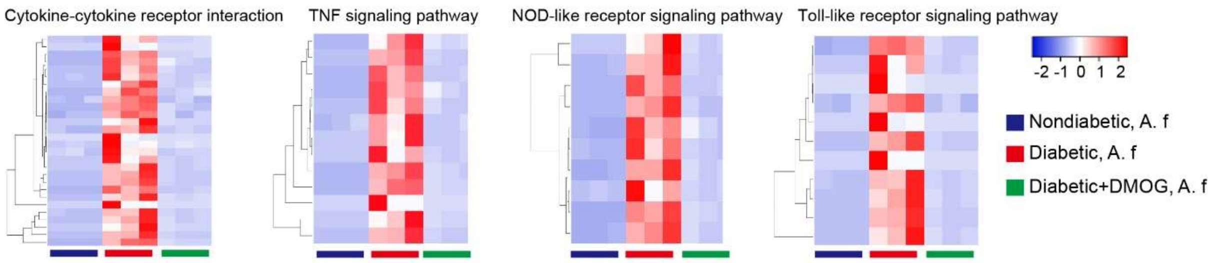

Figure S5. Heatmap diagrams showing the relative expression of genes in inflammation-related pathways analyzed by KEGG pathway analysis in Figure 2N.

List 1. Differentially Expressed Genes between Diabetic, A. f vs Nondiabetic A. f

gene_id	gene_name	padj (diabetic vs nondiabetic)	Fold-Change (diabetic vs nondiabetic)
ENSMUSG00000000031	H19	0.009985938	0.166051202
ENSMUSG00000000182	Fgf23	0.004779587	12.47971149
ENSMUSG00000000204	Slfn4	1.30E-11	43.93092057
ENSMUSG00000000290	Itgb2	4.06E-73	8.464829477
ENSMUSG00000000325	Arvcf	7.92E-27	0.220752352
ENSMUSG00000000385	Tmprss2	1.05E-16	0.240911563
ENSMUSG00000000416	Cttnbp2	8.31E-06	0.228819355
ENSMUSG00000000562	Adora3	1.49E-08	7.342768511
ENSMUSG00000000682	Cd52	1.10E-36	5.755010684
ENSMUSG00000000730	Dnmt3l	1.98E-06	7.504836014
ENSMUSG00000000791	Il12rb1	1.59E-05	5.901630852
ENSMUSG00000000903	Vpreb3	1.92E-30	0.11639807
ENSMUSG00000000957	Mmp14	4.16E-43	6.205516112
ENSMUSG00000000982	Ccl3	4.29E-31	265.3431412
ENSMUSG00000000983	Wfdc18	3.26E-05	98.1432878
ENSMUSG00000001020	S100a4	2.45E-05	5.583769409
ENSMUSG00000001095	Slc13a2	0.000731503	4.30924016
ENSMUSG00000001131	Timp1	7.10E-191	81.05246061
ENSMUSG00000001156	Mxd1	1.21E-62	10.66813084
ENSMUSG00000001473	Tubb6	2.86E-28	6.307117614
ENSMUSG00000001750	Tcirg1	9.09E-42	4.909169914
ENSMUSG00000001865	Cpa3	1.38E-05	0.194535534
ENSMUSG00000002068	Ccne1	4.42E-06	4.650078463
ENSMUSG00000002111	Spi1	1.41E-40	6.269785944
ENSMUSG00000002500	Rpl3l	0.003958367	4.258420833
ENSMUSG00000002588	Pon1	2.06E-68	0.060837085
ENSMUSG00000002699	Lcp2	9.22E-61	9.603575349
ENSMUSG00000002769	Gnmt	1.36E-10	0.228651683
ENSMUSG00000002847	Pla1a	7.36E-26	12.12357924
ENSMUSG00000002897	Il17ra	7.70E-23	4.738156183
ENSMUSG00000002983	Relb	1.17E-23	4.438367271
ENSMUSG00000003206	Ebi3	1.48E-17	7.508933932
ENSMUSG00000003283	Hck	4.00E-37	4.294020581
ENSMUSG00000003477	Inmt	3.55E-25	0.101976957
ENSMUSG00000003484	Cyp4f18	1.83E-35	12.42602525
ENSMUSG00000003665	Has1	2.91E-07	5.62813515
ENSMUSG00000003882	Il7r	6.05E-21	4.407130743
ENSMUSG00000004035	Gstm7	8.48E-06	0.249433326
ENSMUSG00000004207	Psap	9.86E-27	4.719770322
ENSMUSG00000004266	Ptpn6	1.07E-34	4.156881642
ENSMUSG00000004359	Spic	1.56E-06	10.7066703
ENSMUSG00000004360	9330159F19Rik	0.000116018	0.17731175
ENSMUSG00000004371	Il11	6.16E-05	5.439592966
ENSMUSG00000004446	Bid	1.70E-25	4.519732574
ENSMUSG00000004609	Cd33	9.45E-28	9.872349374
ENSMUSG00000004709	Cd244a	1.73E-17	4.011156956
ENSMUSG00000004791	Pgf	2.51E-10	4.252290435
ENSMUSG00000004814	Ccl24	4.61E-09	7.520270475
ENSMUSG00000004892	Bcan	4.29E-07	0.011564909
ENSMUSG00000005054	Cstb	3.13E-41	12.00865313
ENSMUSG00000005057	Sh2b2	1.11E-44	27.24464566
ENSMUSG00000005087	Cd44	6.64E-30	5.30505969
ENSMUSG00000005320	Fgfr4	8.73E-11	0.154631943
ENSMUSG00000005373	Mlxipl	0.000313744	0.170763541
ENSMUSG00000005413	Hmox1	1.72E-37	27.80655712
ENSMUSG00000005540	Fcer2a	1.64E-10	4.76454107
ENSMUSG00000005547	Cyp2a5	2.18E-08	0.067534238
ENSMUSG00000005667	Mthfd2	9.84E-15	5.00731423
ENSMUSG00000005800	Mmp8	4.20E-08	35.91504923
ENSMUSG00000005824	Tnfsf14	1.92E-17	5.56618945
ENSMUSG00000006014	Prg4	0.00222033	19.04858442
ENSMUSG00000006056	Calcoco2	0.000982471	0.08538581
ENSMUSG00000006269	Atp6v1b1	3.13E-14	0.028232776

List 1 continued

ENSMUSG00000006369	Fbln1	0.005678012	0.238458711
ENSMUSG00000006403	Adamts4	3.38E-69	65.687058
ENSMUSG00000006462	A530013C23Rik	0.000524231	4.219720376
ENSMUSG00000006519	Cyba	8.46E-24	5.163292224
ENSMUSG00000006567	Atp7b	3.30E-10	0.246868054
ENSMUSG00000006711	D130043K22Rik	0.000195496	0.20162569
ENSMUSG00000006724	Cyp27b1	0.000816855	18.41560312
ENSMUSG00000006784	Ttc25	1.63E-09	0.232658721
ENSMUSG00000006818	Sod2	3.51E-45	6.767083094
ENSMUSG00000007279	Scube2	1.92E-30	0.119835292
ENSMUSG00000007682	Dio2	0.000412997	5.633610651
ENSMUSG00000007946	Phox2a	1.34E-08	117.5994875
ENSMUSG00000009185	Ccl8	9.22E-15	9.146331154
ENSMUSG00000009214	Mymk	9.90E-05	21.64461411
ENSMUSG00000009292	Trpm2	1.64E-12	7.720871175
ENSMUSG00000009900	Wnt3a	3.73E-13	0.211859404
ENSMUSG00000010362	Rdm1	1.62E-21	0.221062308
ENSMUSG00000010651	Acaa1b	6.47E-16	0.084690655
ENSMUSG00000011008	Mcoln2	1.87E-27	9.748604827
ENSMUSG00000011148	Adssl1	2.72E-11	5.299598173
ENSMUSG00000011154	Cfap161	1.03E-08	0.218652689
ENSMUSG00000012123	Crybg2	2.41E-10	0.22626291
ENSMUSG00000012428	Steap4	8.00E-101	13.27824274
ENSMUSG00000012519	Mlkl	4.40E-10	6.320771184
ENSMUSG00000012819	Cdh23	7.00E-07	4.725085388
ENSMUSG00000013353	4931406B18Rik	0.000283408	75.35631904
ENSMUSG00000013974	Mcemp1	8.66E-20	5.276533117
ENSMUSG00000014543	Klra17	8.04E-08	6.423532032
ENSMUSG00000014599	Csf1	7.83E-25	4.43570653
ENSMUSG00000014686	Ceacam16	1.02E-07	17.29267118
ENSMUSG00000014782	Plekhg4	8.67E-05	8.257345956
ENSMUSG00000014846	Tppp3	3.09E-29	0.198043419
ENSMUSG00000015312	Gadd45b	1.37E-23	4.964339948
ENSMUSG00000015340	Cybb	4.28E-51	8.788551854
ENSMUSG00000015354	Pcolce2	3.01E-32	0.089416776
ENSMUSG00000015437	Gzmb	1.44E-07	4.016502225
ENSMUSG00000015854	Cd5l	0.000121159	7.099058027
ENSMUSG00000015947	Fcgr1	2.92E-18	11.01258122
ENSMUSG00000015950	Ncf1	2.32E-41	5.966288257
ENSMUSG00000016256	Ctsz	1.45E-37	5.39162304
ENSMUSG00000016283	H2-M2	4.28E-08	5.070271416
ENSMUSG00000016349	Eef1a2	1.35E-06	0.119978207
ENSMUSG00000016386	Mpped2	3.44E-08	0.188975857
ENSMUSG00000016496	Cd274	5.61E-39	22.42449759
ENSMUSG00000016498	Pdcd1lg2	6.35E-29	18.76584784
ENSMUSG00000016529	Il10	3.91E-08	129.1821376
ENSMUSG00000017002	Slpi	1.12E-29	19.92518516
ENSMUSG00000017344	Vtn	3.91E-07	0.245586803
ENSMUSG00000017491	Rarb	7.51E-08	0.227911966
ENSMUSG00000017631	Abr	2.44E-31	4.512234305
ENSMUSG00000017724	Etv4	0.000225987	5.098649584
ENSMUSG00000017737	Mmp9	7.05E-16	5.762675061
ENSMUSG00000017754	Pltp	5.18E-26	0.178304028
ENSMUSG00000017830	Dhx58	0.003877223	4.779023423
ENSMUSG00000017868	Sgk2	0.006199747	29.77022893
ENSMUSG00000018411	Mapt	3.60E-24	0.170278542
ENSMUSG00000018451	6330403K07Rik	7.52E-05	0.209660629
ENSMUSG00000018566	Slc2a4	0.001453651	0.24170566
ENSMUSG00000018581	Dnah11	3.04E-07	0.23979687
ENSMUSG00000018774	Cd68	2.41E-38	10.30849311
ENSMUSG00000018776	Slc35g3	0.007034365	0.160870532
ENSMUSG00000018868	Pnpla5	0.000519359	0.017163999
ENSMUSG00000018893	Mb	3.28E-06	0.015254189
ENSMUSG00000018919	Tm4sf5	0.00641202	4.032851041

List 1 continued

ENSMUSG00000018930	Ccl4	5.00E-96	230.4394182
ENSMUSG00000019102	Aldh3a1	6.97E-14	0.174625088
ENSMUSG00000019122	Ccl9	3.58E-31	16.43637671
ENSMUSG00000019278	Dpep1	3.06E-14	0.22528013
ENSMUSG00000019368	Sec14l4	6.38E-22	0.24029631
ENSMUSG00000019762	Iyd	7.28E-06	0.179380683
ENSMUSG00000019832	Rab32	1.13E-42	9.735635149
ENSMUSG00000019850	Tnfaip3	1.05E-63	15.09667011
ENSMUSG00000019874	Fabp7	1.33E-06	84.56179976
ENSMUSG00000019894	Slc6a15	5.48E-08	0.150318285
ENSMUSG00000019905	Gprc6a	4.08E-05	0.242027467
ENSMUSG00000019987	Arg1	1.22E-79	83.63839739
ENSMUSG00000019992	Mtfr2	5.25E-05	7.255974152
ENSMUSG00000020052	Ascl1	0.00037487	0.048672778
ENSMUSG00000020062	Slc5a8	0.000228152	0.089717756
ENSMUSG00000020077	Srgn	1.13E-16	6.216188148
ENSMUSG00000020096	Tbata	8.81E-07	0.076197917
ENSMUSG00000020097	Sgpl1	3.40E-28	4.681421059
ENSMUSG00000020120	Plek	1.29E-51	12.49153863
ENSMUSG00000020178	Adora2a	1.43E-27	5.248777831
ENSMUSG00000020182	Ddc	2.07E-07	0.173299449
ENSMUSG00000020218	Wif1	5.98E-09	0.11821602
ENSMUSG00000020227	Irak3	5.06E-29	10.07713264
ENSMUSG00000020251	Glt8d2	3.12E-13	0.106555363
ENSMUSG00000020256	Aldh1l2	0.000298675	4.053976345
ENSMUSG00000020334	Slc22a4	8.65E-23	24.49985099
ENSMUSG00000020377	Ltc4s	3.27E-06	0.243480882
ENSMUSG00000020399	Havcr2	7.21E-25	6.510698191
ENSMUSG00000020400	Tnip1	2.67E-35	7.063104332
ENSMUSG00000020407	Upp1	8.26E-27	8.082492153
ENSMUSG00000020431	Adcy1	5.61E-10	0.04346502
ENSMUSG00000020524	Gria1	4.46E-09	0.169055063
ENSMUSG00000020572	Nampt	5.28E-14	4.559727737
ENSMUSG00000020620	Abca8b	1.77E-13	0.192922147
ENSMUSG00000020641	Rsad2	1.21E-06	32.12861469
ENSMUSG00000020644	Id2	3.10E-12	4.424399797
ENSMUSG00000020651	Slc26a4	4.54E-25	11.88032379
ENSMUSG00000020682	Mmp28	5.18E-29	0.185220715
ENSMUSG00000020684	Rasl10b	2.04E-19	9.742132293
ENSMUSG00000020826	Nos2	6.25E-68	61.91897814
ENSMUSG00000020836	Coro6	0.000247216	0.221148453
ENSMUSG00000020884	Asgr1	1.31E-09	0.062823191
ENSMUSG00000020901	Pik3r5	9.82E-30	4.902511961
ENSMUSG00000020990	Cdkl1	1.75E-05	0.204908478
ENSMUSG00000021025	Nfkbia	2.22E-14	4.394033255
ENSMUSG00000021055	Esr2	6.34E-07	0.179905474
ENSMUSG00000021091	Serpina3n	1.87E-71	11.30976395
ENSMUSG00000021109	Hif1a	7.51E-31	5.387660216
ENSMUSG00000021123	Rdh12	0.000277941	12.04586106
ENSMUSG00000021125	Arg2	6.08E-27	13.57852688
ENSMUSG00000021186	Fbln5	2.21E-16	0.246541182
ENSMUSG00000021198	Unc79	2.16E-06	0.130085172
ENSMUSG00000021278	Amn	0.003383318	15.4400543
ENSMUSG00000021281	Tnfaip2	1.05E-79	13.25759319
ENSMUSG00000021298	Gpr132	4.83E-39	6.614284702
ENSMUSG00000021322	Aoah	6.94E-48	26.02086521
ENSMUSG00000021379	Id4	5.77E-11	0.230437199
ENSMUSG00000021390	Ogn	9.90E-19	0.178510813
ENSMUSG00000021403	Serpinb9b	1.20E-07	5.633035629
ENSMUSG00000021451	Sema4d	7.61E-55	12.43225978
ENSMUSG00000021456	Fbp2	1.03E-05	10.54660916
ENSMUSG00000021457	Syk	3.23E-28	6.099266392
ENSMUSG00000021567	Nkd2	4.58E-19	0.183467959
ENSMUSG00000021573	Tppp	3.90E-14	0.157302375

List 1 continued

ENSMUSG00000021591	Glrx	3.15E-23	7.042394205
ENSMUSG00000021614	Vcan	7.01E-35	14.31597978
ENSMUSG00000021640	Naip1	7.62E-07	11.18106126
ENSMUSG00000021684	Pde8b	3.12E-22	0.196812251
ENSMUSG00000021708	Rasgrf2	5.46E-11	0.210608445
ENSMUSG00000021765	Fst	5.49E-07	4.420027254
ENSMUSG00000021768	Dusp13	5.89E-07	17.17613848
ENSMUSG00000021779	Thrb	7.26E-13	0.219141495
ENSMUSG00000021798	Ldb3	2.51E-06	0.20152815
ENSMUSG00000021822	Plau	4.36E-17	6.399387682
ENSMUSG00000021922	Itih4	3.22E-22	4.191031663
ENSMUSG00000021939	Ctsb	7.46E-35	6.269225564
ENSMUSG00000021943	Gdf10	6.80E-06	0.235121374
ENSMUSG00000021986	Amer2	3.64E-17	17.02812239
ENSMUSG00000021990	Spata13	4.06E-21	4.699888904
ENSMUSG00000021996	Esd	3.01E-27	7.69994683
ENSMUSG00000021997	Lrrc63	0.00885911	6.664250969
ENSMUSG00000021998	Lcp1	2.27E-26	4.971436962
ENSMUSG00000022026	Olfm4	1.33E-05	28.67562233
ENSMUSG00000022094	Slc39a14	4.25E-67	8.737885142
ENSMUSG00000022096	Hr	1.28E-13	0.178678123
ENSMUSG00000022097	Sftpc	0.000238613	0.14993943
ENSMUSG00000022122	Ednrb	1.21E-12	0.199381878
ENSMUSG00000022126	Acod1	1.21E-229	1697.405047
ENSMUSG00000022148	Fyb	3.43E-39	6.377785072
ENSMUSG00000022180	Slc7a8	2.51E-56	16.91083479
ENSMUSG00000022197	Pdzd2	0.001250752	0.22745852
ENSMUSG00000022206	Npr3	9.04E-19	0.160647997
ENSMUSG00000022218	Tgm1	5.25E-38	9.596664943
ENSMUSG00000022262	Dnah5	5.10E-09	0.198980719
ENSMUSG00000022303	Dcstamp	1.71E-22	84.59882475
ENSMUSG00000022304	Dpys	6.37E-05	20.33597147
ENSMUSG00000022346	Myc	1.75E-21	4.934405914
ENSMUSG00000022367	Has2	6.60E-06	13.89308569
ENSMUSG00000022372	Sla	2.80E-14	4.06170538
ENSMUSG00000022378	Fam49b	9.90E-39	4.256372356
ENSMUSG00000022435	Upk3a	0.000997674	0.018807457
ENSMUSG00000022439	Parvg	3.95E-27	4.433283914
ENSMUSG00000022449	Adamts20	0.002756593	0.147516768
ENSMUSG00000022456	3-Sep	2.38E-06	0.235556487
ENSMUSG00000022490	Ppp1r1a	2.65E-05	0.149122674
ENSMUSG00000022500	Litaf	9.64E-34	6.913589608
ENSMUSG00000022510	Trp63	0.000124548	5.251542766
ENSMUSG00000022534	Mefv	3.64E-37	33.73451435
ENSMUSG00000022546	Gpt	1.83E-16	0.198949348
ENSMUSG00000022564	Grina	1.23E-30	6.188517586
ENSMUSG00000022584	Ly6c2	2.28E-10	5.175110863
ENSMUSG00000022586	Ly6i	1.83E-99	123.3372728
ENSMUSG00000022595	Lypd2	7.45E-21	0.12725135
ENSMUSG00000022650	Retnlb	7.82E-06	115.8356293
ENSMUSG00000022651	Retnlg	3.85E-06	10.98205374
ENSMUSG00000022652	Morc1	0.000403325	8.594593668
ENSMUSG00000022667	Cd200r1	2.47E-13	4.672337653
ENSMUSG00000022758	P2rx6	6.02E-14	0.136562152
ENSMUSG00000022759	Lrrc74b	1.80E-09	0.18044745
ENSMUSG00000022831	Hcls1	2.94E-63	7.889017196
ENSMUSG00000022840	Adcy5	8.66E-19	0.149762053
ENSMUSG00000022876	Samsn1	7.03E-33	13.70023981
ENSMUSG00000022878	Adipoq	0.004001095	0.057502207
ENSMUSG00000022901	Cd86	4.62E-18	6.364671275
ENSMUSG00000022902	Stfa2	2.21E-11	181.2698628
ENSMUSG00000022941	Ripply3	1.60E-14	0.236740694
ENSMUSG00000022945	Chaf1b	2.87E-13	4.03219643
ENSMUSG00000022996	Wnt10b	1.35E-11	0.188608492

List 1 continued

ENSMUSG00000023011	Faim2	1.11E-19	0.102925282
ENSMUSG00000023045	Soat2	3.83E-24	19.01310387
ENSMUSG00000023078	Cxcl13	6.59E-29	20.78170732
ENSMUSG00000023087	Noct	5.50E-14	4.052278922
ENSMUSG00000023088	Abcc1	5.58E-14	4.322433944
ENSMUSG00000023092	Fhl1	1.90E-20	0.236828913
ENSMUSG00000023336	Wfdc1	3.53E-14	0.195544869
ENSMUSG00000023349	Clec4n	4.50E-56	33.75549839
ENSMUSG00000023473	Celsr3	6.77E-09	8.408367058
ENSMUSG00000023903	Mmp25	4.61E-31	10.21846974
ENSMUSG00000023913	Pla2g7	1.07E-68	29.46701967
ENSMUSG00000023947	Nfkbie	8.89E-58	9.690491722
ENSMUSG00000023949	Tcte1	6.95E-11	0.174179788
ENSMUSG00000023982	Guca1a	1.35E-06	4.561598304
ENSMUSG00000023987	Pgc	5.16E-05	0.011342063
ENSMUSG00000023992	Trem2	8.41E-16	7.884165137
ENSMUSG00000024008	Cpne5	1.88E-12	0.088383877
ENSMUSG00000024014	Pim1	2.30E-53	7.623795082
ENSMUSG00000024027	Glp1r	7.80E-18	0.158873784
ENSMUSG00000024039	Cbs	5.55E-07	0.21378467
ENSMUSG00000024041	Cryaa	0.001272025	0.161369426
ENSMUSG00000024053	Emilin2	9.62E-28	11.97016648
ENSMUSG00000024121	Atp6v0c	2.13E-25	4.154903012
ENSMUSG00000024164	C3	2.21E-12	5.370715296
ENSMUSG00000024186	Rgs11	8.56E-09	0.246013462
ENSMUSG00000024222	Fkbp5	0.00374751	4.013473477
ENSMUSG00000024300	Myo1f	9.56E-51	6.268892749
ENSMUSG00000024349	Tmem173	3.73E-19	4.653167349
ENSMUSG00000024354	Slc23a1	7.71E-10	0.244428147
ENSMUSG00000024397	Aif1	6.77E-13	7.181528518
ENSMUSG00000024401	Tnf	7.43E-128	368.3201252
ENSMUSG00000024402	Lta	0.000249935	5.345436445
ENSMUSG00000024440	Pcdh12	1.46E-09	0.231158427
ENSMUSG00000024521	Pmaip1	1.26E-26	4.38758448
ENSMUSG00000024526	Cidea	0.002302039	0.175726625
ENSMUSG00000024598	Fbn2	0.000377865	0.027567237
ENSMUSG00000024621	Csf1r	3.86E-22	4.104890467
ENSMUSG00000024661	Fth1	9.84E-31	8.553760479
ENSMUSG00000024672	Ms4a7	1.24E-17	6.793315909
ENSMUSG00000024675	Ms4a4c	0.000451937	7.952393056
ENSMUSG00000024679	Ms4a6d	3.93E-43	18.99537541
ENSMUSG00000024737	Slc15a3	5.98E-80	12.92728355
ENSMUSG00000024770	Lipn	0.000580809	68.16930324
ENSMUSG00000024774	Ankrd22	1.10E-06	14.57313389
ENSMUSG00000024798	Htr7	7.88E-26	20.21591029
ENSMUSG00000024803	Ankrd1	8.93E-22	7.623173152
ENSMUSG00000024907	Gal	9.67E-07	0.145773888
ENSMUSG00000024953	Prdx5	5.63E-63	8.541310834
ENSMUSG00000025017	Pik3ap1	3.75E-28	6.365639157
ENSMUSG00000025044	Msr1	1.51E-115	65.15012469
ENSMUSG00000025150	Cbr2	3.01E-24	0.14358325
ENSMUSG00000025161	Slc16a3	9.18E-35	11.86029763
ENSMUSG00000025165	Sectm1a	1.67E-06	20.86993407
ENSMUSG00000025175	Fn3k	3.01E-05	0.089038637
ENSMUSG00000025225	Nfkb2	2.07E-27	4.689157256
ENSMUSG00000025318	Jph3	3.26E-06	13.7285589
ENSMUSG00000025321	Itgb8	9.10E-09	5.575199538
ENSMUSG00000025355	Mmp19	5.92E-16	4.419847403
ENSMUSG00000025383	Il23a	1.19E-16	50.78814098
ENSMUSG00000025429	Pstpip2	1.18E-46	9.404831365
ENSMUSG00000025473	Adam8	1.56E-09	14.90574738
ENSMUSG00000025479	Cyp2e1	7.82E-05	0.056338338
ENSMUSG00000025488	Cox8b	2.22E-06	0.135908506
ENSMUSG00000025491	Ifitm1	1.47E-15	4.499221909

List 1 continued

ENSMUSG00000025498	Irf7	0.00110774	9.473183021
ENSMUSG00000025576	Rbfox3	4.58E-05	0.234603551
ENSMUSG00000025591	Tma16	2.49E-29	5.026239014
ENSMUSG00000025658	Cnksr2	3.91E-08	0.135405812
ENSMUSG00000025746	Il6	4.75E-13	128.5313558
ENSMUSG00000025784	Clec3b	0.000967242	0.181919566
ENSMUSG00000025789	St8sia2	3.41E-09	0.171713603
ENSMUSG00000025804	Ccr1	4.27E-62	27.03619308
ENSMUSG00000025854	Fam20c	3.01E-27	9.143303908
ENSMUSG00000025877	Hk3	5.24E-99	37.003006
ENSMUSG00000025900	Rp1	2.91E-08	0.204752878
ENSMUSG00000025929	Il17a	0.000465094	317.4293269
ENSMUSG00000025936	Gm4956	9.24E-13	0.136203951
ENSMUSG00000025963	Mdh1b	3.82E-05	0.244686156
ENSMUSG00000025993	Slc40a1	3.10E-10	4.096770841
ENSMUSG00000026011	Ctla4	1.15E-22	11.161765
ENSMUSG00000026068	Il18rap	6.97E-18	4.837901591
ENSMUSG00000026073	Il1r2	1.27E-69	95.9813252
ENSMUSG00000026109	Tmeff2	1.01E-07	0.216791464
ENSMUSG00000026166	Ccl20	6.62E-10	22.12129923
ENSMUSG00000026177	Slc11a1	3.33E-82	36.07569496
ENSMUSG00000026180	Cxcr2	1.44E-27	10.67373329
ENSMUSG00000026193	Fn1	7.29E-19	4.412839219
ENSMUSG00000026205	Slc23a3	0.002582428	4.158356912
ENSMUSG00000026237	Nmur1	0.003690939	0.117917801
ENSMUSG00000026271	Gpr35	1.97E-35	9.876376658
ENSMUSG00000026285	Pdcd1	9.55E-11	9.793475505
ENSMUSG00000026301	Iqca	2.27E-10	0.20447359
ENSMUSG00000026358	Rgs1	1.55E-31	21.41536675
ENSMUSG00000026390	Marco	1.83E-15	4.229018091
ENSMUSG00000026475	Rgs16	2.87E-21	10.98402608
ENSMUSG00000026480	Ncf2	6.16E-31	5.163463858
ENSMUSG00000026536	Ifi211	1.67E-07	17.85386194
ENSMUSG00000026573	Xcl1	0.008695896	4.492572033
ENSMUSG00000026580	Selp	2.28E-35	11.34401679
ENSMUSG00000026582	Sele	3.91E-16	9.750339672
ENSMUSG00000026610	Esrrg	0.000323972	0.245428212
ENSMUSG00000026628	Atf3	1.80E-16	5.971392583
ENSMUSG00000026656	Fcgr2b	6.97E-48	14.66243702
ENSMUSG00000026676	Ccdc3	0.000798567	0.056221337
ENSMUSG00000026691	Fmo3	3.20E-18	0.080384481
ENSMUSG00000026749	Nek6	4.16E-20	4.130806977
ENSMUSG00000026768	Itga8	5.19E-25	0.166650067
ENSMUSG00000026786	Apbb1ip	1.70E-25	4.578851487
ENSMUSG00000026821	Ralgds	2.21E-33	4.671816651
ENSMUSG00000026822	Lcn2	1.24E-156	42.60425184
ENSMUSG00000026824	Kcnj3	0.007235808	0.075156276
ENSMUSG00000026874	Hc	0.004477609	0.179050409
ENSMUSG00000026879	Gsn	8.11E-19	0.199772893
ENSMUSG00000026961	Lrrc26	1.44E-10	0.158995673
ENSMUSG00000026979	Psd4	5.58E-25	4.096753661
ENSMUSG00000026981	Il1rn	6.18E-180	100.7363338
ENSMUSG00000026984	Il1f6	2.17E-05	135.1112892
ENSMUSG00000027068	Dhrs9	5.42E-11	5.484093841
ENSMUSG00000027199	Gatm	4.31E-09	4.970944294
ENSMUSG00000027219	Slc28a2	4.92E-10	4.064449874
ENSMUSG00000027220	Syt13	0.001709915	8.858590217
ENSMUSG00000027296	Itpka	1.04E-09	0.206321266
ENSMUSG00000027333	Smox	2.65E-21	5.377467572
ENSMUSG00000027368	Dusp2	1.45E-15	4.157260392
ENSMUSG00000027376	Prom2	1.58E-05	0.09632493
ENSMUSG00000027398	Il1b	7.90E-59	46.71511426
ENSMUSG00000027399	Il1a	1.06E-100	95.66406742
ENSMUSG00000027442	Cst8	6.85E-18	0.023804885

List 1 continued

ENSMUSG00000027483	Bpifa1	0.000150683	0.08063213
ENSMUSG00000027500	Stmn2	2.70E-23	0.188094412
ENSMUSG00000027513	Pck1	1.86E-05	0.052631554
ENSMUSG00000027514	Zbp1	7.62E-06	14.29554446
ENSMUSG00000027524	Edn3	3.38E-11	0.235721196
ENSMUSG00000027555	Car13	2.47E-42	7.457076288
ENSMUSG00000027559	Car3	9.46E-23	0.037233943
ENSMUSG00000027560	Dok5	6.17E-05	25.63412077
ENSMUSG00000027579	Srms	4.50E-06	0.234206565
ENSMUSG00000027611	Procr	1.68E-44	13.332059
ENSMUSG00000027670	Ocstamp	2.51E-13	30.27615486
ENSMUSG00000027716	Trpc3	6.86E-07	0.15228372
ENSMUSG00000027737	Slc7a11	3.30E-134	267.9960415
ENSMUSG00000027776	Il12a	7.67E-13	6.606409415
ENSMUSG00000027796	Smad9	2.51E-14	0.151379367
ENSMUSG00000027832	Ptx3	4.69E-07	38.25712855
ENSMUSG00000027875	Hmgcs2	1.88E-09	0.140459252
ENSMUSG00000027995	Tlr2	2.28E-84	22.05509583
ENSMUSG00000028001	Fga	1.79E-07	12.28964414
ENSMUSG00000028003	Lrat	4.41E-12	0.176142093
ENSMUSG00000028011	Tdo2	0.000328644	5.335459776
ENSMUSG00000028036	Ptgfr	5.68E-12	0.142026821
ENSMUSG00000028064	Sema4a	2.27E-26	4.076859806
ENSMUSG00000028088	Fmo5	1.08E-23	0.212869207
ENSMUSG00000028111	Ctsk	5.22E-18	4.885831581
ENSMUSG00000028116	Myoz2	1.38E-06	0.102405616
ENSMUSG00000028128	F3	1.14E-20	6.801163043
ENSMUSG00000028139	Riiad1	2.04E-08	0.240326462
ENSMUSG00000028255	Clca1	5.31E-14	0.008803941
ENSMUSG00000028270	Gbp2	0.000106504	12.11334918
ENSMUSG00000028327	Stra6l	8.61E-11	8.06372984
ENSMUSG00000028332	Hemgn	0.008940549	0.053069318
ENSMUSG00000028356	Ambp	1.11E-05	21.64944276
ENSMUSG00000028359	Orm3	2.03E-08	7.187613117
ENSMUSG00000028364	Tnc	6.46E-85	14.4160296
ENSMUSG00000028417	Tal2	0.000332876	13.54935099
ENSMUSG00000028434	Epb41l4b	3.90E-24	0.192960812
ENSMUSG00000028441	1110017D15Rik	2.63E-09	0.210965158
ENSMUSG00000028488	Sh3gl2	4.30E-11	0.089501607
ENSMUSG00000028599	Tnfrsf1b	2.43E-80	16.38198225
ENSMUSG00000028602	Tnfrsf8	1.20E-27	22.61613691
ENSMUSG00000028613	Lrp8	1.49E-52	20.26394632
ENSMUSG00000028645	Slc2a1	3.18E-29	5.848087253
ENSMUSG00000028655	Mfsd2a	1.54E-05	0.244131091
ENSMUSG00000028680	Plk3	3.00E-20	7.347745691
ENSMUSG00000028730	Cfap57	6.24E-14	0.158895753
ENSMUSG00000028766	Alpl	5.81E-36	11.41121997
ENSMUSG00000028780	Sema3c	2.38E-19	0.243501819
ENSMUSG00000028793	Rnf19b	1.63E-38	5.359074565
ENSMUSG00000028845	Tekt2	1.80E-08	0.218623899
ENSMUSG00000028859	Csf3r	7.83E-18	9.369264802
ENSMUSG00000028874	Fgr	2.60E-51	9.024889639
ENSMUSG00000028885	Smpdl3b	4.31E-40	14.84563983
ENSMUSG00000028940	HeS1	1.37E-14	0.01987271
ENSMUSG00000028965	Tnfrsf9	1.87E-92	38.01319221
ENSMUSG00000028970	Abcb1b	8.75E-14	4.805698985
ENSMUSG00000028996	Rbp7	0.000353119	0.116509519
ENSMUSG00000029075	Tnfrsf4	3.28E-08	5.696448952
ENSMUSG00000029082	Bst1	1.08E-63	10.95796274
ENSMUSG00000029088	Kcnip4	0.001136397	0.234728066
ENSMUSG00000029120	Ppp2r2c	8.92E-09	0.118919036
ENSMUSG00000029168	Dpysl5	5.49E-09	0.207951022
ENSMUSG00000029182	1700001C02Rik	6.92E-08	0.22555372
ENSMUSG00000029273	Sult1d1	3.29E-05	0.06749397

List 1 continued

ENSMUSG00000029304	Spp1	8.01E-10	19.91233169
ENSMUSG00000029322	Plac8	1.14E-20	6.911120271
ENSMUSG00000029335	Bmp3	2.30E-15	0.218735336
ENSMUSG00000029371	Cxcl5	0.00027586	25.58258012
ENSMUSG00000029377	Ereg	5.90E-15	29.18604353
ENSMUSG00000029379	Cxcl3	1.08E-24	1088.523538
ENSMUSG00000029380	Cxcl1	8.74E-62	118.7823175
ENSMUSG00000029414	Kntc1	5.77E-12	6.141112633
ENSMUSG00000029417	Cxcl9	5.53E-08	99.62982301
ENSMUSG00000029438	Bcl7a	5.51E-18	0.232412664
ENSMUSG00000029490	Mfsd7a	1.26E-21	6.266072295
ENSMUSG00000029561	Oasl2	0.001206106	5.792639519
ENSMUSG00000029596	Sdsl	0.001169209	4.6657866
ENSMUSG00000029608	Rph3a	0.000167962	35.4476409
ENSMUSG00000029657	Hsph1	0.005706598	0.229450914
ENSMUSG00000029664	Tfpi2	5.01E-07	4.098583196
ENSMUSG00000029699	Ssc4d	3.02E-05	4.518858312
ENSMUSG00000029727	Cyp3a13	1.89E-09	0.243886357
ENSMUSG00000029771	Irf5	7.17E-15	4.039816773
ENSMUSG00000029816	Gpnmb	1.23E-36	25.93359598
ENSMUSG00000029819	Npy	0.000201484	76.13465475
ENSMUSG00000029843	Slc13a4	8.28E-09	0.129408724
ENSMUSG00000029915	Clec5a	3.50E-141	62.39076324
ENSMUSG00000030048	Gkn3	6.80E-06	0.052958704
ENSMUSG00000030069	Prok2	7.08E-11	454.7258137
ENSMUSG00000030077	Chl1	4.86E-12	69.8476726
ENSMUSG00000030087	Klf15	1.07E-19	0.205281152
ENSMUSG00000030103	Bhlhe40	4.63E-29	7.534990036
ENSMUSG00000030108	Slc6a13	2.69E-11	99.7393417
ENSMUSG00000030109	Slc6a12	1.39E-20	215.3147297
ENSMUSG00000030111	A2m	0.000245003	8.884244638
ENSMUSG00000030117	Gdf3	0.000327057	22.31254788
ENSMUSG00000030125	Lrrc23	4.45E-13	0.212705449
ENSMUSG00000030142	Clec4e	2.24E-128	241.3105668
ENSMUSG00000030144	Clec4d	4.92E-75	96.47048929
ENSMUSG00000030147	Clec4b1	0.001852053	0.192098897
ENSMUSG00000030148	Clec4a2	9.26E-13	4.914212721
ENSMUSG00000030155	Clec2e	0.003074613	5.802285472
ENSMUSG00000030162	Olr1	6.54E-33	9.269503837
ENSMUSG00000030187	Klra2	1.20E-13	4.491968873
ENSMUSG00000030268	Bcat1	0.000133207	15.03273861
ENSMUSG00000030278	Cidec	1.78E-18	0.069135367
ENSMUSG00000030302	Atp2b2	2.47E-13	0.107286349
ENSMUSG00000030306	Tmtc1	1.43E-15	0.232040167
ENSMUSG00000030399	Ckm	6.86E-06	0.009655962
ENSMUSG00000030402	Ppm1n	1.15E-06	23.13707331
ENSMUSG00000030427	Lilra6	1.12E-26	10.17488458
ENSMUSG00000030470	Csrp3	5.33E-12	0.051141131
ENSMUSG00000030472	Ceacam18	1.46E-06	14.80924533
ENSMUSG00000030474	Siglece	6.58E-30	10.14881666
ENSMUSG00000030483	Cyp2b10	2.82E-17	0.200231753
ENSMUSG00000030495	Slc7a10	5.12E-15	0.116617817
ENSMUSG00000030544	Mesp1	0.004528011	0.170711587
ENSMUSG00000030546	Plin1	2.81E-05	0.070892409
ENSMUSG00000030553	Pgpep1l	0.002418338	0.062922163
ENSMUSG00000030554	Synm	1.31E-32	0.146179568
ENSMUSG00000030579	Tyrobp	1.22E-29	6.518839711
ENSMUSG00000030657	Xylt1	3.07E-26	8.477265743
ENSMUSG00000030737	Slco2b1	3.10E-08	0.249515092
ENSMUSG00000030747	Dgat2	0.009450761	4.063370941
ENSMUSG00000030786	Itgam	8.90E-44	19.8864588
ENSMUSG00000030793	Pycard	1.02E-23	4.481980248
ENSMUSG00000030895	Hpx	2.11E-09	6.149220216
ENSMUSG00000031027	Stk33	1.55E-06	0.212570891

List 1 continued

ENSMUSG00000031098	Syt8	3.01E-05	10.12149611
ENSMUSG00000031104	Rab33a	0.000617015	11.73471111
ENSMUSG00000031147	Magix	2.38E-19	0.131480765
ENSMUSG00000031163	Glod5	0.000572794	19.14692167
ENSMUSG00000031173	Otc	0.004243372	0.157015258
ENSMUSG00000031266	Gla	5.06E-40	10.19287665
ENSMUSG00000031283	Chrdl1	1.92E-08	0.199835219
ENSMUSG00000031289	Il13ra2	0.001696974	37.5471423
ENSMUSG00000031294	D630029K05Rik	8.61E-05	0.154713002
ENSMUSG00000031312	Itgb1bp2	0.001610977	0.121308415
ENSMUSG00000031444	F10	8.34E-95	51.78242703
ENSMUSG00000031461	Myom2	3.98E-05	0.104289638
ENSMUSG00000031489	Adrb3	4.48E-09	0.178457755
ENSMUSG00000031494	Cd209a	0.000561054	0.103935522
ENSMUSG00000031504	Rab20	2.17E-43	10.57573882
ENSMUSG00000031551	Ido1	0.002754801	11.27803406
ENSMUSG00000031554	Adam5	0.000137313	0.058030505
ENSMUSG00000031562	Dctd	1.53E-05	5.85507953
ENSMUSG00000031584	Gsr	1.40E-20	5.561973503
ENSMUSG00000031594	Fgl1	0.000429282	5.998031585
ENSMUSG00000031596	Slc7a2	6.30E-29	5.314591755
ENSMUSG00000031610	Scrg1	4.64E-05	7.715872038
ENSMUSG00000031613	Hpgd	2.97E-15	0.23463581
ENSMUSG00000031662	Snx20	1.12E-53	8.84381917
ENSMUSG00000031725	Ces1f	6.29E-21	0.014239424
ENSMUSG00000031762	Mt2	3.42E-13	21.91316661
ENSMUSG00000031765	Mt1	5.59E-26	8.872498196
ENSMUSG00000031775	Pllp	2.02E-19	0.143497355
ENSMUSG00000031786	Drc7	2.26E-11	0.246018508
ENSMUSG00000031821	Gins2	7.31E-11	4.265607066
ENSMUSG00000031827	Cotl1	5.85E-19	4.077493067
ENSMUSG00000031831	Dnaaf1	2.50E-08	0.228156126
ENSMUSG00000031844	Hsd17b2	0.002286433	15.80555117
ENSMUSG00000031870	Pgr	2.28E-11	0.095675478
ENSMUSG00000031872	Bean1	0.004025695	4.531305873
ENSMUSG00000031881	Cdh16	1.31E-10	0.201810819
ENSMUSG00000031891	Hsd11b2	5.12E-08	6.314864393
ENSMUSG00000031966	Glb1l3	6.54E-05	0.119032399
ENSMUSG00000032000	Birc3	1.22E-25	5.106268964
ENSMUSG00000032024	Clmp	5.20E-21	6.28623898
ENSMUSG00000032064	Dixdc1	5.04E-16	0.213359191
ENSMUSG00000032122	Slc37a2	4.07E-35	7.21874537
ENSMUSG00000032204	Aqp9	2.67E-13	6.929216797
ENSMUSG00000032224	Fam81a	1.95E-09	0.244620722
ENSMUSG00000032246	Calml4	9.64E-09	0.208215121
ENSMUSG00000032281	Acsbg1	2.52E-12	7.414628511
ENSMUSG00000032294	Pkm	8.81E-28	5.573951818
ENSMUSG00000032300	1700017B05Rik	2.22E-24	8.381460424
ENSMUSG00000032315	Cyp1a1	0.002869363	0.080778694
ENSMUSG00000032322	Pstpip1	1.01E-29	5.241073208
ENSMUSG00000032357	Tinag	2.26E-09	0.229682697
ENSMUSG00000032487	Ptgs2	5.53E-12	6.263341608
ENSMUSG00000032511	Scn5a	3.10E-12	0.181677526
ENSMUSG00000032532	Cck	0.000244202	71.39567193
ENSMUSG00000032643	Fhl3	1.22E-13	4.717038745
ENSMUSG00000032649	Colgalt2	9.55E-22	0.181467178
ENSMUSG00000032661	Oas3	1.89E-08	16.74428331
ENSMUSG00000032688	Malt1	2.90E-70	9.701501177
ENSMUSG00000032690	Oas2	0.002273698	4.955600021
ENSMUSG00000032691	Nlrp3	4.94E-63	30.45240139
ENSMUSG00000032719	Sbspon	0.000525424	0.086978254
ENSMUSG00000032724	Abtb2	7.97E-25	5.588517949
ENSMUSG00000032802	Srxn1	2.87E-12	4.616512665
ENSMUSG00000032845	Alpk2	6.00E-05	7.966044077

List 1 continued

ENSMUSG00000032854	Ugt8a	0.000219217	0.166205073
ENSMUSG00000032860	P2ry2	5.71E-19	4.30061048
ENSMUSG00000032925	Itgbl1	7.54E-10	0.105203708
ENSMUSG00000032942	Ucp3	4.92E-08	0.108652559
ENSMUSG00000033152	Podxl2	8.54E-07	0.19896431
ENSMUSG00000033192	Lpcat2	7.62E-63	14.89233309
ENSMUSG00000033207	Mamdc2	6.95E-19	0.158833594
ENSMUSG00000033213	AA467197	3.39E-206	164.3650773
ENSMUSG00000033307	Mif	1.65E-15	4.318816235
ENSMUSG00000033355	Rtp4	0.005478424	4.161128703
ENSMUSG00000033508	Asprv1	2.12E-17	140.8372685
ENSMUSG00000033538	Casp4	1.02E-43	10.09162136
ENSMUSG00000033578	Tmem35a	6.43E-06	0.164441955
ENSMUSG00000033595	Lgi3	7.95E-16	0.149150848
ENSMUSG00000033644	Piwil2	8.17E-29	32.1708933
ENSMUSG00000033731	3300002A11Rik	3.10E-07	0.178312722
ENSMUSG00000033763	Mtss1l	1.32E-15	0.212103285
ENSMUSG00000033768	Nrxn2	2.65E-12	0.20404291
ENSMUSG00000033777	Tlr13	1.84E-45	25.31957004
ENSMUSG00000033860	Fgg	1.25E-05	4.765696926
ENSMUSG00000033948	Zswim5	3.29E-09	0.217618212
ENSMUSG00000034116	Vav1	2.47E-71	10.12371168
ENSMUSG00000034226	Rhov	4.68E-32	18.09076045
ENSMUSG00000034245	Hdac11	2.33E-15	0.226803068
ENSMUSG00000034248	Slc25a37	6.30E-29	5.314210992
ENSMUSG00000034295	Fhod3	0.00030915	0.17400262
ENSMUSG00000034361	Cpne2	5.96E-14	5.940990777
ENSMUSG00000034391	Fbxo15	0.000730854	5.85726711
ENSMUSG00000034394	Lif	1.08E-14	7.172093688
ENSMUSG00000034416	Pkd1l2	7.41E-06	15.17779501
ENSMUSG00000034460	Six4	1.54E-11	0.183052743
ENSMUSG00000034467	Dynlrb2	1.85E-10	0.245907243
ENSMUSG00000034593	Myo5a	7.51E-25	4.078122121
ENSMUSG00000034641	Cd300ld	1.78E-16	6.739339195
ENSMUSG00000034648	Lrrn1	0.006609371	12.63832671
ENSMUSG00000034652	Cd300a	3.03E-14	4.928472124
ENSMUSG00000034765	Dusp5	2.31E-14	4.517519213
ENSMUSG00000034810	Scn7a	2.62E-16	0.221680797
ENSMUSG00000034825	Nrip3	2.88E-06	7.03942618
ENSMUSG00000034855	Cxcl10	5.60E-11	178.795138
ENSMUSG00000034987	Hrh2	6.58E-15	7.056675961
ENSMUSG00000035004	Igsf6	4.80E-67	14.77859865
ENSMUSG00000035105	Egln3	3.06E-08	18.64597878
ENSMUSG00000035186	Ubd	1.76E-11	150.9277132
ENSMUSG00000035208	Slfn8	4.75E-05	8.353439853
ENSMUSG00000035258	Abi3bp	2.43E-16	0.196486207
ENSMUSG00000035296	Sgcg	2.85E-05	0.116100079
ENSMUSG00000035352	Ccl12	4.38E-30	22.2673239
ENSMUSG00000035356	Nfkbiz	5.74E-39	7.4745814
ENSMUSG00000035373	Ccl7	5.09E-14	84.49006544
ENSMUSG00000035385	Ccl2	9.08E-109	202.4628751
ENSMUSG00000035407	Kank4	2.43E-25	0.10803296
ENSMUSG00000035458	Tnni3	0.000204106	0.220270425
ENSMUSG00000035486	Plk5	1.11E-06	0.086158371
ENSMUSG00000035493	Tgfbi	1.58E-86	12.45114771
ENSMUSG00000035671	Zswim4	8.80E-14	4.726735126
ENSMUSG00000035673	Sbno2	5.02E-24	4.452589037
ENSMUSG00000035686	Thrsp	8.85E-12	0.07027376
ENSMUSG00000035692	Isg15	0.001002914	11.70461549
ENSMUSG00000035860	Cdhr3	8.77E-12	0.224517793
ENSMUSG00000035910	Dcdc2a	1.01E-13	0.073129947
ENSMUSG00000035916	Ptprq	0.001073726	7.027501736
ENSMUSG00000036062	Phf24	2.31E-12	0.222831458
ENSMUSG00000036067	Slc2a6	4.71E-41	15.05415148

List 1 continued

ENSMUSG00000036095	Dgkb	0.000569245	0.123814598
ENSMUSG00000036223	Ska1	0.006844642	4.283133654
ENSMUSG00000036295	Lrrn3	2.88E-11	0.21014148
ENSMUSG00000036362	P2ry13	2.51E-23	6.576585958
ENSMUSG00000036395	Glb1l2	1.18E-19	0.17764396
ENSMUSG00000036412	Arsi	1.35E-06	4.894434983
ENSMUSG00000036469	1-Mar	1.44E-27	4.9659905
ENSMUSG00000036478	Btg1	5.14E-21	4.339214032
ENSMUSG00000036585	Fgf1	2.62E-26	0.180907165
ENSMUSG00000036813	Entpd8	0.001365346	0.108042671
ENSMUSG00000036820	Amdhd2	2.01E-18	4.536570229
ENSMUSG00000036833	Pnpla7	8.41E-16	4.375620111
ENSMUSG00000036931	Nfkbid	1.87E-41	14.39227709
ENSMUSG00000037014	Sstr4	0.000179108	0.215178099
ENSMUSG00000037016	Frem2	3.21E-05	0.180791838
ENSMUSG00000037053	Azgp1	1.60E-12	0.123645645
ENSMUSG00000037095	Lrg1	6.38E-44	6.498440989
ENSMUSG00000037101	Ttc29	1.09E-10	0.193298457
ENSMUSG00000037259	Dzank1	1.33E-08	0.185106101
ENSMUSG00000037321	Tap1	1.25E-08	4.561246096
ENSMUSG00000037362	Nov	1.85E-05	0.232897819
ENSMUSG00000037411	Serpine1	1.33E-17	5.187831222
ENSMUSG00000037451	Slc22a20	0.005578223	11.78952592
ENSMUSG00000037477	Tbx10	0.00096279	71.21764626
ENSMUSG00000037492	Zmat4	0.000305685	0.109756143
ENSMUSG00000037541	Shank2	1.24E-08	0.228991744
ENSMUSG00000037568	Vash2	0.00306949	4.004323943
ENSMUSG00000037613	Tnfrsf23	3.93E-44	23.59447379
ENSMUSG00000037624	Kcnk2	3.76E-21	0.238515511
ENSMUSG00000037731	Themis2	6.36E-15	4.356444724
ENSMUSG00000037771	Slc32a1	0.000332876	12.50054815
ENSMUSG00000037801	Iqch	0.006325705	0.102511154
ENSMUSG00000037846	Rtkn2	5.40E-17	0.147690713
ENSMUSG00000037860	Aim2	8.96E-15	4.533493182
ENSMUSG00000037868	Egr2	9.50E-27	6.04936612
ENSMUSG00000037872	Ackr1	1.79E-09	21.34661068
ENSMUSG00000037921	Ddx60	0.002513878	5.100094248
ENSMUSG00000037946	Fgd3	1.49E-24	7.355458122
ENSMUSG00000037960	Card19	2.18E-17	4.378285051
ENSMUSG00000037966	Ninj1	1.89E-56	12.36496983
ENSMUSG00000037973	Ccdc129	1.27E-20	0.075755043
ENSMUSG00000038011	Dnah10	2.00E-08	0.22932067
ENSMUSG00000038037	Socs1	8.13E-05	9.37668876
ENSMUSG00000038060	Dlec1	5.37E-08	0.183689112
ENSMUSG00000038065	Mturn	1.03E-35	0.143030441
ENSMUSG00000038067	Csf3	5.56E-30	158.6740026
ENSMUSG00000038077	Kcna6	0.000823026	0.018288711
ENSMUSG00000038147	Cd84	1.80E-27	5.284747369
ENSMUSG00000038168	P3h2	6.38E-13	0.236466922
ENSMUSG00000038179	Slamf7	6.60E-09	5.689010775
ENSMUSG00000038292	Ccdc155	5.94E-05	0.218122189
ENSMUSG00000038301	Snx10	3.33E-50	7.612178133
ENSMUSG00000038352	Arl5c	4.42E-11	4.052507441
ENSMUSG00000038370	Pcp4l1	9.51E-19	0.248005728
ENSMUSG00000038418	Egr1	7.85E-13	5.546206731
ENSMUSG00000038508	Gdf15	3.23E-34	10.88876693
ENSMUSG00000038527	C1rl	4.20E-28	5.352325569
ENSMUSG00000038572	Bpifb5	1.08E-09	0.130697646
ENSMUSG00000038642	Ctss	3.07E-22	4.898253066
ENSMUSG00000038663	Fsd2	2.14E-06	0.231457691
ENSMUSG00000038756	Ttll6	1.53E-08	0.104235828
ENSMUSG00000038791	Scgb3a2	6.31E-49	0.084987913
ENSMUSG00000038801	Scgb1c1	1.34E-08	0.239980296
ENSMUSG00000038843	Gcnt1	7.53E-17	5.586043687

List 1 continued

ENSMUSG00000038963	Slco4a1	5.21E-13	5.889260284
ENSMUSG00000039058	Ak5	0.000316114	6.059486985
ENSMUSG00000039084	Chad	9.58E-19	0.131853751
ENSMUSG00000039099	Wdr93	0.000345474	0.240292734
ENSMUSG00000039109	F13a1	3.67E-07	13.71611494
ENSMUSG00000039114	Nrn1	2.62E-19	0.141274651
ENSMUSG00000039155	Cdh26	0.004188954	0.138428111
ENSMUSG00000039196	Orm1	1.18E-129	339.8874892
ENSMUSG00000039208	Metrnl	5.30E-14	4.53373187
ENSMUSG00000039232	Stx11	4.01E-11	4.601780073
ENSMUSG00000039257	Vstm2b	3.62E-05	0.048045778
ENSMUSG00000039323	Igfbp2	7.44E-13	0.189520099
ENSMUSG00000039364	Sectm1b	0.000202811	31.11449631
ENSMUSG00000039395	Mreg	3.68E-14	31.9637136
ENSMUSG00000039452	Snx22	4.12E-09	0.168998931
ENSMUSG00000039519	Cyp7b1	2.67E-39	11.18548868
ENSMUSG00000039521	Foxp3	1.06E-05	5.555426819
ENSMUSG00000039543	Cfap70	3.17E-09	0.226403547
ENSMUSG00000039552	Rsph4a	3.62E-10	0.247786076
ENSMUSG00000039628	Hs3st6	6.56E-05	0.229341369
ENSMUSG00000039672	Kcne2	6.28E-29	0.154386021
ENSMUSG00000039720	Got1l1	0.003562906	0.087640234
ENSMUSG00000039753	Fbxl5	1.58E-23	4.297908244
ENSMUSG00000039813	Tbc1d2	2.45E-27	4.234543882
ENSMUSG00000039883	Lrrc17	0.000616637	0.243811091
ENSMUSG00000040017	Saa4	8.26E-08	207.0149271
ENSMUSG00000040026	Saa3	0	2579.781229
ENSMUSG00000040037	Negr1	4.54E-07	0.114046755
ENSMUSG00000040133	Gpr176	1.33E-13	9.993582901
ENSMUSG00000040181	Fmo1	7.44E-24	0.222718782
ENSMUSG00000040298	Btbd16	4.77E-07	130.4019429
ENSMUSG00000040328	Olfr56	1.32E-05	13.46455861
ENSMUSG00000040488	Ltbp4	9.84E-14	0.160151042
ENSMUSG00000040528	Milr1	2.64E-30	7.116820768
ENSMUSG00000040537	Adam22	7.33E-10	0.178473882
ENSMUSG00000040543	Pitpnm3	1.03E-12	0.193850577
ENSMUSG00000040552	C3ar1	2.68E-39	11.81924819
ENSMUSG00000040653	Ppp1r14c	4.94E-13	0.235154052
ENSMUSG00000040659	Efhd2	1.34E-21	4.303721775
ENSMUSG00000040666	Sh3bgr	2.72E-05	0.232255834
ENSMUSG00000040703	Cyp2s1	8.52E-19	0.242861265
ENSMUSG00000040711	Sh3pxd2b	8.29E-36	6.30501831
ENSMUSG00000040740	Slc25a34	0.006894436	0.217500992
ENSMUSG00000040747	Cd53	2.35E-47	10.12347324
ENSMUSG00000040752	Myh6	0.000241843	0.10771726
ENSMUSG00000040808	S100g	8.01E-18	0.172678564
ENSMUSG00000040838	Gm11639	1.00E-05	93.57545051
ENSMUSG00000040855	Reps2	3.12E-13	0.225803113
ENSMUSG00000040896	Kcnd3	0.000233464	0.179263474
ENSMUSG00000040938	Slc16a11	1.55E-13	0.200138078
ENSMUSG00000040978	Gm11992	1.45E-05	0.239955892
ENSMUSG00000040998	Npnt	2.53E-16	0.232989316
ENSMUSG00000041202	Pla2g2d	0.000227233	0.161455174
ENSMUSG00000041323	Ak7	1.90E-07	0.243070733
ENSMUSG00000041324	Inhba	2.04E-157	121.9329236
ENSMUSG00000041347	Bdkrb1	4.69E-07	5.093577732
ENSMUSG00000041423	Paqr6	2.61E-05	0.226259601
ENSMUSG00000041449	Serpina3h	1.22E-10	7.886129271
ENSMUSG00000041481	Serpina3g	1.45E-19	12.42020182
ENSMUSG00000041523	Upk2	0.001276687	0.088080358
ENSMUSG00000041577	Prelp	8.16E-24	0.245166434
ENSMUSG00000041608	Entpd3	6.76E-06	4.490205681
ENSMUSG00000041624	Gucy1a2	1.84E-06	0.231400058
ENSMUSG00000041644	Slc5a12	1.29E-15	0.067321523

List 1 continued

ENSMUSG00000041754	Trem3	2.87E-31	14.03368771
ENSMUSG00000041827	Oasl1	1.49E-05	16.86862713
ENSMUSG00000041828	Abca8a	7.14E-07	0.09744596
ENSMUSG00000041842	Fhdc1	2.55E-19	0.231049158
ENSMUSG00000041930	Fam222a	6.10E-09	0.222955388
ENSMUSG00000041984	Rptn	0.007756296	0.14353802
ENSMUSG00000042109	Csdc2	6.37E-06	0.166999964
ENSMUSG00000042254	Cilp	5.22E-05	0.18413199
ENSMUSG00000042265	Trem1	1.43E-34	30.16019343
ENSMUSG00000042269	Fam92b	7.12E-09	0.219081471
ENSMUSG00000042306	S100a14	1.44E-17	5.36093607
ENSMUSG00000042349	Ikbke	1.72E-60	14.93557055
ENSMUSG00000042357	Gjb5	6.04E-07	6.742242384
ENSMUSG00000042359	Osbpl6	6.17E-24	0.176125736
ENSMUSG00000042428	Mgat3	1.13E-18	0.239692692
ENSMUSG00000042433	Pih1h3b	0.00181872	0.226866654
ENSMUSG00000042529	Kcnj12	6.86E-05	0.197216587
ENSMUSG00000042607	Asb4	1.11E-05	7.186961638
ENSMUSG00000042638	Gucy2c	5.51E-06	24.73971692
ENSMUSG00000042677	Zc3h12a	4.18E-42	8.245244821
ENSMUSG00000042707	Dnali1	8.66E-19	0.195140988
ENSMUSG00000042750	Bex2	9.07E-11	0.153209108
ENSMUSG00000042759	Apobr	1.79E-33	6.596238347
ENSMUSG00000042793	Lgr6	2.70E-07	0.148347672
ENSMUSG00000042895	Abra	0.000407372	0.175730743
ENSMUSG00000043091	Tuba1c	1.54E-16	4.594908828
ENSMUSG00000043122	A530016L24Rik	0.001226696	0.02505651
ENSMUSG00000043157	Arl11	2.97E-17	6.430109722
ENSMUSG00000043230	Fam124b	0.00013873	0.201542513
ENSMUSG00000043263	Ifi209	0.008130874	4.253619333
ENSMUSG00000043333	Rhbdl2	0.000204594	7.457430182
ENSMUSG00000043383	Olfr1342	0.009003851	0.153932791
ENSMUSG00000043496	Tril	2.05E-22	0.123317141
ENSMUSG00000043613	Mmp3	5.00E-28	4.538199291
ENSMUSG00000043631	Ecm2	1.33E-12	0.14508143
ENSMUSG00000043635	Adamts3	7.44E-05	4.604960257
ENSMUSG00000043705	Capn13	0.000897689	0.082852402
ENSMUSG00000043740	B430306N03Rik	1.72E-44	16.12684456
ENSMUSG00000043939	A530064D06Rik	9.84E-15	7.014311983
ENSMUSG00000043953	Ccrl2	7.88E-56	7.891245465
ENSMUSG00000044062	Plekhd1os	0.0057371	0.199674003
ENSMUSG00000044103	Il1f9	2.50E-25	87.98827565
ENSMUSG00000044156	Hepacam2	4.12E-10	0.109646117
ENSMUSG00000044162	Tnip3	7.00E-24	8.535845872
ENSMUSG00000044165	Bcl2l15	0.002943268	34.49678741
ENSMUSG00000044244	Il20rb	5.28E-08	4.560166123
ENSMUSG00000044296	Zfp879	0.003757747	0.239747077
ENSMUSG00000044313	Mab21l3	5.71E-19	19.04627582
ENSMUSG00000044338	Aplnr	1.64E-17	0.186126388
ENSMUSG00000044350	Lacc1	2.62E-34	7.765066808
ENSMUSG00000044352	Sowaha	0.000802532	0.18377971
ENSMUSG00000044365	Cxxc4	2.29E-06	0.11928619
ENSMUSG00000044461	Shisa2	8.96E-05	0.241855363
ENSMUSG00000044583	Tlr7	1.78E-27	5.566856379
ENSMUSG00000044694	2010007H06Rik	2.27E-06	0.219481196
ENSMUSG00000044701	Il27	7.98E-08	14.75646657
ENSMUSG00000044726	Erich5	0.003477193	0.049572166
ENSMUSG00000044811	Cd300c2	2.16E-16	4.905708874
ENSMUSG00000044827	Tlr1	6.08E-13	4.456083979
ENSMUSG00000044903	Psg22	0.00046084	49.99361793
ENSMUSG00000044951	Mylk4	2.64E-05	0.051901524
ENSMUSG00000044966	Fbxo48	0.006742596	4.000577614
ENSMUSG00000045027	Prss22	2.14E-07	18.39268603
ENSMUSG00000045036	Tmem232	2.89E-07	0.207841673

List 1 continued

ENSMUSG00000045065	9930022D16Rik	2.08E-16	88.42355775
ENSMUSG00000045201	Lrrc3b	0.002476668	0.021312817
ENSMUSG00000045273	Cenph	0.000682303	4.164926592
ENSMUSG00000045312	Lhfpl2	1.64E-34	8.129817994
ENSMUSG00000045349	Sh2d5	2.57E-06	9.724104204
ENSMUSG00000045362	Tnfrsf26	2.97E-53	25.28811422
ENSMUSG00000045381	Olfr433	0.004643729	8.296292642
ENSMUSG00000045441	Gprin3	8.27E-07	0.243893315
ENSMUSG00000045502	Hcar2	9.33E-212	138.5769777
ENSMUSG00000045514	Olfr460	0.001795713	24.64640914
ENSMUSG00000045515	Pou3f3	0.006551877	0.035810762
ENSMUSG00000045551	Fpr1	8.31E-21	12.20254813
ENSMUSG00000045763	Basp1	3.60E-43	16.2538563
ENSMUSG00000045875	Adra1a	7.29E-12	0.209171927
ENSMUSG00000045915	Ccdc42	1.38E-05	0.163779785
ENSMUSG00000045954	Cavin2	1.09E-30	0.230027205
ENSMUSG00000046031	Calhm6	7.21E-05	19.56552797
ENSMUSG00000046093	Hpcal4	1.41E-13	0.024406153
ENSMUSG00000046182	Gsg1l	2.68E-08	0.048168686
ENSMUSG00000046192	Iqub	9.62E-07	0.2408964
ENSMUSG00000046215	Rprml	2.75E-10	0.082770314
ENSMUSG00000046223	Plaur	1.64E-27	5.663748295
ENSMUSG00000046240	Hepacam	8.16E-05	0.177073433
ENSMUSG00000046242	Nme9	7.19E-15	0.199190438
ENSMUSG00000046245	Pilra	1.04E-38	9.297041219
ENSMUSG00000046610	Oacyl	5.53E-09	11.94195793
ENSMUSG00000046618	Olfml2a	4.92E-18	0.15710071
ENSMUSG00000046623	Gjb4	3.50E-09	15.05386702
ENSMUSG00000046687	Gm5424	2.21E-17	4.816595069
ENSMUSG00000046688	Tifa	2.82E-31	7.42412298
ENSMUSG00000046709	Mapk10	0.009578329	0.164491613
ENSMUSG00000046794	Ppp1r3b	1.04E-16	4.32682149
ENSMUSG00000046805	Mpeg1	1.62E-84	9.881590982
ENSMUSG00000046879	Irgm1	0.000492428	7.21794108
ENSMUSG00000046908	Ltb4r1	1.67E-27	8.300897448
ENSMUSG00000046997	Spsb4	5.28E-10	9.838507269
ENSMUSG00000047021	Cfap65	3.41E-09	0.197371919
ENSMUSG00000047104	Pbp2	0.001528864	45.96921734
ENSMUSG00000047230	Cldn2	3.29E-09	10.84735298
ENSMUSG00000047293	Gpr15	0.004437941	4.253627291
ENSMUSG00000047330	Kcne4	2.34E-26	6.726492462
ENSMUSG00000047361	Gm973	1.98E-07	0.213045755
ENSMUSG00000047407	Tgif1	7.10E-31	6.877402594
ENSMUSG00000047415	Gpr68	1.33E-09	4.421294143
ENSMUSG00000047419	Cmya5	7.53E-08	0.180750775
ENSMUSG00000047443	Erfe	2.02E-07	10.60548165
ENSMUSG00000047517	Dmbt1	3.94E-06	27.88389724
ENSMUSG00000047562	Mmp10	0.003411257	33.6517663
ENSMUSG00000047592	Nxpe5	2.36E-09	7.987501092
ENSMUSG00000047773	Ankfn1	7.62E-05	0.244273453
ENSMUSG00000047798	Cd300lf	2.41E-59	29.00270173
ENSMUSG00000047810	Ccdc88b	3.86E-39	9.982682017
ENSMUSG00000047842	Diras2	9.21E-17	0.148658547
ENSMUSG00000047945	Marcksl1	1.92E-69	18.10556067
ENSMUSG00000048038	Ccdc187	5.06E-15	0.168650469
ENSMUSG00000048040	Arxes2	1.44E-10	0.209711555
ENSMUSG00000048163	Selplg	4.42E-21	4.196567136
ENSMUSG00000048234	Rnf149	3.11E-41	13.12472412
ENSMUSG00000048368	Omd	2.79E-05	0.220021498
ENSMUSG00000048480	Cxcr1	6.01E-21	17.51575518
ENSMUSG00000048621	Gm6377	7.42E-20	11.36624798
ENSMUSG00000048636	A730049H05Rik	6.71E-07	8.021468634
ENSMUSG00000048721	Fndc9	2.14E-05	7.227722166
ENSMUSG00000048752	Prss50	0.00715867	4.236749485

List 1 continued

ENSMUSG00000048779	P2ry6	1.35E-20	4.69155845
ENSMUSG00000048794	Cfap100	2.46E-09	0.236404824
ENSMUSG00000048834	Vstm2a	2.26E-06	0.116550481
ENSMUSG00000049037	Clec4a1	2.42E-17	4.627917219
ENSMUSG00000049130	C5ar1	5.24E-33	10.20852667
ENSMUSG00000049154	Fam183b	3.64E-11	0.238182561
ENSMUSG00000049303	Syt12	2.02E-14	9.369363318
ENSMUSG00000049511	Htr1b	2.86E-07	0.119512462
ENSMUSG00000049526	Tmem202	0.000631548	4.115979817
ENSMUSG00000049537	Tecrl	0.002371432	0.247163359
ENSMUSG00000049555	Tmie	2.69E-11	0.163595375
ENSMUSG00000049653	Spatc1	0.00375636	8.84618821
ENSMUSG00000049719	Prss46	4.82E-05	70.36053199
ENSMUSG00000049723	Mmp12	1.93E-12	41.23436247
ENSMUSG00000049848	Ceacam19	0.006330621	8.934243284
ENSMUSG00000049988	Lrrc25	2.58E-31	9.127042402
ENSMUSG00000050089	Akap4	7.02E-09	154.155024
ENSMUSG00000050097	Ces2b	6.03E-06	0.130295888
ENSMUSG00000050103	Agmo	7.56E-13	0.18343753
ENSMUSG00000050138	Kcnk12	1.78E-08	0.196140458
ENSMUSG00000050222	Il17d	5.76E-12	0.239806508
ENSMUSG00000050335	Lgals3	3.72E-31	6.653885555
ENSMUSG00000050359	Sprr1a	8.12E-08	10.12654822
ENSMUSG00000050370	Ch25h	2.33E-22	14.69917653
ENSMUSG00000050395	Tnfsf15	1.52E-23	10.28317936
ENSMUSG00000050439	Enthd1	4.49E-11	474.4270426
ENSMUSG00000050447	Lypd6	0.000219429	0.112363009
ENSMUSG00000050493	Fam167b	0.000595798	4.442369756
ENSMUSG00000050578	Mmp13	3.37E-25	20.29990493
ENSMUSG00000050721	Plekho2	1.96E-50	5.952041508
ENSMUSG00000050737	Ptges	1.10E-28	7.178014443
ENSMUSG00000050844	1700020N01Rik	0.002197206	24.95410833
ENSMUSG00000050896	Rtn4rl2	2.56E-20	6.204235787
ENSMUSG00000050908	Tvp23a	3.02E-05	7.540378755
ENSMUSG00000050931	Sgms2	1.19E-25	9.891391024
ENSMUSG00000050957	Insl6	0.000214893	5.136598117
ENSMUSG00000050966	Lin28a	1.97E-05	10.14026938
ENSMUSG00000050967	Creg2	1.34E-11	10.03523711
ENSMUSG00000051048	P4ha3	1.95E-05	4.525089359
ENSMUSG00000051367	Six1	4.45E-07	0.194444467
ENSMUSG00000051439	Cd14	9.47E-48	32.52969399
ENSMUSG00000051498	Tlr6	9.10E-39	15.85278282
ENSMUSG00000051590	Map3k19	1.12E-07	0.2067945
ENSMUSG00000051606	2010001K21Rik	7.00E-09	0.220005761
ENSMUSG00000051682	Treml4	1.11E-21	8.484072878
ENSMUSG00000051726	Kcnf1	0.000884772	0.124000212
ENSMUSG00000051747	Ttn	2.17E-06	0.189799951
ENSMUSG00000051748	Wfdc21	8.41E-27	16.18054827
ENSMUSG00000051832	E230016K23Rik	5.20E-05	5.757620461
ENSMUSG00000051839	Gypa	0.001398362	0.061527622
ENSMUSG00000051906	Cd209f	1.36E-06	0.076787615
ENSMUSG00000052102	Gnpda1	6.60E-17	4.646664309
ENSMUSG00000052131	Akr1b7	9.26E-05	0.065382372
ENSMUSG00000052188	Gm14964	4.06E-10	0.140064342
ENSMUSG00000052212	Cd177	1.73E-23	12.64385641
ENSMUSG00000052270	Fpr2	9.85E-46	22.71223892
ENSMUSG00000052353	Cemip	3.31E-10	8.806747091
ENSMUSG00000052435	Cebpe	1.88E-07	6.908044706
ENSMUSG00000052698	Tln2	8.85E-17	0.201070587
ENSMUSG00000052749	Trim30b	8.03E-15	8.037264551
ENSMUSG00000052776	Oas1a	0.000328537	5.606946977
ENSMUSG00000052837	Junb	9.59E-40	7.718282616
ENSMUSG00000052861	Dnah6	1.36E-09	0.21067278
ENSMUSG00000052974	Cyp2f2	3.72E-37	0.065921921

List 1 continued

ENSMUSG00000053093	Myh7	4.72E-17	0.188039063
ENSMUSG00000053101	Gpr141	1.20E-65	12.68313727
ENSMUSG00000053111	Fank1	1.50E-05	0.247905766
ENSMUSG00000053113	Socs3	1.28E-79	10.99452165
ENSMUSG00000053161	Daw1	0.000682899	0.182708394
ENSMUSG00000053175	Bcl3	1.58E-17	4.430565758
ENSMUSG00000053214	Gm9899	0.000903813	0.234465965
ENSMUSG00000053279	Aldh1a1	4.18E-38	0.139655523
ENSMUSG00000053318	Slamf8	7.73E-08	21.19083721
ENSMUSG00000053338	Tarm1	1.68E-58	107.2002244
ENSMUSG00000053442	4930597O21Rik	0.009506524	28.90848827
ENSMUSG00000053475	Tnfaip6	1.17E-05	4.947243573
ENSMUSG00000053647	Gper1	8.88E-05	0.201738905
ENSMUSG00000054072	Iigp1	0.002325063	10.28715664
ENSMUSG00000054146	Krt15	0.000370582	0.033706211
ENSMUSG00000054169	Ceacam10	1.21E-08	15.82935727
ENSMUSG00000054203	Ifi205	4.80E-06	18.37378853
ENSMUSG00000054252	Fgfr3	8.59E-16	0.219354915
ENSMUSG00000054320	Lrrc36	4.40E-06	0.238123786
ENSMUSG00000054422	Fabp1	3.30E-20	0.023984029
ENSMUSG00000054459	Vsnl1	9.01E-07	0.236074176
ENSMUSG00000054477	Kcnn2	1.90E-05	0.149418698
ENSMUSG00000054520	Sh3bp2	1.15E-49	6.662734482
ENSMUSG00000054556	Gm4876	0.00047324	0.200219757
ENSMUSG00000054619	Mettl7a1	5.63E-37	0.102317165
ENSMUSG00000054855	Rnd1	2.83E-12	4.206844444
ENSMUSG00000054863	Fam19a5	0.007935706	0.207279808
ENSMUSG00000054905	Stfa3	5.49E-18	322.9239368
ENSMUSG00000054931	Zkscan4	2.62E-05	0.228260384
ENSMUSG00000054986	Sec14l3	1.18E-26	0.201340829
ENSMUSG00000055134	9130017K11Rik	0.002838304	0.114070069
ENSMUSG00000055170	Ifng	5.81E-08	26.07460455
ENSMUSG00000055301	Adh7	9.71E-23	0.148688434
ENSMUSG00000055368	Slc6a2	1.40E-11	0.225910832
ENSMUSG00000055430	Nap1l5	0.006899193	0.153661197
ENSMUSG00000055493	Epm2a	8.52E-12	0.216168967
ENSMUSG00000055541	Lair1	4.23E-21	7.36898661
ENSMUSG00000055629	B4galnt4	5.44E-06	5.649988324
ENSMUSG00000055676	Gm5069	0.001072296	0.243775468
ENSMUSG00000055733	Nap1l3	1.50E-05	0.223796461
ENSMUSG00000055805	Fmnl1	1.80E-21	4.018090791
ENSMUSG00000055976	Cldn23	1.59E-06	0.207395857
ENSMUSG00000055994	Nod2	6.18E-14	5.488963417
ENSMUSG00000056054	S100a8	1.33E-08	22.40912127
ENSMUSG00000056071	S100a9	1.23E-07	15.03851638
ENSMUSG00000056313	Tcim	4.63E-16	0.244170353
ENSMUSG00000056413	Adap1	2.36E-20	4.152415147
ENSMUSG00000056476	Med12l	4.30E-09	4.607567602
ENSMUSG00000056501	Cebpb	7.22E-33	5.250149874
ENSMUSG00000056508	1700001K19Rik	0.007736482	0.242304234
ENSMUSG00000056529	Ptafr	8.82E-150	50.60019052
ENSMUSG00000056569	Mpz	1.86E-05	0.181759836
ENSMUSG00000056737	Capg	1.71E-44	7.566459485
ENSMUSG00000056749	Nfil3	1.14E-16	6.677783513
ENSMUSG00000056824	Zfp663	0.001913504	0.10697289
ENSMUSG00000056973	Ces1d	6.79E-25	0.15193868
ENSMUSG00000057060	Slc35f3	1.34E-06	0.10471387
ENSMUSG00000057074	Ces1g	1.33E-19	0.061649057
ENSMUSG00000057135	Scimp	5.51E-15	6.436652407
ENSMUSG00000057182	Scn3a	6.83E-19	0.13966351
ENSMUSG00000057191	AB124611	2.30E-32	5.232883525
ENSMUSG00000057265	Bbof1	1.45E-06	0.244638914
ENSMUSG00000057286	St6galnac2	1.97E-17	0.245559608
ENSMUSG00000057346	Apol9a	6.02E-05	4.616692063

List 1 continued

ENSMUSG00000057378	Ryr3	0.002856392	0.14700545
ENSMUSG00000057465	Saa2	1.50E-14	390.6209695
ENSMUSG00000057606	Colq	1.17E-46	0.02647835
ENSMUSG00000057715	A830018L16Rik	0.000226484	0.22779101
ENSMUSG00000057716	Tmem178b	0.005811587	0.120634507
ENSMUSG00000057897	Camk2b	1.63E-05	0.228128263
ENSMUSG00000057933	Gsta2	2.19E-17	0.048290108
ENSMUSG00000058046	4933430I17Rik	0.0017542	5.15118999
ENSMUSG00000058057	Mettl7a3	0.00559868	0.051791853
ENSMUSG00000058145	Adamts17	1.16E-05	0.234186932
ENSMUSG00000058159	T2	0.002795458	0.099436358
ENSMUSG00000058427	Cxcl2	5.98E-33	3289.780123
ENSMUSG00000058624	Gda	1.02E-30	6.891348411
ENSMUSG00000058715	Fcer1g	1.36E-58	11.21108856
ENSMUSG00000058755	Osm	1.93E-43	50.58503118
ENSMUSG00000058818	Pirb	3.30E-35	7.259812016
ENSMUSG00000058869	Gm4832	1.68E-06	456.6626574
ENSMUSG00000058914	C1qtnf3	9.86E-07	0.040231638
ENSMUSG00000058921	Slc10a5	2.46E-11	0.081824546
ENSMUSG00000059040	Eno1b	3.09E-18	6.659071925
ENSMUSG00000059089	Fcgr4	8.49E-22	10.04614992
ENSMUSG00000059108	Ifitm6	1.14E-17	8.9556364
ENSMUSG00000059325	Hopx	6.40E-16	0.246344501
ENSMUSG00000059326	Csf2ra	1.19E-34	6.914193096
ENSMUSG00000059463	Spag11b	0.007123395	0.051105257
ENSMUSG00000059479	B3gnt8	5.12E-09	4.045921302
ENSMUSG00000059498	Fcgr3	2.10E-35	8.366832697
ENSMUSG00000059562	Ccdc154	6.80E-07	0.246722657
ENSMUSG00000059657	Stfa2l1	5.10E-11	38.46249066
ENSMUSG00000059741	Myl3	0.000182084	0.073438038
ENSMUSG00000059854	Hydin	4.14E-10	0.182596109
ENSMUSG00000060063	Alox5ap	7.48E-15	5.287627501
ENSMUSG00000060131	Atp8b4	5.25E-38	7.963510313
ENSMUSG00000060176	Kif27	3.03E-10	0.20426975
ENSMUSG00000060183	Cxcl11	1.41E-05	1333.409081
ENSMUSG00000060212	Pcnx2	0.00067596	4.304512994
ENSMUSG00000060402	Chst8	0.000980572	0.229049237
ENSMUSG00000060445	Sycp2	3.47E-08	7.575300128
ENSMUSG00000060459	Kng2	8.16E-10	13.25224157
ENSMUSG00000060477	Irak2	3.37E-23	4.371532536
ENSMUSG00000060639	Hist1h4i	2.40E-06	4.837176693
ENSMUSG00000061080	Lsamp	0.000810875	0.023594852
ENSMUSG00000061086	Myl4	0.00334921	0.193133342
ENSMUSG00000061100	Retnla	1.63E-45	18.11961478
ENSMUSG00000061175	Fnip2	1.03E-23	7.917604182
ENSMUSG00000061397	Krt79	2.52E-09	0.233844395
ENSMUSG00000061462	Obscn	9.76E-06	0.162171164
ENSMUSG00000061540	Orm2	4.41E-24	40.71579755
ENSMUSG00000061576	Dpp6	2.52E-05	0.064993648
ENSMUSG00000061601	Pclo	3.44E-08	0.167675595
ENSMUSG00000061718	Ppp1r1b	1.44E-14	0.162034749
ENSMUSG00000061780	Cfd	1.14E-08	0.054788887
ENSMUSG00000061816	Myl1	0.004797594	0.095753092
ENSMUSG00000061959	Ces1e	4.02E-21	0.080776986
ENSMUSG00000062070	Pgk1	7.64E-14	4.01144606
ENSMUSG00000062148	Ear6	7.93E-08	35.79302375
ENSMUSG00000062184	Hs6st2	8.82E-06	0.233375519
ENSMUSG00000062257	Opcml	0.002912244	0.06717629
ENSMUSG00000062309	Rpp25	0.003741312	4.484355234
ENSMUSG00000062310	Glrp1	4.80E-05	67.80266802
ENSMUSG00000062329	Cytl1	3.15E-37	0.052090868
ENSMUSG00000062345	Serpinb2	6.54E-20	25.83424101
ENSMUSG00000062380	Tubb3	2.42E-13	12.59836285
ENSMUSG00000062382	Ftl1-ps1	3.18E-12	4.353748309

List 1 continued

ENSMUSG00000062480	Acat3	2.66E-09	12.91404594
ENSMUSG00000062563	Cys1	1.07E-13	0.123567703
ENSMUSG00000062980	Cped1	6.08E-27	0.232695126
ENSMUSG00000063130	Calml3	1.71E-12	0.052703411
ENSMUSG00000063234	Gpr84	8.96E-21	1317.76256
ENSMUSG00000063239	Grm4	0.000310641	0.134419237
ENSMUSG00000063260	Syt10	0.000171379	0.190508401
ENSMUSG00000063388	BC023105	0.001270035	18.89143822
ENSMUSG00000063428	Ddo	9.22E-12	0.247159552
ENSMUSG00000063524	Eno1	2.48E-27	6.492156825
ENSMUSG00000063529	Stmnd1	8.17E-06	0.233860755
ENSMUSG00000063623	C230062I16Rik	3.57E-06	0.150726046
ENSMUSG00000063626	Unc5d	0.000166992	0.052800718
ENSMUSG00000063632	Sox11	3.09E-17	0.127798219
ENSMUSG00000063681	Crb1	0.00155407	0.142901619
ENSMUSG00000064115	Cadm2	0.000173837	0.0955919
ENSMUSG00000064193	Gm4735	3.41E-08	4.560089437
ENSMUSG00000064225	Paqr9	0.009670562	0.105406342
ENSMUSG00000064267	Hvcn1	3.47E-30	5.447544298
ENSMUSG00000064293	Cntn4	0.00334057	0.032609768
ENSMUSG00000064294	Aox3	4.08E-22	0.177483273
ENSMUSG00000064350	mt-Ty	7.39E-07	0.188243454
ENSMUSG00000065037	Rn7sk	8.16E-07	11.71086304
ENSMUSG00000065420	Mir142b	0.000857586	8.307859565
ENSMUSG00000065987	Cd209b	0.00128219	0.080885798
ENSMUSG00000066319	Rtp3	1.22E-07	0.165579045
ENSMUSG00000066363	Serpina3f	1.22E-10	26.4246655
ENSMUSG00000066438	Plekhd1	1.12E-10	0.21699444
ENSMUSG00000066632	Pgk1-rs7	0.000195496	5.571275845
ENSMUSG00000066682	Pilrb2	3.69E-27	7.303509266
ENSMUSG00000066684	Pilrb1	1.50E-43	11.12816601
ENSMUSG00000066861	Oas1g	0.001333974	7.943615309
ENSMUSG00000067049	Unc93a	0.000682741	46.59621532
ENSMUSG00000067577	A430093F15Rik	0.003686757	4.395249052
ENSMUSG00000067642	Adgrf3	0.007797008	27.70786053
ENSMUSG00000067916	Zfp991	2.72E-12	4.722784175
ENSMUSG00000068263	Efcc1	4.13E-13	0.098555974
ENSMUSG00000068323	Slc4a5	2.11E-21	0.158951014
ENSMUSG00000068417	Pnp2	2.90E-09	5.038943197
ENSMUSG00000068522	Aard	1.34E-13	0.222569634
ENSMUSG00000068606	Gm4841	0.000223656	21.05536191
ENSMUSG00000068614	Actc1	0.0001131	0.094453962
ENSMUSG00000068745	Mybphl	0.000188177	0.123810315
ENSMUSG00000068957	4930589L23Rik	7.80E-07	179.4535101
ENSMUSG00000069170	Adgrv1	0.000621699	0.248653777
ENSMUSG00000069792	Wfdc17	4.05E-38	26.39693598
ENSMUSG00000069793	Slfn9	6.73E-05	8.494037542
ENSMUSG00000069830	Nlrp1a	1.95E-05	4.067457118
ENSMUSG00000069873	4930438A08Rik	0.000182692	23.50902031
ENSMUSG00000069910	Spdl1	4.32E-10	5.461773145
ENSMUSG00000070427	Il18bp	2.37E-20	12.37359639
ENSMUSG00000070524	Fcrlb	2.99E-37	81.89828012
ENSMUSG00000070529	Wfdc10	0.001614944	0.068427617
ENSMUSG00000070605	Zfp992	1.51E-05	4.009860003
ENSMUSG00000071230	Npw	6.83E-16	0.143401001
ENSMUSG00000071311	Gpr31b	0.002756666	12.28166985
ENSMUSG00000071324	Armc2	4.97E-05	0.245928438
ENSMUSG00000071550	Cfap44	3.62E-07	0.220245279
ENSMUSG00000071552	Tigit	6.11E-06	4.709309071
ENSMUSG00000071561	BC100530	2.76E-05	116.2072778
ENSMUSG00000071562	Stfa1	6.08E-12	74.85937808
ENSMUSG00000071604	Fam189a2	4.59E-15	0.163581583
ENSMUSG00000071713	Csf2rb	3.75E-94	14.89014276
ENSMUSG00000071714	Csf2rb2	1.23E-114	29.75729529

List 1 continued

ENSMUSG00000071715	Ncf4	9.96E-41	12.89454114
ENSMUSG00000071719	Tmem28	3.30E-07	0.107970413
ENSMUSG00000072473	1700024G13Rik	2.72E-16	0.125294526
ENSMUSG00000072572	Slc39a2	6.51E-07	4.242241353
ENSMUSG00000072601	Ear1	0.007162195	0.219583361
ENSMUSG00000072620	Slfn2	1.19E-49	9.515633288
ENSMUSG00000072621	Slfn10-ps	5.30E-22	15.29639638
ENSMUSG00000072844	G530011O06Rik	5.59E-05	18.33122749
ENSMUSG00000072949	Acot1	6.34E-11	0.153555039
ENSMUSG00000072966	Gprasp2	0.000942512	0.18828071
ENSMUSG00000073102	Drc1	7.38E-14	0.223197196
ENSMUSG00000073208	Speer4c	0.002078244	7.254267546
ENSMUSG00000073274	Gm14636	8.62E-05	19.58160788
ENSMUSG00000073293	Nudt10	0.000118187	0.122726243
ENSMUSG00000073386	9830107B12Rik	1.54E-05	5.719867464
ENSMUSG00000073400	Trim10	0.005919264	0.081780848
ENSMUSG00000073412	Lst1	5.84E-27	7.009260423
ENSMUSG00000073424	Cyp4f15	1.87E-11	0.169142906
ENSMUSG00000073489	Ifi204	1.07E-08	22.83063086
ENSMUSG00000073490	Ifi207	9.59E-18	9.171614778
ENSMUSG00000073492	Gm10521	7.06E-05	4.546629025
ENSMUSG00000073529	F830208F22Rik	1.21E-11	30.17878295
ENSMUSG00000073598	1700066B19Rik	1.92E-07	8.051008233
ENSMUSG00000073600	Prob1	2.58E-14	0.248537754
ENSMUSG00000073771	Btbd19	1.97E-07	4.003922514
ENSMUSG00000074063	Osgin1	2.75E-11	4.523556273
ENSMUSG00000074115	Saa1	1.17E-41	549.6981917
ENSMUSG00000074151	Nlrc5	0.000253384	5.611791825
ENSMUSG00000074217	2210011C24Rik	3.95E-37	0.111284589
ENSMUSG00000074342	I830077J02Rik	2.45E-11	4.24972395
ENSMUSG00000074417	Gm14548	4.15E-25	17.90386886
ENSMUSG00000074419	Gm15448	8.75E-12	6.573903188
ENSMUSG00000074505	Fat3	1.45E-07	0.209933866
ENSMUSG00000074595	Wfdc6a	0.000208071	0.152496862
ENSMUSG00000074622	Mafb	1.71E-28	5.076215718
ENSMUSG00000074673	Ttll9	0.000583106	0.202534965
ENSMUSG00000074677	Sirpb1c	1.33E-20	5.297861851
ENSMUSG00000074743	Thbd	2.96E-28	0.208622012
ENSMUSG00000074802	Gas2l3	9.62E-35	11.80216154
ENSMUSG00000074805	Il1bos	4.18E-05	6.33207195
ENSMUSG00000074813	Gm14005	5.51E-27	8.726729512
ENSMUSG00000074818	Pdzd7	1.55E-08	0.175822678
ENSMUSG00000074971	Fibin	3.86E-17	0.231757789
ENSMUSG00000074981	Dcdc5	2.09E-05	0.08519531
ENSMUSG00000075010	AW112010	1.09E-07	4.406274907
ENSMUSG00000075122	Cd80	3.72E-36	12.52559374
ENSMUSG00000075605	Slurp2	0.004126569	0.16696495
ENSMUSG00000075705	Msrb1	5.76E-30	5.790992169
ENSMUSG00000076441	Ass1	1.25E-37	7.63066292
ENSMUSG00000076614	Ighg1	7.69E-08	5.6577588
ENSMUSG00000078117	Gm16485	9.20E-05	0.063179994
ENSMUSG00000078122	F630028O10Rik	1.60E-17	8.978357261
ENSMUSG00000078137	Ankrd63	6.35E-24	0.143948768
ENSMUSG00000078144	Capns2	0.00630374	13.90251569
ENSMUSG00000078238	Gm12854	1.66E-06	4.663796837
ENSMUSG00000078252	Krtap17-1	2.98E-09	0.003470597
ENSMUSG00000078300	Gm2606	0.000358607	6.330691744
ENSMUSG00000078452	Raet1d	8.92E-11	33.40262913
ENSMUSG00000078487	Ankrd65	1.00E-07	0.180105066
ENSMUSG00000078597	Cyp4a12b	4.41E-06	0.008482238
ENSMUSG00000078607	1810010H24Rik	1.62E-18	0.153504024
ENSMUSG00000078640	Gm11627	1.01E-05	0.146715004
ENSMUSG00000078763	Slfn1	1.86E-49	13.64316537
ENSMUSG00000078783	Gm9733	2.96E-08	4.700509378

List 1 continued

ENSMUSG00000078920	Ifi47	0.003424994	5.890672666
ENSMUSG00000078922	Tgtp1	0.001275864	14.458936
ENSMUSG00000078945	Naip2	2.29E-38	11.03967405
ENSMUSG00000079012	Serpina3m	5.74E-52	21.6927426
ENSMUSG00000079014	Serpina3i	1.99E-29	23.77609017
ENSMUSG00000079022	Col22a1	0.000236506	8.789754456
ENSMUSG00000079049	Serpinb1c	0.003097793	10.36017
ENSMUSG00000079101	Esd-ps	8.65E-05	13.4391246
ENSMUSG00000079168	Cd209g	0.001454217	0.042797306
ENSMUSG00000079186	Gzmc	0.005478424	13.75764573
ENSMUSG00000079227	Ccr5	3.33E-22	10.97226013
ENSMUSG00000079293	Clec7a	1.93E-38	9.924442195
ENSMUSG00000079330	Lemd1	0.002883503	10.17836803
ENSMUSG00000079343	C1s2	0.001455604	45.13768938
ENSMUSG00000079355	Ackr4	9.15E-11	0.117509765
ENSMUSG00000079419	Ms4a6c	1.24E-29	7.779985392
ENSMUSG00000079434	Neu2	9.20E-05	0.168224147
ENSMUSG00000079436	Kcnj13	0.000100583	0.212977408
ENSMUSG00000079584	Gm364	0.002185792	55.17889033
ENSMUSG00000079597	Gm5483	4.22E-08	59.89311278
ENSMUSG00000079625	Tm4sf19	1.37E-14	169.750389
ENSMUSG00000079700	Fpr3	4.67E-09	276.7631472
ENSMUSG00000079722	Ttll2	0.009152523	42.59688843
ENSMUSG00000080242	Atp6v0c-ps2	1.09E-05	5.456412084
ENSMUSG00000080783	Gm8250	0.000493256	6.74718138
ENSMUSG00000081058	Hist2h3c2	0.00153078	0.218501492
ENSMUSG00000081650	Gm16181	2.56E-08	12.2105179
ENSMUSG00000081664	Gm15544	0.00085286	42.97431612
ENSMUSG00000081665	Gm15922	1.13E-18	13.44593032
ENSMUSG00000081723	Gm15931	3.71E-13	8.2025798
ENSMUSG00000082016	Pgam1-ps2	1.36E-05	5.97655042
ENSMUSG00000082292	Gm12250	0.002501599	10.44464094
ENSMUSG00000082419	Gm11425	1.51E-07	11.65716111
ENSMUSG00000082976	Gm15056	1.55E-07	189.4794036
ENSMUSG00000083041	Gmfg-ps	0.004695709	7.746805579
ENSMUSG00000083282	Ctsf	7.27E-17	0.188570404
ENSMUSG00000083773	Gm13394	4.82E-05	5.153988133
ENSMUSG00000083849	Gm13477	0.008888343	5.105522032
ENSMUSG00000084350	Znf41-ps	2.69E-10	4.829294726
ENSMUSG00000084497	Gm22107	7.28E-05	10.3792951
ENSMUSG00000084796	Mir142hg	1.40E-09	4.604759676
ENSMUSG00000084902	Gm281	2.32E-12	0.133838281
ENSMUSG00000084918	Gm12708	0.001929845	6.991106528
ENSMUSG00000084939	Gm830	1.11E-05	0.116819194
ENSMUSG00000084941	Gm11944	4.56E-05	0.135166656
ENSMUSG00000084960	B430010I23Rik	1.00E-10	0.19977518
ENSMUSG00000084989	Crocc2	1.12E-06	0.175726833
ENSMUSG00000085069	Gm13111	2.45E-19	0.129015555
ENSMUSG00000085124	Gm12766	0.006096452	0.177197285
ENSMUSG00000085126	Gm12589	0.000191873	13.31878152
ENSMUSG00000085156	Snhg15	1.18E-09	4.274747617
ENSMUSG00000085295	4930430E12Rik	1.23E-38	548.5327233
ENSMUSG00000085298	F730035M05Rik	0.001484579	64.71459971
ENSMUSG00000085407	1700095J03Rik	0.001204862	0.110848706
ENSMUSG00000085498	Gm14023	2.70E-08	8.602860772
ENSMUSG00000085514	Bcas3os2	0.002600423	0.175218953
ENSMUSG00000085517	Gm12963	0.002246649	0.24723563
ENSMUSG00000085531	Slc36a3os	5.12E-09	12.21348864
ENSMUSG00000085558	4930412C18Rik	1.74E-05	0.222413071
ENSMUSG00000085682	Gm14267	0.001828369	52.72039313
ENSMUSG00000085894	Gm15832	1.16E-24	11.44279243
ENSMUSG00000085903	Gm15340	5.23E-05	102.2507404
ENSMUSG00000085915	Gm8091	4.57E-05	14.62560707
ENSMUSG00000085939	Cd63-ps	2.88E-18	4.574897268

List 1 continued

ENSMUSG00000085976	Gm13816	6.31E-05	0.205264684
ENSMUSG00000085977	Gm5970	1.59E-06	23.51914619
ENSMUSG00000086109	Gm13391	1.18E-07	28.6894943
ENSMUSG00000086141	9030622O22Rik	1.61E-05	0.219465786
ENSMUSG00000086150	Bach2os	0.001200853	12.54206267
ENSMUSG00000086196	Gm13571	2.56E-11	472.6143822
ENSMUSG00000086335	Gm12107	0.00017558	35.47179668
ENSMUSG00000086499	Gm16217	0.003371975	47.89603938
ENSMUSG00000086727	4931428L18Rik	0.003119085	28.72117344
ENSMUSG00000086755	Gm11216	0.00013093	82.0423011
ENSMUSG00000086822	5330413P13Rik	0.000375892	0.240149523
ENSMUSG00000086843	E030013I19Rik	2.45E-11	0.104169103
ENSMUSG00000086868	Gm15883	7.18E-09	0.076885287
ENSMUSG00000086952	Gm12596	0.000370736	49.71802183
ENSMUSG00000087001	Gm15475	9.62E-07	0.108877137
ENSMUSG00000087113	Gm11714	0.000982036	18.7765416
ENSMUSG00000087263	Gm15726	0.002500398	18.51734404
ENSMUSG00000087477	Gm13822	5.62E-08	10.91006539
ENSMUSG00000087624	9230111E07Rik	0.000127355	6.101609676
ENSMUSG00000089712	Gm15889	3.78E-06	0.201364131
ENSMUSG00000089844	A530032D15Rik	0.004160137	5.452292384
ENSMUSG00000089874	9230117E06Rik	0.000127413	0.140465861
ENSMUSG00000089929	Bcl2a1b	1.43E-65	15.44641615
ENSMUSG00000089931	Gm8459	5.94E-05	45.48857817
ENSMUSG00000089942	Pira2	1.69E-24	13.87308801
ENSMUSG00000089999	Gm6485	9.31E-06	4.871133323
ENSMUSG00000090124	Ugt1a7c	7.07E-18	10.11909104
ENSMUSG00000090173	Fbxw10	0.000275683	4.179919357
ENSMUSG00000090192	Gm16556	0.006541546	4.548780015
ENSMUSG00000090230	Gm16315	7.90E-05	19.60547242
ENSMUSG00000090231	Cfb	3.31E-26	11.91708958
ENSMUSG00000090257	Gm4524	7.48E-06	0.064748871
ENSMUSG00000090291	Lrrc10b	2.41E-08	0.242937661
ENSMUSG00000090307	1700071M16Rik	7.96E-16	21.45135786
ENSMUSG00000090379	Gm8229	8.62E-05	0.011717731
ENSMUSG00000090439	Gm17455	0.002574262	10.78402393
ENSMUSG00000090564	A430057M04Rik	0.000202728	14.08212978
ENSMUSG00000090576	Gm17055	5.68E-05	5.290380468
ENSMUSG00000090799	Klhl33	0.001713993	0.225753473
ENSMUSG00000091230	Gm6970	0.00383077	0.197239733
ENSMUSG00000091415	Ak9	9.00E-07	0.233892704
ENSMUSG00000091618	H60c	2.42E-05	0.047169304
ENSMUSG00000091712	Sec14l5	1.40E-06	0.165734366
ENSMUSG00000091730	Gm17230	5.42E-05	27.14642907
ENSMUSG00000092171	4833427F10Rik	0.001979743	7.276220243
ENSMUSG00000092418	Gm20406	1.06E-06	38.77691142
ENSMUSG00000092517	Art2a-ps	0.001917759	5.736122692
ENSMUSG00000093384	Gm20689	0.005771566	4.468862951
ENSMUSG00000093622	Gm20703	0.003573618	10.5812763
ENSMUSG00000093765	Gm20658	5.03E-05	12.60076779
ENSMUSG00000093954	Gm16867	6.27E-11	7.135988751
ENSMUSG00000093973	Mrgpra2a	2.57E-09	316.9026597
ENSMUSG00000094504	Gm5294	0.005614743	41.95897298
ENSMUSG00000094626	Tmem121b	1.29E-07	0.137979958
ENSMUSG00000094707	A830019P07Rik	0.005748778	0.024778665
ENSMUSG00000094733	Gm5416	6.61E-08	34.33204922
ENSMUSG00000095028	Sirpb1b	1.59E-16	6.244355992
ENSMUSG00000095061	E030018B13Rik	0.002186476	13.34282383
ENSMUSG00000095134	Mid1-ps1	0.000524448	81.61759122
ENSMUSG00000095493	A630023A22Rik	0.000511563	62.73934801
ENSMUSG00000095576	Fmo6	0.001616288	0.074696879
ENSMUSG00000095609	Gm21188	1.73E-28	7.777957921
ENSMUSG00000095620	2010005H15Rik	5.07E-08	32.62064874
ENSMUSG00000095788	Sirpb1a	2.91E-14	4.193884437

List 1 continued

ENSMUSG00000096141	Dnah7a	3.94E-06	0.237861
ENSMUSG00000096617	Gm5559	6.73E-11	4.261875954
ENSMUSG00000096719	Mrgpra2b	3.64E-21	71.0739483
ENSMUSG00000096768	Gm47283	0.00260737	6.008347669
ENSMUSG00000096960	A230028O05Rik	0.002948083	47.77617509
ENSMUSG00000096965	3300005D01Rik	0.000581305	15.46044498
ENSMUSG00000097077	Gm16712	0.001625938	12.70626949
ENSMUSG00000097134	1110002J07Rik	4.21E-09	20.68132814
ENSMUSG00000097139	Gm26626	5.26E-09	63.84590307
ENSMUSG00000097178	2310002F09Rik	0.001126351	0.061105524
ENSMUSG00000097194	9330175E14Rik	1.67E-07	6.981831924
ENSMUSG00000097203	4732419C18Rik	0.003317685	16.0150919
ENSMUSG00000097236	4831440E17Rik	0.006834146	0.240438093
ENSMUSG00000097352	C920009B18Rik	7.21E-20	10.16799986
ENSMUSG00000097418	Mir155hg	6.60E-09	13.35425038
ENSMUSG00000097453	Gm26894	0.001238872	0.201477614
ENSMUSG00000097462	9530026P05Rik	1.37E-13	0.162563161
ENSMUSG00000097471	5830432E09Rik	1.82E-10	5.936973764
ENSMUSG00000097504	4930516B21Rik	9.20E-09	0.132944482
ENSMUSG00000097610	A930012L18Rik	0.00721307	0.185389918
ENSMUSG00000097636	Mirt1	1.34E-15	9.8701423
ENSMUSG00000097726	9530036O11Rik	9.71E-07	0.161149957
ENSMUSG00000097740	E030044B06Rik	0.000202779	0.029282125
ENSMUSG00000097742	Gm26535	0.002767146	22.67046801
ENSMUSG00000097756	A730056A06Rik	0.002719011	0.021726338
ENSMUSG00000097779	4833407H14Rik	8.08E-21	10.30260379
ENSMUSG00000097789	Gm2115	0.000176756	0.187462041
ENSMUSG00000097804	Gm16685	4.07E-07	6.862854025
ENSMUSG00000097815	Gm26809	4.00E-23	8.004658169
ENSMUSG00000097848	Gm807	6.53E-05	105.2897167
ENSMUSG00000097855	A930007I19Rik	5.98E-33	16.49601792
ENSMUSG00000097857	Gm26603	0.008139119	26.8916091
ENSMUSG00000097877	Gm26703	1.87E-06	9.294906527
ENSMUSG00000098008	A930001A20Rik	0.000194542	0.029722809
ENSMUSG00000098320	Vis1	5.22E-06	69.79895145
ENSMUSG00000098434	2010110E17Rik	6.51E-05	88.59237521
ENSMUSG00000098470	C1rb	3.69E-06	44.96950974
ENSMUSG00000098489	Mir7678	0.001954058	54.86050179
ENSMUSG00000098708	Gm27252	5.14E-07	8.035063475
ENSMUSG00000098975	Gm27177	0.004020222	10.14969453
ENSMUSG00000099338	2810030D12Rik	1.05E-05	0.139203281
ENSMUSG00000099398	Ms4a14	0.000108732	7.647552335
ENSMUSG00000099875	Rbm3-ps	8.15E-07	4.175088346
ENSMUSG00000099930	Gm2396	0.000202779	73.37749391
ENSMUSG00000099974	Bcl2a1d	5.50E-46	9.011009195
ENSMUSG00000100916	Lhb	8.54E-07	0.192491223
ENSMUSG00000101132	Gm8000	0.000941498	7.976839234
ENSMUSG00000101389	Ms4a4a	1.26E-40	14.53837182
ENSMUSG00000101517	4732465J04Rik	0.000500498	7.055369074
ENSMUSG00000101872	Gm29237	1.20E-12	0.201333482
ENSMUSG00000102037	Bcl2a1a	7.09E-51	8.633521819
ENSMUSG00000102051	I830127L07Rik	3.18E-11	6.070089765
ENSMUSG00000102155	Gm37468	0.002008584	6.605083235
ENSMUSG00000102269	Gm7357	0.002619754	0.075018065
ENSMUSG00000102329	Gm10851	0.001940707	4.231480529
ENSMUSG00000102376	Gm37975	0.003887586	8.265303041
ENSMUSG00000102471	Gm5100	0.009956172	28.24304715
ENSMUSG00000102697	Pcdhac2	3.00E-20	0.126641353
ENSMUSG00000102715	Gm6209	2.72E-07	49.10330275
ENSMUSG00000102895	5830415G21Rik	6.12E-05	65.70619656
ENSMUSG00000102975	Gm37347	1.54E-05	22.38589511
ENSMUSG00000103174	Gm37168	0.000343961	10.11788219
ENSMUSG00000103219	Gm37787	0.009838802	29.08686824
ENSMUSG00000103308	Gm37800	4.40E-08	197.6365217

List 1 continued

ENSMUSG00000103439	Gm7019	3.75E-05	6.612248983
ENSMUSG00000103588	Gm18445	7.06E-05	90.8913337
ENSMUSG00000103847	Gm20056	6.35E-16	39.34230897
ENSMUSG00000103907	Gm37498	8.43E-05	81.54086115
ENSMUSG00000104068	Gm37199	4.22E-07	76.32251892
ENSMUSG00000104082	Gm7115	3.59E-07	24.87588501
ENSMUSG00000104348	Gm37691	6.54E-35	22.34310772
ENSMUSG00000104467	Gm37660	0.000823019	7.848466973
ENSMUSG00000104728	Gm42462	1.51E-29	76.54867461
ENSMUSG00000104818	Gm43661	2.17E-05	37.50054616
ENSMUSG00000104913	Gm6560	5.13E-15	5.711852292
ENSMUSG00000105006	Gm9484	0.006204832	5.271758813
ENSMUSG00000105112	Gm42778	0.000390374	0.217649401
ENSMUSG00000105357	Gm42647	1.71E-06	81.14386275
ENSMUSG00000105403	Gm43618	0.004560294	0.093925704
ENSMUSG00000105504	Gbp5	4.62E-06	18.73126225
ENSMUSG00000105746	Gm43595	6.66E-05	0.133105722
ENSMUSG00000105769	Gm43351	0.009192096	37.44848192
ENSMUSG00000105843	Gm19439	1.76E-15	0.150298144
ENSMUSG00000105987	AI506816	8.77E-23	4.760221375
ENSMUSG00000106261	Gm34086	0.00766714	42.24301127
ENSMUSG00000106416	Gm5857	0.002316463	0.204083813
ENSMUSG00000106478	Gm36551	7.11E-07	11.61700907
ENSMUSG00000106574	Gm2451	0.002950186	6.62881783
ENSMUSG00000106609	Gm43181	2.04E-56	101.8270521
ENSMUSG00000106755	Tpi-rs11	2.77E-05	4.785286037
ENSMUSG00000106783	Chaer1	0.000114818	4.574608366
ENSMUSG00000106874	Gm20186	1.69E-06	4.674923049
ENSMUSG00000107111	Gm40304	0.004145518	44.06129911
ENSMUSG00000107191	Gm43579	0.004392962	0.023834437
ENSMUSG00000107317	Gm19719	0.00902013	4.730552284
ENSMUSG00000107355	AI839979	4.86E-11	7.302115628
ENSMUSG00000107451	Gm44421	0.000172806	0.171661212
ENSMUSG00000107479	2610300M13Rik	0.001454217	24.76517545
ENSMUSG00000107480	Gm44165	0.006357958	11.62317443
ENSMUSG00000107655	Gm44220	6.10E-18	0.086944473
ENSMUSG00000107768	Gm44275	0.000116018	17.56496825
ENSMUSG00000107770	Gm44126	0.000189569	6.213595919
ENSMUSG00000107811	Gm44000	0.00459056	0.235104017
ENSMUSG00000108010	Gm38708	0.000232663	0.190006271
ENSMUSG00000108024	Gm43912	0.004513175	5.886405232
ENSMUSG00000108059	Gm44369	0.004189305	10.81501677
ENSMUSG00000108161	Gm32914	0.000515901	22.61028395
ENSMUSG00000108210	Gm35808	7.62E-06	95.08709297
ENSMUSG00000108255	Gm16499	0.001017089	0.024471037
ENSMUSG00000108291	Gm44292	7.90E-05	63.9359637
ENSMUSG00000108358	Gm44509	3.81E-07	0.232591156
ENSMUSG00000108436	Gm44851	3.63E-06	20.32541619
ENSMUSG00000108481	Gm33248	2.85E-06	0.219701401
ENSMUSG00000108580	Gm39094	0.000337969	0.094560303
ENSMUSG00000108695	Gm2511	4.20E-08	248.9724355
ENSMUSG00000108859	Gm44776	0.009945294	39.29759266
ENSMUSG00000108950	9130015G15Rik	1.76E-08	0.217414832
ENSMUSG00000109005	Gm45221	5.66E-07	11.91331913
ENSMUSG00000109036	2210406H18Rik	1.48E-09	7.068978247
ENSMUSG00000109251	E230032D23Rik	5.70E-13	19.1549092
ENSMUSG00000109321	A330076H08Rik	0.003634256	0.199667722
ENSMUSG00000109587	Gm31105	7.04E-06	0.184798608
ENSMUSG00000109669	Gm45472	0.000466354	10.77543936
ENSMUSG00000109674	Gm45470	0.000221765	0.104704547
ENSMUSG00000109864	Eid3	0.003887586	6.827533911
ENSMUSG00000110185	Igip	3.50E-07	0.234647039
ENSMUSG00000110344	Gm45716	7.75E-06	4.037968492
ENSMUSG00000110380	Gm45332	9.58E-05	0.222167917

List 1 continued

ENSMUSG00000110397	Gm45540	2.87E-06	0.112018733
ENSMUSG00000110427	4933406B17Rik	0.000277297	0.153530717
ENSMUSG00000110498	A630001O12Rik	4.29E-06	7.867610278
ENSMUSG00000110588	Gm45774	0.000882027	59.86335351
ENSMUSG00000110697	Gm31718	2.46E-36	40.91622053
ENSMUSG00000110702	Gm45767	0.001550687	0.246849975
ENSMUSG00000110891	Gm40639	0.002065419	0.021029608
ENSMUSG00000111116	Gm48065	7.25E-06	22.15340529
ENSMUSG00000111394	AC160637.1	7.14E-13	4.792393934
ENSMUSG00000111662	Gm7435	0.000514285	0.124934816
ENSMUSG00000111713	Gm20234	0.001381142	4.646487724
ENSMUSG00000111720	Gm39459	0.000167263	80.84905369
ENSMUSG00000111752	Gm38575	8.67E-05	88.97720506
ENSMUSG00000112023	Lilr4b	6.99E-60	42.84896477
ENSMUSG00000112129	Pbld1	0.000732331	0.093369879
ENSMUSG00000112146	Gm46210	2.68E-05	14.21939805
ENSMUSG00000112148	Lilrb4a	3.47E-33	21.817734
ENSMUSG00000112226	Gm48786	0.000375892	17.16503474
ENSMUSG00000112265	Gm34983	0.002271315	8.151104604
ENSMUSG00000112500	4933411E08Rik	0.009869343	0.059921626
ENSMUSG00000112618	Gm48835	0.003881582	0.114174237
ENSMUSG00000112758	Gm36172	0.000828723	16.30117915
ENSMUSG00000112762	4930459C07Rik	1.54E-05	81.66365131
ENSMUSG00000112766	Gm40645	0.009815075	18.0141194
ENSMUSG00000112792	Gm38407	4.31E-11	9.053008209
ENSMUSG00000112843	Gm46224	7.08E-14	30.45384327
ENSMUSG00000112980	D430020J02Rik	2.43E-11	6.224902811
ENSMUSG00000113136	Gm19951	5.56E-20	15.88595291
ENSMUSG00000113309	Gm48408	0.002345329	50.30839063
ENSMUSG00000113338	A530046M15Rik	0.000346258	76.56484411
ENSMUSG00000113346	Eprn	8.12E-06	5.000528858
ENSMUSG00000113553	Gm40932	0.000111277	23.85792046
ENSMUSG00000113701	B230303A05Rik	1.12E-16	255.7173117
ENSMUSG00000113960	4933412O06Rik	2.43E-08	213.7681453
ENSMUSG00000114033	Gm47705	0.002733306	5.264415568
ENSMUSG00000114055	Gm32089	2.79E-09	297.2277071
ENSMUSG00000114068	Gm48783	0.000300281	28.41973429
ENSMUSG00000114161	Gm48662	2.94E-09	0.105566749
ENSMUSG00000114245	Gm38655	2.26E-09	0.128446341
ENSMUSG00000114278	Gm49027	0.000404467	0.142575444
ENSMUSG00000114355	Gm40916	0.001560183	40.56136772
ENSMUSG00000114414	A930014D07Rik	0.000454236	64.38243983
ENSMUSG00000114608	Gm36161	8.21E-20	8.447685353
ENSMUSG00000114662	Gm31683	0.003094656	6.666721892
ENSMUSG00000114755	Galr3	0.000988157	11.29764155
ENSMUSG00000114784	Gm47754	0.009069915	6.395323872
ENSMUSG00000114980	4933432I03Rik	8.98E-07	120.7961408
ENSMUSG00000114988	Gm49226	0.002474396	50.61914968
ENSMUSG00000115026	Gm49041	0.00062898	4.660909612
ENSMUSG00000115205	Gm16374	0.007343971	8.751093443
ENSMUSG00000115355	4930445E18Rik	0.001007284	61.54606017
ENSMUSG00000115391	Gm49123	0.00221147	50.55502406
ENSMUSG00000115426	Gm19510	4.46E-10	323.1002369
ENSMUSG00000115439	Gm36107	0.001395752	56.02761034
ENSMUSG00000115480	Gm49249	0.003706695	12.51613556
ENSMUSG00000115518	Gm10791	0.000578304	4.963699787
ENSMUSG00000115536	Gm32857	0.000518619	0.034663798
ENSMUSG00000115593	Gm4824	0.009716917	4.670646765
ENSMUSG00000115801	AC160336.1	2.15E-05	0.070795983
ENSMUSG00000115816	Gm34589	0.006709293	5.944562303
ENSMUSG00000115855	Gm34643	1.68E-10	374.0622207
ENSMUSG00000115869	Gm31814	2.08E-14	19.13057757
ENSMUSG00000115902	AC113595.1	3.12E-15	4.940427384
ENSMUSG00000115919	Gm31583	0.00239599	32.66660997

List 1 continued

ENSMUSG00000115946	Mirt2	6.79E-07	24.81581919
ENSMUSG00000116380	Gm39556	2.38E-06	4.054982703
ENSMUSG00000116657	AC127341.3	7.65E-07	13.58494023
ENSMUSG00000116720	AC154546.2	0.004529363	0.247150304
ENSMUSG00000116935	AC117241.2	0.000121043	82.47455111
ENSMUSG00000117232	CT030665.2	0.000115386	11.77089159
ENSMUSG00000117309	CT010583.2	0.000276478	12.33494844
ENSMUSG00000117315	AC154218.2	0.000610067	28.30403511
ENSMUSG00000117318	AC241601.2	0.000225845	16.10299974

List 2. Differentially Expressed Genes between Diabetic+DMOG, A. f vs Diabetic, A. f

gene_id	gene_name	padj (Diabetic+DMOG vs Diabetic)	Fold-Change (Diabetic+DMOG vs Diabetic)
ENSMUSG00000000031	H19	1.32E-05	11.01150343
ENSMUSG00000000204	Slfn4	0.004742932	0.193554861
ENSMUSG00000000982	Ccl3	6.83E-07	0.109129139
ENSMUSG00000000983	Wfdc18	0.006818173	0.161590382
ENSMUSG00000001131	Timp1	6.55E-18	0.208331927
ENSMUSG00000001156	Mxd1	1.51E-26	0.219501448
ENSMUSG00000001865	Cpa3	0.000374254	4.085676331
ENSMUSG00000002588	Pon1	8.59E-21	4.88196508
ENSMUSG00000003477	Inmt	2.73E-21	6.761238303
ENSMUSG00000004035	Gstm7	1.63E-07	4.633950739
ENSMUSG00000004371	Il11	2.89E-05	0.186995547
ENSMUSG00000004814	Ccl24	7.33E-06	0.212934934
ENSMUSG00000005054	Cstb	6.44E-14	0.237706494
ENSMUSG00000005057	Sh2b2	1.85E-12	0.198686397
ENSMUSG00000005320	Fgfr4	1.92E-07	4.065607136
ENSMUSG00000005547	Cyp2a5	2.10E-09	4.851691725
ENSMUSG00000005800	Mmp8	0.00477947	0.187180721
ENSMUSG00000006235	Epor	1.85E-06	4.167555338
ENSMUSG00000006269	Atp6v1b1	1.30E-09	23.47181641
ENSMUSG00000006403	Adamts4	3.71E-12	0.160703746
ENSMUSG00000006567	Atp7b	1.45E-15	4.925891867
ENSMUSG00000006724	Cyp27b1	0.001422969	0.086972505
ENSMUSG00000007279	Scube2	3.63E-27	5.608852036
ENSMUSG00000007877	Tcap	3.35E-05	7.463814307
ENSMUSG00000007946	Phox2a	1.67E-05	0.146686051
ENSMUSG00000010651	Acaa1b	0.000169863	4.073623774
ENSMUSG00000013483	Card14	0.004667878	4.812785282
ENSMUSG00000014542	Clec4f	0.006748923	7.370537018
ENSMUSG00000014782	Plekhg4	0.000202482	0.148470804
ENSMUSG00000015354	Pcolce2	2.77E-16	4.948629167
ENSMUSG00000015970	Chdh	0.000230577	4.865780861
ENSMUSG00000016349	Eef1a2	0.001517209	4.791931302
ENSMUSG00000016496	Cd274	4.02E-14	0.148503956
ENSMUSG00000016529	Il10	9.73E-06	0.105488266
ENSMUSG00000017002	Slpi	7.56E-05	0.161741835
ENSMUSG00000018566	Slc2a4	3.92E-06	4.339252065
ENSMUSG00000018893	Mb	6.93E-08	15.51386395
ENSMUSG00000018930	Ccl4	4.73E-26	0.059414395
ENSMUSG00000019787	Trdn	0.007884042	6.093114748
ENSMUSG00000019850	Tnfaip3	2.49E-17	0.213885176
ENSMUSG00000019880	Rspo3	8.55E-10	0.165476396
ENSMUSG00000019905	Gprc6a	1.08E-08	4.718291091
ENSMUSG00000020022	Ndufa12	1.91E-28	0.207289708
ENSMUSG00000020096	Tbata	0.000383542	6.898569231
ENSMUSG00000020182	Ddc	1.03E-07	5.492045089
ENSMUSG00000020218	Wif1	2.38E-05	5.619550546
ENSMUSG00000020227	Irak3	7.17E-10	0.243590802
ENSMUSG00000020251	Glt8d2	1.02E-08	5.375513883
ENSMUSG00000020334	Slc22a4	6.62E-07	0.220625056
ENSMUSG00000020407	Upp1	8.67E-43	0.178490508
ENSMUSG00000020431	Adcy1	4.77E-05	11.4013578
ENSMUSG00000020524	Gria1	2.30E-08	4.836321568
ENSMUSG00000020591	Ntsr2	0.000450995	6.235955469
ENSMUSG00000020620	Abca8b	1.09E-10	4.029208739
ENSMUSG00000020641	Rsad2	0.002926344	0.097917937
ENSMUSG00000020826	Nos2	5.29E-26	0.06849956
ENSMUSG00000020990	Cdkl1	4.66E-06	4.242820768
ENSMUSG00000021055	Esr2	2.73E-05	4.019367421
ENSMUSG00000021198	Unc79	3.45E-06	6.293882747
ENSMUSG00000021278	Amn	0.008587908	0.12536013
ENSMUSG00000021281	Tnfaip2	1.83E-25	0.231980835
ENSMUSG00000021322	Aoah	6.36E-37	0.230843126
ENSMUSG00000021591	Glrx	2.39E-12	0.242473012
ENSMUSG00000021750	Fam107a	8.95E-11	4.701504844
ENSMUSG00000022026	Olfm4	0.00013823	0.167040911
ENSMUSG00000022126	Acod1	8.36E-41	0.103658522
ENSMUSG00000022206	Npr3	1.38E-09	4.12674082
ENSMUSG00000022262	Dnah5	4.97E-09	4.671790504
ENSMUSG00000022304	Dpys	0.001077072	0.188888367
ENSMUSG00000022367	Has2	0.002703001	0.184819854
ENSMUSG00000022534	Mefv	4.08E-22	0.215845775

List 2 continued

ENSMUSG00000022586	Ly6i	5.16E-14	0.188039247
ENSMUSG00000022758	P2rx6	7.17E-13	6.076360748
ENSMUSG00000022759	Lrrc74b	6.40E-10	4.584088678
ENSMUSG00000023011	Faim2	1.17E-14	6.599861403
ENSMUSG00000023349	Clec4n	1.27E-14	0.217633918
ENSMUSG00000023913	Pla2g7	2.32E-26	0.225761091
ENSMUSG00000024008	Cpne5	0.007401267	4.202342644
ENSMUSG00000024041	Cryaa	0.007225085	4.892408062
ENSMUSG00000024401	Tnf	3.58E-27	0.081748552
ENSMUSG00000024679	Ms4a6d	1.01E-19	0.217530949
ENSMUSG00000024737	Slc15a3	3.67E-32	0.227624141
ENSMUSG00000024774	Ankrd22	0.00398197	0.24565208
ENSMUSG00000024798	Htr7	3.94E-11	0.146762809
ENSMUSG00000024953	Prdx5	4.91E-51	0.192938074
ENSMUSG00000025044	Msr1	2.57E-20	0.215709157
ENSMUSG00000025165	Sectm1a	0.009218806	0.244787935
ENSMUSG00000025318	Jph3	0.000121111	0.198799364
ENSMUSG00000025383	Il23a	4.65E-11	0.144711817
ENSMUSG00000025479	Cyp2e1	8.11E-05	6.538174913
ENSMUSG00000025746	Il6	1.08E-05	0.082454048
ENSMUSG00000025789	St8sia2	2.06E-06	4.439622575
ENSMUSG00000025854	Fam20c	4.43E-12	0.22996691
ENSMUSG00000025877	Hk3	2.82E-24	0.203895381
ENSMUSG00000025936	Gm4956	5.44E-08	4.664401332
ENSMUSG00000026018	Ica1l	0.003003138	6.157413918
ENSMUSG00000026073	Il1r2	2.96E-11	0.190130736
ENSMUSG00000026177	Slc11a1	2.63E-13	0.232333606
ENSMUSG00000026304	Rab17	0.000132648	4.727534131
ENSMUSG00000026358	Rgs1	2.79E-08	0.203612789
ENSMUSG00000026398	Nr5a2	9.72E-07	0.228636534
ENSMUSG00000026475	Rgs16	7.30E-18	0.180110538
ENSMUSG00000026536	Ifi211	0.000545332	0.15157194
ENSMUSG00000026580	Selp	1.18E-20	0.214382646
ENSMUSG00000026582	Sele	1.13E-13	0.112159079
ENSMUSG00000026676	Ccdc3	0.009630676	11.13416121
ENSMUSG00000026691	Fmo3	3.47E-08	5.262275123
ENSMUSG00000026768	Itga8	3.58E-27	5.878007999
ENSMUSG00000026981	Il1rn	2.87E-15	0.174795811
ENSMUSG00000026984	Il1f6	0.000363903	0.013949402
ENSMUSG00000027335	Adra1d	0.001069303	4.091072596
ENSMUSG00000027398	Il1b	1.67E-24	0.117804928
ENSMUSG00000027399	Il1a	1.39E-24	0.090170443
ENSMUSG00000027442	Cst8	5.20E-05	11.04608557
ENSMUSG00000027513	Pck1	0.008895282	11.34351509
ENSMUSG00000027514	Zbp1	0.007905012	0.205911488
ENSMUSG00000027559	Car3	3.24E-05	8.707795397
ENSMUSG00000027560	Dok5	4.80E-05	0.08832037
ENSMUSG00000027611	Procr	2.90E-20	0.205637375
ENSMUSG00000027737	Slc7a11	1.53E-09	0.211425806
ENSMUSG00000027776	Il12a	1.19E-13	0.152218752
ENSMUSG00000027832	Ptx3	6.75E-06	0.082483511
ENSMUSG00000028270	Gbp2	0.003526202	0.152023138
ENSMUSG00000028356	Ambp	0.000251341	0.115395623
ENSMUSG00000028364	Tnc	1.39E-11	0.234171885
ENSMUSG00000028488	Sh3gl2	1.58E-05	6.001900743
ENSMUSG00000028599	Tnfrsf1b	1.65E-24	0.236599915
ENSMUSG00000028602	Tnfrsf8	1.52E-10	0.202344864
ENSMUSG00000028730	Cfap57	8.48E-14	4.846697159
ENSMUSG00000028940	Hes2	0.000601265	9.811923482
ENSMUSG00000028996	Rbp7	0.00172218	6.458536177
ENSMUSG00000029082	Bst1	2.24E-35	0.227576107
ENSMUSG00000029273	Sult1d1	1.22E-06	6.149668205
ENSMUSG00000029371	Cxcl5	2.00E-16	0.135063534
ENSMUSG00000029377	Ereg	2.63E-06	0.158908027
ENSMUSG00000029379	Cxcl3	9.13E-09	0.209041893
ENSMUSG00000029380	Cxcl1	2.36E-15	0.095824333
ENSMUSG00000029417	Cxcl9	0.00626627	0.111104922
ENSMUSG00000029778	Adcyap1r1	9.25E-05	4.237212232
ENSMUSG00000029819	Npy	0.006477309	0.0918083
ENSMUSG00000029843	Slc13a4	7.07E-05	4.68235823
ENSMUSG00000029915	Clec5a	5.46E-27	0.165471778
ENSMUSG00000030048	Gkn3	0.000816303	10.82836953

List 2 continued

ENSMUSG00000030069	Prok2	1.41E-08	0.094407961
ENSMUSG00000030087	Klf15	1.47E-36	5.231376662
ENSMUSG00000030108	Slc6a13	1.06E-06	0.243890895
ENSMUSG00000030125	Lrrc23	1.28E-08	4.431117824
ENSMUSG00000030142	Clec4e	1.56E-24	0.130964363
ENSMUSG00000030144	Clec4d	6.85E-13	0.155089729
ENSMUSG00000030268	Bcat1	0.009265841	0.246337743
ENSMUSG00000030278	Cidec	8.82E-07	5.113456719
ENSMUSG00000030302	Atp2b2	7.42E-08	6.319952654
ENSMUSG00000030399	Ckm	0.002862428	34.21278097
ENSMUSG00000030402	Ppm1n	4.66E-06	0.099717723
ENSMUSG00000030470	Csrp3	1.11E-11	13.61880722
ENSMUSG00000030495	Slc7a10	2.74E-08	4.9324912
ENSMUSG00000030546	Plin1	0.005754904	9.015547941
ENSMUSG00000030562	Nox4	5.35E-10	4.111975125
ENSMUSG00000030737	Slco2b1	3.33E-17	4.610214771
ENSMUSG00000031104	Rab33a	0.007330699	0.193299119
ENSMUSG00000031163	Glod5	0.002749641	0.084854603
ENSMUSG00000031257	Nox1	0.000117928	7.721584712
ENSMUSG00000031294	D630029K05Rik	0.001652566	4.446368812
ENSMUSG00000031444	F10	7.96E-27	0.140170875
ENSMUSG00000031461	Myom2	7.33E-08	11.64152042
ENSMUSG00000031554	Adam5	0.003774595	11.78908173
ENSMUSG00000031725	Ces1f	4.95E-08	18.17853204
ENSMUSG00000031870	Pgr	2.60E-05	4.051384332
ENSMUSG00000031881	Cdh16	1.07E-12	4.199310776
ENSMUSG00000031966	Glb1l3	0.002653691	5.424934709
ENSMUSG00000032315	Cyp1a1	3.29E-28	13.5133289
ENSMUSG00000032487	Ptgs2	8.06E-10	0.22992373
ENSMUSG00000032532	Cck	0.004632294	0.049469097
ENSMUSG00000032661	Oas3	0.004412565	0.205495833
ENSMUSG00000032691	Nlrp3	4.61E-16	0.203978559
ENSMUSG00000032925	Itgbl1	0.000107974	5.022455484
ENSMUSG00000032942	Ucp3	2.90E-06	7.291944174
ENSMUSG00000033207	Mamdc2	1.06E-18	5.851643921
ENSMUSG00000033213	AA467197	4.92E-34	0.133402737
ENSMUSG00000033508	Asprv1	0.001830273	0.129917584
ENSMUSG00000033644	Piwil2	2.03E-07	0.219115488
ENSMUSG00000033967	Rnf225	5.58E-07	4.338662821
ENSMUSG00000034226	Rhov	2.76E-12	0.179372626
ENSMUSG00000034391	Fbxo15	0.00191754	0.184446458
ENSMUSG00000034416	Pkd1l2	0.0070866	0.222402271
ENSMUSG00000034855	Cxcl10	2.95E-05	0.050853551
ENSMUSG00000035186	Ubd	1.27E-05	0.053814305
ENSMUSG00000035208	Slfn8	0.009290678	0.23864066
ENSMUSG00000035296	Sgcg	2.46E-05	7.444996392
ENSMUSG00000035352	Ccl12	2.46E-08	0.221482926
ENSMUSG00000035373	Ccl7	1.19E-14	0.1001819
ENSMUSG00000035385	Ccl2	5.44E-35	0.109975304
ENSMUSG00000035407	Kank4	1.27E-10	4.132778603
ENSMUSG00000035448	Ccr3	0.001203483	4.194931294
ENSMUSG00000035459	Stab2	0.000172288	4.099943994
ENSMUSG00000035486	Plk5	0.001513051	6.805192283
ENSMUSG00000035686	Thrsp	3.40E-06	5.344900633
ENSMUSG00000035692	Isg15	0.002672999	0.119481795
ENSMUSG00000035910	Dcdc2a	4.77E-08	6.99745827
ENSMUSG00000036095	Dgkb	0.00366343	6.924009533
ENSMUSG00000036931	Nfkbid	1.30E-11	0.218189854
ENSMUSG00000037477	Tbx10	0.00562314	0.02647108
ENSMUSG00000037492	Zmat4	6.65E-06	11.59976159
ENSMUSG00000037613	Tnfrsf23	2.27E-10	0.233335761
ENSMUSG00000037771	Slc32a1	0.004926946	0.181148722
ENSMUSG00000037872	Ackr1	9.90E-05	0.195025572
ENSMUSG00000037966	Ninj1	9.37E-18	0.206922385
ENSMUSG00000037973	Ccdc129	7.37E-05	4.332825264
ENSMUSG00000038037	Socs1	0.001058261	0.154410871
ENSMUSG00000038067	Csf3	4.45E-06	0.061818728
ENSMUSG00000038209	Itln1	0.005346167	10.87637124
ENSMUSG00000038259	Gdf5	0.00244691	8.580409667
ENSMUSG00000038357	Camp	0.000331597	0.242043067
ENSMUSG00000038756	Ttll6	2.81E-07	6.358491421
ENSMUSG00000038791	Scgb3a2	9.22E-15	4.705821326

List 2 continued

ENSMUSG00000039084	Chad	2.14E-08	4.474972991
ENSMUSG00000039196	Orm1	1.43E-25	0.224216958
ENSMUSG00000039257	Vstm2b	0.00219286	11.9378852
ENSMUSG00000039395	Mreg	2.55E-13	0.17115876
ENSMUSG00000039883	Lrrc17	6.59E-05	4.211412399
ENSMUSG00000040026	Saa3	3.92E-22	0.172660624
ENSMUSG00000040752	Myh6	4.75E-05	11.22829958
ENSMUSG00000040838	Gm11639	8.14E-06	0.048298537
ENSMUSG00000040950	Mgl2	8.95E-20	4.329061996
ENSMUSG00000041324	Inhba	1.17E-23	0.20114505
ENSMUSG00000041380	Htr2c	0.000683774	8.461613935
ENSMUSG00000041423	Paqr6	8.35E-07	4.838055437
ENSMUSG00000041624	Gucy1a2	6.01E-09	4.392926062
ENSMUSG00000041644	Slc5a12	0.000887734	5.113274032
ENSMUSG00000041789	2700046A07Rik	0.00113364	10.68282698
ENSMUSG00000041827	Oasl1	0.008794306	0.160914479
ENSMUSG00000041828	Abca8a	1.22E-08	5.502449697
ENSMUSG00000042109	Csdc2	6.15E-06	5.638014188
ENSMUSG00000042254	Cilp	6.00E-05	8.175059727
ENSMUSG00000042265	Trem1	1.22E-09	0.193619862
ENSMUSG00000042349	Ikbke	8.49E-31	0.215876167
ENSMUSG00000042529	Kcnj12	0.000171362	4.155822795
ENSMUSG00000042793	Lgr6	8.29E-07	5.808785189
ENSMUSG00000043333	Rhbdl2	0.001071886	0.219876023
ENSMUSG00000043424	Eif3j2	0.000132473	0.161344144
ENSMUSG00000043496	Tril	1.52E-18	4.56183954
ENSMUSG00000043631	Ecm2	8.78E-09	4.554204118
ENSMUSG00000043953	Ccrl2	2.72E-36	0.18513749
ENSMUSG00000044103	Il1f9	3.02E-05	0.153107071
ENSMUSG00000044156	Hepacam2	0.000539943	4.02448736
ENSMUSG00000044352	Sowaha	0.000837182	5.199540532
ENSMUSG00000044365	Cxxc4	0.000275655	5.309090454
ENSMUSG00000044701	Il27	4.35E-06	0.131962006
ENSMUSG00000044903	Psg22	0.004566425	0.108123474
ENSMUSG00000044951	Mylk4	0.004549182	8.655175509
ENSMUSG00000045027	Prss22	0.000346826	0.205009629
ENSMUSG00000045362	Tnfrsf26	1.57E-13	0.219845486
ENSMUSG00000045381	Olfr433	0.00237161	0.116784929
ENSMUSG00000045502	Hcar2	3.13E-22	0.161589607
ENSMUSG00000045534	Kcna5	0.000308851	11.54926983
ENSMUSG00000045551	Fpr1	2.02E-11	0.16264979
ENSMUSG00000045875	Adra1a	6.17E-17	4.055325929
ENSMUSG00000045915	Ccdc42	0.000434986	4.845972974
ENSMUSG00000046093	Hpcal4	0.000614651	8.465944773
ENSMUSG00000046182	Gsg1l	1.08E-05	14.90927748
ENSMUSG00000046196	Ttc39d	0.009439233	37.23829252
ENSMUSG00000046610	Oacyl	2.59E-06	0.211335435
ENSMUSG00000046618	Olfml2a	5.61E-12	4.768951546
ENSMUSG00000046828	Mettl21e	5.15E-06	5.724778556
ENSMUSG00000047021	Cfap65	5.59E-10	4.564098226
ENSMUSG00000047443	Erfe	0.000186342	0.185781701
ENSMUSG00000047841	Fndc11	0.005587613	7.038431782
ENSMUSG00000047945	Marcksl1	4.59E-38	0.146296664
ENSMUSG00000048038	Ccdc187	3.21E-15	4.384338451
ENSMUSG00000048621	Gm6377	1.16E-09	0.205444998
ENSMUSG00000048752	Prss50	0.002992253	0.153580059
ENSMUSG00000048834	Vstm2a	0.00065559	5.710001072
ENSMUSG00000049511	Htr1b	0.001262302	4.482375327
ENSMUSG00000050089	Akap4	9.28E-09	0.059285019
ENSMUSG00000050097	Ces2b	0.000427592	5.094247704
ENSMUSG00000050359	Sprr1a	0.000688194	0.214671204
ENSMUSG00000050395	Tnfsf15	8.30E-16	0.180601007
ENSMUSG00000050578	Mmp13	3.51E-10	0.228894864
ENSMUSG00000051439	Cd14	1.65E-05	0.143146627
ENSMUSG00000051747	Ttn	1.42E-10	5.176206679
ENSMUSG00000051748	Wfdc21	0.000290852	0.195746578
ENSMUSG00000051906	Cd209f	0.000113136	10.05158203
ENSMUSG00000052270	Fpr2	1.55E-20	0.160780849
ENSMUSG00000052974	Cyp2f2	5.69E-15	6.554004493
ENSMUSG00000053161	Daw1	0.005152125	4.344707612
ENSMUSG00000053318	Slamf8	0.000981936	0.154935977
ENSMUSG00000053338	Tarm1	3.85E-22	0.12474645

List 2 continued

ENSMUSG00000053475	Tnfaip6	1.85E-06	0.181123703
ENSMUSG00000053647	Gper1	0.000177658	4.596918178
ENSMUSG00000054146	Krt15	0.000715217	24.79606911
ENSMUSG00000054203	Ifi205	9.15E-05	0.106056353
ENSMUSG00000054422	Fabp1	0.000710842	8.697051196
ENSMUSG00000054619	Mettl7a1	3.39E-12	4.267468213
ENSMUSG00000054855	Rnd1	1.95E-21	0.173703314
ENSMUSG00000054905	Stfa3	0.000292118	0.072935242
ENSMUSG00000055170	Ifng	0.003114146	0.205154598
ENSMUSG00000055368	Slc6a2	4.07E-13	4.897330345
ENSMUSG00000056054	S100a8	8.75E-05	0.132725377
ENSMUSG00000056071	S100a9	0.002521649	0.202527474
ENSMUSG00000056529	Ptafr	1.33E-28	0.191338705
ENSMUSG00000057182	Scn3a	2.26E-19	6.740149577
ENSMUSG00000057465	Saa2	3.45E-06	0.159881304
ENSMUSG00000057606	Colq	1.25E-17	11.79056778
ENSMUSG00000057933	Gsta2	0.001715172	5.198246595
ENSMUSG00000058159	T2	0.005473137	8.250821183
ENSMUSG00000058260	Serpina9	0.002288302	4.058470665
ENSMUSG00000058427	Cxcl2	0.0001281	0.083349823
ENSMUSG00000058755	Osm	3.61E-11	0.146757167
ENSMUSG00000058914	C1qtnf3	0.005661761	11.04337
ENSMUSG00000058921	Slc10a5	0.00067873	4.585211859
ENSMUSG00000059854	Hydin	2.40E-09	4.633299772
ENSMUSG00000060183	Cxcl11	0.00131531	0.045257974
ENSMUSG00000061397	Krt79	9.55E-13	4.889820187
ENSMUSG00000061462	Obscn	3.47E-05	5.322267972
ENSMUSG00000061576	Dpp6	0.003895059	7.661661738
ENSMUSG00000061718	Ppp1r1b	6.17E-13	4.256544195
ENSMUSG00000061742	Slc22a12	0.003583415	4.675731894
ENSMUSG00000061780	Cfd	0.002698981	7.374977259
ENSMUSG00000061959	Ces1e	1.93E-06	5.052550084
ENSMUSG00000062329	Cytl1	4.43E-12	7.084536641
ENSMUSG00000062345	Serpinb2	6.03E-12	0.107729598
ENSMUSG00000062480	Acat3	0.000114596	0.229970513
ENSMUSG00000062760	Shisal1	1.76E-08	4.890261725
ENSMUSG00000063130	Calml3	0.001901396	5.037381352
ENSMUSG00000063234	Gpr84	2.46E-21	0.098857983
ENSMUSG00000063626	Unc5d	0.00344372	11.70289282
ENSMUSG00000065037	Rn7sk	0.000109069	0.101064076
ENSMUSG00000066687	Zbtb16	3.81E-17	4.129664576
ENSMUSG00000068263	Efcc1	1.76E-05	5.168420199
ENSMUSG00000068373	D430041D05Rik	1.20E-06	6.924504101
ENSMUSG00000068463	B630019A10Rik	0.00662603	4.941943913
ENSMUSG00000068614	Actc1	2.36E-08	6.213508853
ENSMUSG00000069792	Wfdc17	6.71E-14	0.177469251
ENSMUSG00000069873	4930438A08Rik	0.000132369	0.060401117
ENSMUSG00000070427	Il18bp	2.98E-07	0.225274285
ENSMUSG00000070524	Fcrlb	5.21E-10	0.207435298
ENSMUSG00000071317	Bves	0.002668971	7.28746452
ENSMUSG00000072473	1700024G13Rik	1.70E-07	4.581904071
ENSMUSG00000072620	Slfn2	1.51E-26	0.242232475
ENSMUSG00000072844	G530011O06Rik	0.004129984	0.134481634
ENSMUSG00000073274	Gm14636	0.004488388	0.248577927
ENSMUSG00000073489	Ifi204	0.001338531	0.153930897
ENSMUSG00000074115	Saa1	0.003050162	0.206878911
ENSMUSG00000074505	Fat3	2.44E-05	4.009153839
ENSMUSG00000074595	Wfdc6a	0.00129491	5.248116795
ENSMUSG00000074651	Mcidas	0.001200887	5.150019602
ENSMUSG00000074981	Dcdc5	0.000348263	7.591777267
ENSMUSG00000076550	Igkv4-63	0.001908242	23.51296937
ENSMUSG00000078137	Ankrd63	1.30E-17	4.752787723
ENSMUSG00000078252	Krtap17-1	0.003698087	41.18885152
ENSMUSG00000079101	Esd-ps	0.006546568	0.229466222
ENSMUSG00000079597	Gm5483	0.004320481	0.159324331
ENSMUSG00000079700	Fpr3	1.03E-08	0.050711158
ENSMUSG00000081650	Gm16181	3.59E-08	0.135000288
ENSMUSG00000081664	Gm15544	0.004295616	0.125778817
ENSMUSG00000082976	Gm15056	6.30E-06	0.06420482
ENSMUSG00000084902	Gm281	6.30E-05	4.411921832
ENSMUSG00000084941	Gm11944	0.000721415	5.185919659
ENSMUSG00000084989	Crocc2	7.17E-08	8.648852964

List 2 continued

ENSMUSG00000085295	4930430E12Rik	4.81E-23	0.171021368
ENSMUSG00000085407	1700095J03Rik	0.00178392	7.982074758
ENSMUSG00000085498	Gm14023	6.37E-08	0.153589273
ENSMUSG00000086755	Gm11216	0.00462387	0.08536235
ENSMUSG00000086843	E030013I19Rik	6.72E-07	6.574963686
ENSMUSG00000087477	Gm13822	3.22E-07	0.150808062
ENSMUSG00000089874	9230117E06Rik	0.000334833	5.894619773
ENSMUSG00000090007	Rpl30-ps2	0.001627693	47.18812667
ENSMUSG00000090230	Gm16315	9.81E-05	0.074344961
ENSMUSG00000090257	Gm4524	0.003876678	7.563568983
ENSMUSG00000091890	A830073O21Rik	0.00540973	5.576875144
ENSMUSG00000092418	Gm20406	0.002628028	0.16385993
ENSMUSG00000093973	Mrgpra2a	2.47E-05	0.117469056
ENSMUSG00000094198	Ighv1-50	0.003262453	0.12594681
ENSMUSG00000094733	Gm5416	0.001370557	0.149498758
ENSMUSG00000095620	2010005H15Rik	0.00010004	0.077265274
ENSMUSG00000096719	Mrgpra2b	3.55E-07	0.115818536
ENSMUSG00000097139	Gm26626	0.000225763	0.229560203
ENSMUSG00000097418	Mir155hg	9.05E-06	0.189199065
ENSMUSG00000097453	Gm26894	0.006387096	4.076955691
ENSMUSG00000097504	4930516B21Rik	8.05E-05	4.245778144
ENSMUSG00000098008	A930001A20Rik	0.000901738	25.77830895
ENSMUSG00000101693	Gm19461	1.12E-05	4.183217896
ENSMUSG00000102269	Gm7357	0.000215932	15.32338183
ENSMUSG00000102697	Pcdhac2	1.09E-08	4.380746328
ENSMUSG00000103308	Gm37800	0.002001732	0.240363453
ENSMUSG00000103588	Gm18445	6.21E-05	0.010661965
ENSMUSG00000104621	Gm43185	0.004304435	41.7663041
ENSMUSG00000104728	Gm42462	1.16E-09	0.229445849
ENSMUSG00000104818	Gm43661	0.005996952	0.182007835
ENSMUSG00000105112	Gm42778	2.02E-05	4.909549148
ENSMUSG00000105286	Gm43140	0.004305391	37.76947582
ENSMUSG00000105357	Gm42647	8.39E-06	0.083694861
ENSMUSG00000105504	Gbp5	0.003314677	0.125942953
ENSMUSG00000106609	Gm43181	3.99E-17	0.168237453
ENSMUSG00000107191	Gm43579	0.009086609	34.60228674
ENSMUSG00000107655	Gm44220	7.50E-11	5.245017552
ENSMUSG00000108010	Gm38708	8.86E-06	5.30647441
ENSMUSG00000108161	Gm32914	0.003140755	0.129779221
ENSMUSG00000108210	Gm35808	0.001124329	0.209049191
ENSMUSG00000108255	Gm16499	0.004047884	28.04658308
ENSMUSG00000108291	Gm44292	0.004153633	0.224436734
ENSMUSG00000108691	Gm45226	0.007497045	35.84781607
ENSMUSG00000108695	Gm2511	0.002544051	0.231985273
ENSMUSG00000108950	9130015G15Rik	3.42E-17	5.612155047
ENSMUSG00000109251	E230032D23Rik	2.21E-06	0.178924674
ENSMUSG00000109674	Gm45470	0.003513881	6.600834956
ENSMUSG00000110266	Gm32742	0.001409311	6.084971847
ENSMUSG00000110397	Gm45540	3.41E-08	8.452724111
ENSMUSG00000112023	Lilr4b	3.69E-17	0.243339792
ENSMUSG00000112129	Pbld1	0.001069303	7.002536749
ENSMUSG00000112146	Gm46210	3.06E-05	0.091378979
ENSMUSG00000112762	4930459C07Rik	0.000114755	0.1323854
ENSMUSG00000113106	Gm47139	0.000568715	9.079746237
ENSMUSG00000113701	B230303A05Rik	8.46E-15	0.118919241
ENSMUSG00000113960	4933412O06Rik	2.88E-07	0.059065617
ENSMUSG00000114161	Gm48662	0.00048608	4.747302045
ENSMUSG00000114245	Gm38655	1.52E-05	5.19517975
ENSMUSG00000115153	Gm49240	0.003654285	47.7848164
ENSMUSG00000115919	Gm31583	0.004281737	0.184554852
ENSMUSG00000115946	Mirt2	0.006047899	0.19581
ENSMUSG00000116657	AC127341.3	0.001953441	0.241970495
ENSMUSG00000117318	AC241601.2	0.004219055	0.1831127

List 3. Differentially Expressed Genes that are common between Nondiabetic, A. f vs Diabetic, A. f and between Diabetic+DMOG, A. f vs Diabetic, A. f

gene_id	gene_name	padj (diabetic vs nondiabetic)	Fold-Change (diabetic vs nondiabetic)	padj (DMOG vs vehicle)	Fold-Change (DMOG vs vehicle)
ENSMUSG00000000031	H19	0.009985938	0.166051202	1.32E-05	11.01150343
ENSMUSG00000000204	Slfn4	1.30E-11	43.93092057	0.004742932	0.193554861
ENSMUSG00000000982	Ccl3	4.29E-31	265.3431412	6.83E-07	0.109129139
ENSMUSG00000000983	Wfdc18	3.26E-05	98.1432878	0.006818173	0.161590382
ENSMUSG00000001131	Timp1	7.10E-191	81.05246061	6.55E-18	0.208331927
ENSMUSG00000001156	Mxd1	1.21E-62	10.66813084	1.51E-26	0.219501448
ENSMUSG00000001865	Cpa3	1.38E-05	0.194535534	0.000374254	4.085676331
ENSMUSG00000002588	Pon1	2.06E-68	0.060837085	8.59E-21	4.88196508
ENSMUSG00000003477	Inmt	3.55E-25	0.101976957	2.73E-21	6.761238303
ENSMUSG00000004035	Gstm7	8.48E-06	0.249433326	1.63E-07	4.633950739
ENSMUSG00000004371	Il11	6.16E-05	5.439592966	2.89E-05	0.186995547
ENSMUSG00000004814	Ccl24	4.61E-09	7.520270475	7.33E-06	0.212934934
ENSMUSG00000005054	Cstb	3.13E-41	12.00865313	6.44E-14	0.237706494
ENSMUSG00000005057	Sh2b2	1.11E-44	27.24464566	1.85E-12	0.198686397
ENSMUSG00000005320	Fgfr4	8.73E-11	0.154631943	1.92E-07	4.065607136
ENSMUSG00000005547	Cyp2a5	2.18E-08	0.067534238	2.10E-09	4.851691725
ENSMUSG00000005800	Mmp8	4.20E-08	35.91504923	0.00477947	0.187180721
ENSMUSG00000006269	Atp6v1b1	3.13E-14	0.028232776	1.30E-09	23.47181641
ENSMUSG00000006403	Adamts4	3.38E-69	65.687058	3.71E-12	0.160703746
ENSMUSG00000006567	Atp7b	3.30E-10	0.246868054	1.45E-15	4.925891867
ENSMUSG00000006724	Cyp27b1	0.000816855	18.41560312	0.001422969	0.086972505
ENSMUSG00000007279	Scube2	1.92E-30	0.119835292	3.63E-27	5.608852036
ENSMUSG00000007946	Phox2a	1.34E-08	117.5994875	1.67E-05	0.146686051
ENSMUSG00000010651	Acaa1b	6.47E-16	0.084690655	0.000169863	4.073623774
ENSMUSG00000014782	Plekhg4	8.67E-05	8.257345956	0.000202482	0.148470804
ENSMUSG00000015354	Pcolce2	3.01E-32	0.089416776	2.77E-16	4.948629167
ENSMUSG00000016349	Eef1a2	1.35E-06	0.119978207	0.001517209	4.791931302
ENSMUSG00000016496	Cd274	5.61E-39	22.42449759	4.02E-14	0.148503956
ENSMUSG00000016529	Il10	3.91E-08	129.1821376	9.73E-06	0.105488266
ENSMUSG00000017002	Slpi	1.12E-29	19.92518516	7.56E-05	0.161741835
ENSMUSG00000018566	Slc2a4	0.001453651	0.24170566	3.92E-06	4.339252065
ENSMUSG00000018893	Mb	3.28E-06	0.015254189	6.93E-08	15.51386395
ENSMUSG00000018930	Ccl4	5.00E-96	230.4394182	4.73E-26	0.059414395
ENSMUSG00000019850	Tnfaip3	1.05E-63	15.09667011	2.49E-17	0.213885176
ENSMUSG00000019905	Gprc6a	4.08E-05	0.242027467	1.08E-08	4.718291091
ENSMUSG00000020096	Tbata	8.81E-07	0.076197917	0.000383542	6.898569231
ENSMUSG00000020182	Ddc	2.07E-07	0.173299449	1.03E-07	5.492045089
ENSMUSG00000020218	Wif1	5.98E-09	0.11821602	2.38E-05	5.619550546
ENSMUSG00000020227	Irak3	5.06E-29	10.07713264	7.17E-10	0.243590802
ENSMUSG00000020251	Glt8d2	3.12E-13	0.106555363	1.02E-08	5.375513883
ENSMUSG00000020334	Slc22a4	8.65E-23	24.49985099	6.62E-07	0.220625056
ENSMUSG00000020407	Upp1	8.26E-27	8.082492153	8.67E-43	0.178490508
ENSMUSG00000020431	Adcy1	5.61E-10	0.04346502	4.77E-05	11.4013578

List 3 continued

ENSMUSG00000020524	Gria1	4.46E-09	0.169055063	2.30E-08	4.836321568
ENSMUSG00000020620	Abca8b	1.77E-13	0.192922147	1.09E-10	4.029208739
ENSMUSG00000020641	Rsad2	1.21E-06	32.12861469	0.002926344	0.097917937
ENSMUSG00000020826	Nos2	6.25E-68	61.91897814	5.29E-26	0.06849956
ENSMUSG00000020990	Cdkl1	1.75E-05	0.204908478	4.66E-06	4.242820768
ENSMUSG00000021055	Esr2	6.34E-07	0.179905474	2.73E-05	4.019367421
ENSMUSG00000021198	Unc79	2.16E-06	0.130085172	3.45E-06	6.293882747
ENSMUSG00000021278	Amn	0.003383318	15.4400543	0.008587908	0.12536013
ENSMUSG00000021281	Tnfaip2	1.05E-79	13.25759319	1.83E-25	0.231980835
ENSMUSG00000021322	Aoah	6.94E-48	26.02086521	6.36E-37	0.230843126
ENSMUSG00000021591	Glrx	3.15E-23	7.042394205	2.39E-12	0.242473012
ENSMUSG00000022026	Olfm4	1.33E-05	28.67562233	0.00013823	0.167040911
ENSMUSG00000022126	Acod1	1.21E-229	1697.405047	8.36E-41	0.103658522
ENSMUSG00000022206	Npr3	9.04E-19	0.160647997	1.38E-09	4.12674082
ENSMUSG00000022262	Dnah5	5.10E-09	0.198980719	4.97E-09	4.671790504
ENSMUSG00000022304	Dpys	6.37E-05	20.33597147	0.001077072	0.188888367
ENSMUSG00000022367	Has2	6.60E-06	13.89308569	0.002703001	0.184819854
ENSMUSG00000022534	Mefv	3.64E-37	33.73451435	4.08E-22	0.215845775
ENSMUSG00000022586	Ly6i	1.83E-99	123.3372728	5.16E-14	0.188039247
ENSMUSG00000022758	P2rx6	6.02E-14	0.136562152	7.17E-13	6.076360748
ENSMUSG00000022759	Lrrc74b	1.80E-09	0.18044745	6.40E-10	4.584088678
ENSMUSG00000023011	Faim2	1.11E-19	0.102925282	1.17E-14	6.599861403
ENSMUSG00000023349	Clec4n	4.50E-56	33.75549839	1.27E-14	0.217633918
ENSMUSG00000023913	Pla2g7	1.07E-68	29.46701967	2.32E-26	0.225761091
ENSMUSG00000024008	Cpne5	1.88E-12	0.088383877	0.007401267	4.202342644
ENSMUSG00000024041	Cryaa	0.001272025	0.161369426	0.007225085	4.892408062
ENSMUSG00000024401	Tnf	7.43E-128	368.3201252	3.58E-27	0.081748552
ENSMUSG00000024679	Ms4a6d	3.93E-43	18.99537541	1.01E-19	0.217530949
ENSMUSG00000024737	Slc15a3	5.98E-80	12.92728355	3.67E-32	0.227624141
ENSMUSG00000024774	Ankrd22	1.10E-06	14.57313389	0.00398197	0.24565208
ENSMUSG00000024798	Htr7	7.88E-26	20.21591029	3.94E-11	0.146762809
ENSMUSG00000024953	Prdx5	5.63E-63	8.541310834	4.91E-51	0.192938074
ENSMUSG00000025044	Msr1	1.51E-115	65.15012469	2.57E-20	0.215709157
ENSMUSG00000025165	Sectm1a	1.67E-06	20.86993407	0.009218806	0.244787935
ENSMUSG00000025318	Jph3	3.26E-06	13.7285589	0.000121111	0.198799364
ENSMUSG00000025383	Il23a	1.19E-16	50.78814098	4.65E-11	0.144711817
ENSMUSG00000025479	Cyp2e1	7.82E-05	0.056338338	8.11E-05	6.538174913
ENSMUSG00000025746	Il6	4.75E-13	128.5313558	1.08E-05	0.082454048
ENSMUSG00000025789	St8sia2	3.41E-09	0.171713603	2.06E-06	4.439622575
ENSMUSG00000025854	Fam20c	3.01E-27	9.143303908	4.43E-12	0.22996691
ENSMUSG00000025877	Hk3	5.24E-99	37.003006	2.82E-24	0.203895381
ENSMUSG00000025936	Gm4956	9.24E-13	0.136203951	5.44E-08	4.664401332
ENSMUSG00000026073	Il1r2	1.27E-69	95.9813252	2.96E-11	0.190130736
ENSMUSG00000026177	Slc11a1	3.33E-82	36.07569496	2.63E-13	0.232333606

List 3 continued

ENSMUSG00000026358	Rgs1	1.55E-31	21.41536675	2.79E-08	0.203612789
ENSMUSG00000026475	Rgs16	2.87E-21	10.98402608	7.30E-18	0.180110538
ENSMUSG00000026536	Ifi211	1.67E-07	17.85386194	0.000545332	0.15157194
ENSMUSG00000026580	Selp	2.28E-35	11.34401679	1.18E-20	0.214382646
ENSMUSG00000026582	Sele	3.91E-16	9.750339672	1.13E-13	0.112159079
ENSMUSG00000026676	Ccdc3	0.000798567	0.056221337	0.009630676	11.13416121
ENSMUSG00000026691	Fmo3	3.20E-18	0.080384481	3.47E-08	5.262275123
ENSMUSG00000026768	Itga8	5.19E-25	0.166650067	3.58E-27	5.878007999
ENSMUSG00000026981	Il1rn	6.18E-180	100.7363338	2.87E-15	0.174795811
ENSMUSG00000026984	Il1f6	2.17E-05	135.1112892	0.000363903	0.013949402
ENSMUSG00000027398	Il1b	7.90E-59	46.71511426	1.67E-24	0.117804928
ENSMUSG00000027399	Il1a	1.06E-100	95.66406742	1.39E-24	0.090170443
ENSMUSG00000027442	Cst8	6.85E-18	0.023804885	5.20E-05	11.04608557
ENSMUSG00000027513	Pck1	1.86E-05	0.052631554	0.008895282	11.34351509
ENSMUSG00000027514	Zbp1	7.62E-06	14.29554446	0.007905012	0.205911488
ENSMUSG00000027559	Car3	9.46E-23	0.037233943	3.24E-05	8.707795397
ENSMUSG00000027560	Dok5	6.17E-05	25.63412077	4.80E-05	0.08832037
ENSMUSG00000027611	Procr	1.68E-44	13.332059	2.90E-20	0.205637375
ENSMUSG00000027737	Slc7a11	3.30E-134	267.9960415	1.53E-09	0.211425806
ENSMUSG00000027776	Il12a	7.67E-13	6.606409415	1.19E-13	0.152218752
ENSMUSG00000027832	Ptx3	4.69E-07	38.25712855	6.75E-06	0.082483511
ENSMUSG00000028270	Gbp2	0.000106504	12.11334918	0.003526202	0.152023138
ENSMUSG00000028356	Ambp	1.11E-05	21.64944276	0.000251341	0.115395623
ENSMUSG00000028364	Tnc	6.46E-85	14.4160296	1.39E-11	0.234171885
ENSMUSG00000028488	Sh3gl2	4.30E-11	0.089501607	1.58E-05	6.001900743
ENSMUSG00000028599	Tnfrsf1b	2.43E-80	16.38198225	1.65E-24	0.236599915
ENSMUSG00000028602	Tnfrsf8	1.20E-27	22.61613691	1.52E-10	0.202344864
ENSMUSG00000028730	Cfap57	6.24E-14	0.158895753	8.48E-14	4.846697159
ENSMUSG00000028940	Hes2	1.37E-14	0.01987271	0.000601265	9.811923482
ENSMUSG00000028996	Rbp7	0.000353119	0.116509519	0.00172218	6.458536177
ENSMUSG00000029082	Bst1	1.08E-63	10.95796274	2.24E-35	0.227576107
ENSMUSG00000029273	Sult1d1	3.29E-05	0.06749397	1.22E-06	6.149668205
ENSMUSG00000029371	Cxcl5	0.00027586	25.58258012	2.00E-16	0.135063534
ENSMUSG00000029377	Ereg	5.90E-15	29.18604353	2.63E-06	0.158908027
ENSMUSG00000029379	Cxcl3	1.08E-24	1088.523538	9.13E-09	0.209041893
ENSMUSG00000029380	Cxcl1	8.74E-62	118.7823175	2.36E-15	0.095824333
ENSMUSG00000029417	Cxcl9	5.53E-08	99.62982301	0.00626627	0.111104922
ENSMUSG00000029819	Npy	0.000201484	76.13465475	0.006477309	0.0918083
ENSMUSG00000029843	Slc13a4	8.28E-09	0.129408724	7.07E-05	4.68235823
ENSMUSG00000029915	Clec5a	3.50E-141	62.39076324	5.46E-27	0.165471778
ENSMUSG00000030048	Gkn3	6.80E-06	0.052958704	0.000816303	10.82836953
ENSMUSG00000030069	Prok2	7.08E-11	454.7258137	1.41E-08	0.094407961
ENSMUSG00000030087	Klf15	1.07E-19	0.205281152	1.47E-36	5.231376662
ENSMUSG00000030108	Slc6a13	2.69E-11	99.7393417	1.06E-06	0.243890895

List 3 continued

ENSMUSG00000030125	Lrrc23	4.45E-13	0.212705449	1.28E-08	4.431117824
ENSMUSG00000030142	Clec4e	2.24E-128	241.3105668	1.56E-24	0.130964363
ENSMUSG00000030144	Clec4d	4.92E-75	96.47048929	6.85E-13	0.155089729
ENSMUSG00000030268	Bcat1	0.000133207	15.03273861	0.009265841	0.246337743
ENSMUSG00000030278	Cidec	1.78E-18	0.069135367	8.82E-07	5.113456719
ENSMUSG00000030302	Atp2b2	2.47E-13	0.107286349	7.42E-08	6.319952654
ENSMUSG00000030399	Ckm	6.86E-06	0.009655962	0.002862428	34.21278097
ENSMUSG00000030402	Ppm1n	1.15E-06	23.13707331	4.66E-06	0.099717723
ENSMUSG00000030470	Csrp3	5.33E-12	0.051141131	1.11E-11	13.61880722
ENSMUSG00000030495	Slc7a10	5.12E-15	0.116617817	2.74E-08	4.9324912
ENSMUSG00000030546	Plin1	2.81E-05	0.070892409	0.005754904	9.015547941
ENSMUSG00000030737	Slco2b1	3.10E-08	0.249515092	3.33E-17	4.610214771
ENSMUSG00000031104	Rab33a	0.000617015	11.73471111	0.007330699	0.193299119
ENSMUSG00000031163	Glod5	0.000572794	19.14692167	0.002749641	0.084854603
ENSMUSG00000031294	D630029K05Rik	8.61E-05	0.154713002	0.001652566	4.446368812
ENSMUSG00000031444	F10	8.34E-95	51.78242703	7.96E-27	0.140170875
ENSMUSG00000031461	Myom2	3.98E-05	0.104289638	7.33E-08	11.64152042
ENSMUSG00000031554	Adam5	0.000137313	0.058030505	0.003774595	11.78908173
ENSMUSG00000031725	Ces1f	6.29E-21	0.014239424	4.95E-08	18.17853204
ENSMUSG00000031870	Pgr	2.28E-11	0.095675478	2.60E-05	4.051384332
ENSMUSG00000031881	Cdh16	1.31E-10	0.201810819	1.07E-12	4.199310776
ENSMUSG00000031966	Glb1l3	6.54E-05	0.119032399	0.002653691	5.424934709
ENSMUSG00000032315	Cyp1a1	0.002869363	0.080778694	3.29E-28	13.5133289
ENSMUSG00000032487	Ptgs2	5.53E-12	6.263341608	8.06E-10	0.22992373
ENSMUSG00000032532	Cck	0.000244202	71.39567193	0.004632294	0.049469097
ENSMUSG00000032661	Oas3	1.89E-08	16.74428331	0.004412565	0.205495833
ENSMUSG00000032691	Nlrp3	4.94E-63	30.45240139	4.61E-16	0.203978559
ENSMUSG00000032925	Itgbl1	7.54E-10	0.105203708	0.000107974	5.022455484
ENSMUSG00000032942	Ucp3	4.92E-08	0.108652559	2.90E-06	7.291944174
ENSMUSG00000033207	Mamdc2	6.95E-19	0.158833594	1.06E-18	5.851643921
ENSMUSG00000033213	AA467197	3.39E-206	164.3650773	4.92E-34	0.133402737
ENSMUSG00000033508	Asprv1	2.12E-17	140.8372685	0.001830273	0.129917584
ENSMUSG00000033644	Piwil2	8.17E-29	32.1708933	2.03E-07	0.219115488
ENSMUSG00000034226	Rhov	4.68E-32	18.09076045	2.76E-12	0.179372626
ENSMUSG00000034391	Fbxo15	0.000730854	5.85726711	0.00191754	0.184446458
ENSMUSG00000034416	Pkd1l2	7.41E-06	15.17779501	0.0070866	0.222402271
ENSMUSG00000034855	Cxcl10	5.60E-11	178.795138	2.95E-05	0.050853551
ENSMUSG00000035186	Ubd	1.76E-11	150.9277132	1.27E-05	0.053814305
ENSMUSG00000035208	Slfn8	4.75E-05	8.353439853	0.009290678	0.23864066
ENSMUSG00000035296	Sgcg	2.85E-05	0.116100079	2.46E-05	7.444996392
ENSMUSG00000035352	Ccl12	4.38E-30	22.2673239	2.46E-08	0.221482926
ENSMUSG00000035373	Ccl7	5.09E-14	84.49006544	1.19E-14	0.1001819
ENSMUSG00000035385	Ccl2	9.08E-109	202.4628751	5.44E-35	0.109975304
ENSMUSG00000035407	Kank4	2.43E-25	0.10803296	1.27E-10	4.132778603

List 3 continued

ENSMUSG00000035486	Plk5	1.11E-06	0.086158371	0.001513051	6.805192283
ENSMUSG00000035686	Thrsp	8.85E-12	0.07027376	3.40E-06	5.344900633
ENSMUSG00000035692	Isg15	0.001002914	11.70461549	0.002672999	0.119481795
ENSMUSG00000035910	Dcdc2a	1.01E-13	0.073129947	4.77E-08	6.99745827
ENSMUSG00000036095	Dgkb	0.000569245	0.123814598	0.00366343	6.924009533
ENSMUSG00000036931	Nfkbid	1.87E-41	14.39227709	1.30E-11	0.218189854
ENSMUSG00000037477	Tbx10	0.00096279	71.21764626	0.00562314	0.02647108
ENSMUSG00000037492	Zmat4	0.000305685	0.109756143	6.65E-06	11.59976159
ENSMUSG00000037613	Tnfrsf23	3.93E-44	23.59447379	2.27E-10	0.233335761
ENSMUSG00000037771	Slc32a1	0.000332876	12.50054815	0.004926946	0.181148722
ENSMUSG00000037872	Ackr1	1.79E-09	21.34661068	9.90E-05	0.195025572
ENSMUSG00000037966	Ninj1	1.89E-56	12.36496983	9.37E-18	0.206922385
ENSMUSG00000037973	Ccdc129	1.27E-20	0.075755043	7.37E-05	4.332825264
ENSMUSG00000038037	Socs1	8.13E-05	9.37668876	0.001058261	0.154410871
ENSMUSG00000038067	Csf3	5.56E-30	158.6740026	4.45E-06	0.061818728
ENSMUSG00000038756	Ttll6	1.53E-08	0.104235828	2.81E-07	6.358491421
ENSMUSG00000038791	Scgb3a2	6.31E-49	0.084987913	9.22E-15	4.705821326
ENSMUSG00000039084	Chad	9.58E-19	0.131853751	2.14E-08	4.474972991
ENSMUSG00000039196	Orm1	1.18E-129	339.8874892	1.43E-25	0.224216958
ENSMUSG00000039257	Vstm2b	3.62E-05	0.048045778	0.00219286	11.9378852
ENSMUSG00000039395	Mreg	3.68E-14	31.9637136	2.55E-13	0.17115876
ENSMUSG00000039883	Lrrc17	0.000616637	0.243811091	6.59E-05	4.211412399
ENSMUSG00000040026	Saa3	0	2579.781229	3.92E-22	0.172660624
ENSMUSG00000040752	Myh6	0.000241843	0.10771726	4.75E-05	11.22829958
ENSMUSG00000040838	Gm11639	1.00E-05	93.57545051	8.14E-06	0.048298537
ENSMUSG00000041324	Inhba	2.04E-157	121.9329236	1.17E-23	0.20114505
ENSMUSG00000041423	Paqr6	2.61E-05	0.226259601	8.35E-07	4.838055437
ENSMUSG00000041624	Gucy1a2	1.84E-06	0.231400058	6.01E-09	4.392926062
ENSMUSG00000041644	Slc5a12	1.29E-15	0.067321523	0.000887734	5.113274032
ENSMUSG00000041827	Oasl1	1.49E-05	16.86862713	0.008794306	0.160914479
ENSMUSG00000041828	Abca8a	7.14E-07	0.09744596	1.22E-08	5.502449697
ENSMUSG00000042109	Csdc2	6.37E-06	0.166999964	6.15E-06	5.638014188
ENSMUSG00000042254	Cilp	5.22E-05	0.18413199	6.00E-05	8.175059727
ENSMUSG00000042265	Trem1	1.43E-34	30.16019343	1.22E-09	0.193619862
ENSMUSG00000042349	Ikbke	1.72E-60	14.93557055	8.49E-31	0.215876167
ENSMUSG00000042529	Kcnj12	6.86E-05	0.197216587	0.000171362	4.155822795
ENSMUSG00000042793	Lgr6	2.70E-07	0.148347672	8.29E-07	5.808785189
ENSMUSG00000043333	Rhbdl2	0.000204594	7.457430182	0.001071886	0.219876023
ENSMUSG00000043496	Tril	2.05E-22	0.123317141	1.52E-18	4.56183954
ENSMUSG00000043631	Ecm2	1.33E-12	0.14508143	8.78E-09	4.554204118
ENSMUSG00000043953	Ccrl2	7.88E-56	7.891245465	2.72E-36	0.18513749
ENSMUSG00000044103	Il1f9	2.50E-25	87.98827565	3.02E-05	0.153107071
ENSMUSG00000044156	Hepacam2	4.12E-10	0.109646117	0.000539943	4.02448736
ENSMUSG00000044352	Sowaha	0.000802532	0.18377971	0.000837182	5.199540532

List 3 continued

ENSMUSG00000044365	Cxxc4	2.29E-06	0.11928619	0.000275655	5.309090454
ENSMUSG00000044701	Il27	7.98E-08	14.75646657	4.35E-06	0.131962006
ENSMUSG00000044903	Psg22	0.00046084	49.99361793	0.004566425	0.108123474
ENSMUSG00000044951	Mylk4	2.64E-05	0.051901524	0.004549182	8.655175509
ENSMUSG00000045027	Prss22	2.14E-07	18.39268603	0.000346826	0.205009629
ENSMUSG00000045362	Tnfrsf26	2.97E-53	25.28811422	1.57E-13	0.219845486
ENSMUSG00000045381	Olfr433	0.004643729	8.296292642	0.00237161	0.116784929
ENSMUSG00000045502	Hcar2	9.33E-212	138.5769777	3.13E-22	0.161589607
ENSMUSG00000045551	Fpr1	8.31E-21	12.20254813	2.02E-11	0.16264979
ENSMUSG00000045875	Adra1a	7.29E-12	0.209171927	6.17E-17	4.055325929
ENSMUSG00000045915	Ccdc42	1.38E-05	0.163779785	0.000434986	4.845972974
ENSMUSG00000046093	Hpcal4	1.41E-13	0.024406153	0.000614651	8.465944773
ENSMUSG00000046182	Gsg1l	2.68E-08	0.048168686	1.08E-05	14.90927748
ENSMUSG00000046610	Oacyl	5.53E-09	11.94195793	2.59E-06	0.211335435
ENSMUSG00000046618	Olfml2a	4.92E-18	0.15710071	5.61E-12	4.768951546
ENSMUSG00000047021	Cfap65	3.41E-09	0.197371919	5.59E-10	4.564098226
ENSMUSG00000047443	Erfe	2.02E-07	10.60548165	0.000186342	0.185781701
ENSMUSG00000047945	Marcksl1	1.92E-69	18.10556067	4.59E-38	0.146296664
ENSMUSG00000048038	Ccdc187	5.06E-15	0.168650469	3.21E-15	4.384338451
ENSMUSG00000048621	Gm6377	7.42E-20	11.36624798	1.16E-09	0.205444998
ENSMUSG00000048752	Prss50	0.00715867	4.236749485	0.002992253	0.153580059
ENSMUSG00000048834	Vstm2a	2.26E-06	0.116550481	0.00065559	5.710001072
ENSMUSG00000049511	Htr1b	2.86E-07	0.119512462	0.001262302	4.482375327
ENSMUSG00000050089	Akap4	7.02E-09	154.155024	9.28E-09	0.059285019
ENSMUSG00000050097	Ces2b	6.03E-06	0.130295888	0.000427592	5.094247704
ENSMUSG00000050359	Sprr1a	8.12E-08	10.12654822	0.000688194	0.214671204
ENSMUSG00000050395	Tnfsf15	1.52E-23	10.28317936	8.30E-16	0.180601007
ENSMUSG00000050578	Mmp13	3.37E-25	20.29990493	3.51E-10	0.228894864
ENSMUSG00000051439	Cd14	9.47E-48	32.52969399	1.65E-05	0.143146627
ENSMUSG00000051747	Ttn	2.17E-06	0.189799951	1.42E-10	5.176206679
ENSMUSG00000051748	Wfdc21	8.41E-27	16.18054827	0.000290852	0.195746578
ENSMUSG00000051906	Cd209f	1.36E-06	0.076787615	0.000113136	10.05158203
ENSMUSG00000052270	Fpr2	9.85E-46	22.71223892	1.55E-20	0.160780849
ENSMUSG00000052974	Cyp2f2	3.72E-37	0.065921921	5.69E-15	6.554004493
ENSMUSG00000053161	Daw1	0.000682899	0.182708394	0.005152125	4.344707612
ENSMUSG00000053318	Slamf8	7.73E-08	21.19083721	0.000981936	0.154935977
ENSMUSG00000053338	Tarm1	1.68E-58	107.2002244	3.85E-22	0.12474645
ENSMUSG00000053475	Tnfaip6	1.17E-05	4.947243573	1.85E-06	0.181123703
ENSMUSG00000053647	Gper1	8.88E-05	0.201738905	0.000177658	4.596918178
ENSMUSG00000054146	Krt15	0.000370582	0.033706211	0.000715217	24.79606911
ENSMUSG00000054203	Ifi205	4.80E-06	18.37378853	9.15E-05	0.106056353
ENSMUSG00000054422	Fabp1	3.30E-20	0.023984029	0.000710842	8.697051196
ENSMUSG00000054619	Mettl7a1	5.63E-37	0.102317165	3.39E-12	4.267468213
ENSMUSG00000054855	Rnd1	2.83E-12	4.206844444	1.95E-21	0.173703314

List 3 continued

ENSMUSG00000054905	Stfa3	5.49E-18	322.9239368	0.000292118	0.072935242
ENSMUSG00000055170	Ifng	5.81E-08	26.07460455	0.003114146	0.205154598
ENSMUSG00000055368	Slc6a2	1.40E-11	0.225910832	4.07E-13	4.897330345
ENSMUSG00000056054	S100a8	1.33E-08	22.40912127	8.75E-05	0.132725377
ENSMUSG00000056071	S100a9	1.23E-07	15.03851638	0.002521649	0.202527474
ENSMUSG00000056529	Ptafr	8.82E-150	50.60019052	1.33E-28	0.191338705
ENSMUSG00000057182	Scn3a	6.83E-19	0.13966351	2.26E-19	6.740149577
ENSMUSG00000057465	Saa2	1.50E-14	390.6209695	3.45E-06	0.159881304
ENSMUSG00000057606	Colq	1.17E-46	0.02647835	1.25E-17	11.79056778
ENSMUSG00000057933	Gsta2	2.19E-17	0.048290108	0.001715172	5.198246595
ENSMUSG00000058159	T2	0.002795458	0.099436358	0.005473137	8.250821183
ENSMUSG00000058427	Cxcl2	5.98E-33	3289.780123	0.0001281	0.083349823
ENSMUSG00000058755	Osm	1.93E-43	50.58503118	3.61E-11	0.146757167
ENSMUSG00000058914	C1qtnf3	9.86E-07	0.040231638	0.005661761	11.04337
ENSMUSG00000058921	Slc10a5	2.46E-11	0.081824546	0.00067873	4.585211859
ENSMUSG00000059854	Hydin	4.14E-10	0.182596109	2.40E-09	4.633299772
ENSMUSG00000060183	Cxcl11	1.41E-05	1333.409081	0.00131531	0.045257974
ENSMUSG00000061397	Krt79	2.52E-09	0.233844395	9.55E-13	4.889820187
ENSMUSG00000061462	Obscn	9.76E-06	0.162171164	3.47E-05	5.322267972
ENSMUSG00000061576	Dpp6	2.52E-05	0.064993648	0.003895059	7.661661738
ENSMUSG00000061718	Ppp1r1b	1.44E-14	0.162034749	6.17E-13	4.256544195
ENSMUSG00000061780	Cfd	1.14E-08	0.054788887	0.002698981	7.374977259
ENSMUSG00000061959	Ces1e	4.02E-21	0.080776986	1.93E-06	5.052550084
ENSMUSG00000062329	Cytl1	3.15E-37	0.052090868	4.43E-12	7.084536641
ENSMUSG00000062345	Serpinb2	6.54E-20	25.83424101	6.03E-12	0.107729598
ENSMUSG00000062480	Acat3	2.66E-09	12.91404594	0.000114596	0.229970513
ENSMUSG00000063130	Calml3	1.71E-12	0.052703411	0.001901396	5.037381352
ENSMUSG00000063234	Gpr84	8.96E-21	1317.76256	2.46E-21	0.098857983
ENSMUSG00000063626	Unc5d	0.000166992	0.052800718	0.00344372	11.70289282
ENSMUSG00000065037	Rn7sk	8.16E-07	11.71086304	0.000109069	0.101064076
ENSMUSG00000068263	Efcc1	4.13E-13	0.098555974	1.76E-05	5.168420199
ENSMUSG00000068614	Actc1	0.0001131	0.094453962	2.36E-08	6.213508853
ENSMUSG00000069792	Wfdc17	4.05E-38	26.39693598	6.71E-14	0.177469251
ENSMUSG00000069873	4930438A08Rik	0.000182692	23.50902031	0.000132369	0.060401117
ENSMUSG00000070427	Il18bp	2.37E-20	12.37359639	2.98E-07	0.225274285
ENSMUSG00000070524	Fcrlb	2.99E-37	81.89828012	5.21E-10	0.207435298
ENSMUSG00000072473	1700024G13Rik	2.72E-16	0.125294526	1.70E-07	4.581904071
ENSMUSG00000072620	Slfn2	1.19E-49	9.515633288	1.51E-26	0.242232475
ENSMUSG00000072844	G530011O06Rik	5.59E-05	18.33122749	0.004129984	0.134481634
ENSMUSG00000073274	Gm14636	8.62E-05	19.58160788	0.004488388	0.248577927
ENSMUSG00000073489	Ifi204	1.07E-08	22.83063086	0.001338531	0.153930897
ENSMUSG00000074115	Saa1	1.17E-41	549.6981917	0.003050162	0.206878911
ENSMUSG00000074505	Fat3	1.45E-07	0.209933866	2.44E-05	4.009153839
ENSMUSG00000074595	Wfdc6a	0.000208071	0.152496862	0.00129491	5.248116795

List 3 continued

ENSMUSG00000074981	Dcdc5	2.09E-05	0.08519531	0.000348263	7.591777267
ENSMUSG00000078137	Ankrd63	6.35E-24	0.143948768	1.30E-17	4.752787723
ENSMUSG00000078252	Krtap17-1	2.98E-09	0.003470597	0.003698087	41.18885152
ENSMUSG00000079101	Esd-ps	8.65E-05	13.4391246	0.006546568	0.229466222
ENSMUSG00000079597	Gm5483	4.22E-08	59.89311278	0.004320481	0.159324331
ENSMUSG00000079700	Fpr3	4.67E-09	276.7631472	1.03E-08	0.050711158
ENSMUSG00000081650	Gm16181	2.56E-08	12.2105179	3.59E-08	0.135000288
ENSMUSG00000081664	Gm15544	0.00085286	42.97431612	0.004295616	0.125778817
ENSMUSG00000082976	Gm15056	1.55E-07	189.4794036	6.30E-06	0.06420482
ENSMUSG00000084902	Gm281	2.32E-12	0.133838281	6.30E-05	4.411921832
ENSMUSG00000084941	Gm11944	4.56E-05	0.135166656	0.000721415	5.185919659
ENSMUSG00000084989	Crocc2	1.12E-06	0.175726833	7.17E-08	8.648852964
ENSMUSG00000085295	4930430E12Rik	1.23E-38	548.5327233	4.81E-23	0.171021368
ENSMUSG00000085407	1700095J03Rik	0.001204862	0.110848706	0.00178392	7.982074758
ENSMUSG00000085498	Gm14023	2.70E-08	8.602860772	6.37E-08	0.153589273
ENSMUSG00000086755	Gm11216	0.00013093	82.0423011	0.00462387	0.08536235
ENSMUSG00000086843	E030013I19Rik	2.45E-11	0.104169103	6.72E-07	6.574963686
ENSMUSG00000087477	Gm13822	5.62E-08	10.91006539	3.22E-07	0.150808062
ENSMUSG00000089874	9230117E06Rik	0.000127413	0.140465861	0.000334833	5.894619773
ENSMUSG00000090230	Gm16315	7.90E-05	19.60547242	9.81E-05	0.074344961
ENSMUSG00000090257	Gm4524	7.48E-06	0.064748871	0.003876678	7.563568983
ENSMUSG00000092418	Gm20406	1.06E-06	38.77691142	0.002628028	0.16385993
ENSMUSG00000093973	Mrgpra2a	2.57E-09	316.9026597	2.47E-05	0.117469056
ENSMUSG00000094733	Gm5416	6.61E-08	34.33204922	0.001370557	0.149498758
ENSMUSG00000095620	2010005H15Rik	5.07E-08	32.62064874	0.00010004	0.077265274
ENSMUSG00000096719	Mrgpra2b	3.64E-21	71.0739483	3.55E-07	0.115818536
ENSMUSG00000097139	Gm26626	5.26E-09	63.84590307	0.000225763	0.229560203
ENSMUSG00000097418	Mir155hg	6.60E-09	13.35425038	9.05E-06	0.189199065
ENSMUSG00000097453	Gm26894	0.001238872	0.201477614	0.006387096	4.076955691
ENSMUSG00000097504	4930516B21Rik	9.20E-09	0.132944482	8.05E-05	4.245778144
ENSMUSG00000098008	A930001A20Rik	0.000194542	0.029722809	0.000901738	25.77830895
ENSMUSG00000102269	Gm7357	0.002619754	0.075018065	0.000215932	15.32338183
ENSMUSG00000102697	Pcdhac2	3.00E-20	0.126641353	1.09E-08	4.380746328
ENSMUSG00000103308	Gm37800	4.40E-08	197.6365217	0.002001732	0.240363453
ENSMUSG00000103588	Gm18445	7.06E-05	90.8913337	6.21E-05	0.010661965
ENSMUSG00000104728	Gm42462	1.51E-29	76.54867461	1.16E-09	0.229445849
ENSMUSG00000104818	Gm43661	2.17E-05	37.50054616	0.005996952	0.182007835
ENSMUSG00000105112	Gm42778	0.000390374	0.217649401	2.02E-05	4.909549148
ENSMUSG00000105357	Gm42647	1.71E-06	81.14386275	8.39E-06	0.083694861
ENSMUSG00000105504	Gbp5	4.62E-06	18.73126225	0.003314677	0.125942953
ENSMUSG00000106609	Gm43181	2.04E-56	101.8270521	3.99E-17	0.168237453
ENSMUSG00000107191	Gm43579	0.004392962	0.023834437	0.009086609	34.60228674
ENSMUSG00000107655	Gm44220	6.10E-18	0.086944473	7.50E-11	5.245017552
ENSMUSG00000108010	Gm38708	0.000232663	0.190006271	8.86E-06	5.30647441

List 3 continued

ENSMUSG00000108161	Gm32914	0.000515901	22.61028395	0.003140755	0.129779221
ENSMUSG00000108210	Gm35808	7.62E-06	95.08709297	0.001124329	0.209049191
ENSMUSG00000108255	Gm16499	0.001017089	0.024471037	0.004047884	28.04658308
ENSMUSG00000108291	Gm44292	7.90E-05	63.9359637	0.004153633	0.224436734
ENSMUSG00000108695	Gm2511	4.20E-08	248.9724355	0.002544051	0.231985273
ENSMUSG00000108950	9130015G15Rik	1.76E-08	0.217414832	3.42E-17	5.612155047
ENSMUSG00000109251	E230032D23Rik	5.70E-13	19.1549092	2.21E-06	0.178924674
ENSMUSG00000109674	Gm45470	0.000221765	0.104704547	0.003513881	6.600834956
ENSMUSG00000110397	Gm45540	2.87E-06	0.112018733	3.41E-08	8.452724111
ENSMUSG00000112023	Lilr4b	6.99E-60	42.84896477	3.69E-17	0.243339792
ENSMUSG00000112129	Pbld1	0.000732331	0.093369879	0.001069303	7.002536749
ENSMUSG00000112146	Gm46210	2.68E-05	14.21939805	3.06E-05	0.091378979
ENSMUSG00000112762	4930459C07Rik	1.54E-05	81.66365131	0.000114755	0.1323854
ENSMUSG00000113701	B230303A05Rik	1.12E-16	255.7173117	8.46E-15	0.118919241
ENSMUSG00000113960	4933412O06Rik	2.43E-08	213.7681453	2.88E-07	0.059065617
ENSMUSG00000114161	Gm48662	2.94E-09	0.105566749	0.00048608	4.747302045
ENSMUSG00000114245	Gm38655	2.26E-09	0.128446341	1.52E-05	5.19517975
ENSMUSG00000115919	Gm31583	0.00239599	32.66660997	0.004281737	0.184554852
ENSMUSG00000115946	Mirt2	6.79E-07	24.81581919	0.006047899	0.19581
ENSMUSG00000116657	AC127341.3	7.65E-07	13.58494023	0.001953441	0.241970495
ENSMUSG00000117318	AC241601.2	0.000225845	16.10299974	0.004219055	0.1831127

List 4. GSEA, inflammatory response related genes affected by Diabetes

gene_id	gene_name	padj (diabetic vs nondiabetic)	Fold-Change (diabetic vs nondiabetic)	Rank in gene list	Rank metric score	Running ES	Leading edge subset
ENSMUSG00000039196	Orm1	1.18E-129	339.8874892	1	4.971	0.0302	Yes
ENSMUSG00000040026	Saa3	0	2579.781229	4	4.889	0.0566	Yes
ENSMUSG00000001131	Timp1	7.10E-191	81.05246061	5	4.864	0.0892	Yes
ENSMUSG00000056529	Ptafr	8.82E-150	50.60019052	6	4.806	0.1214	Yes
ENSMUSG00000057465	Saa2	1.50E-14	390.6209695	7	4.669	0.1528	Yes
ENSMUSG00000025383	Il23a	1.19E-16	50.78814098	9	4.508	0.1798	Yes
ENSMUSG00000021322	Aoah	6.94E-48	26.02086521	13	4.338	0.1993	Yes
ENSMUSG00000029082	Bst1	1.08E-63	10.95796274	19	4.165	0.2114	Yes
ENSMUSG00000035385	Ccl2	9.08E-109	202.4628751	22	4.045	0.2321	Yes
ENSMUSG00000028599	Tnfrsf1b	2.43E-80	16.38198225	26	3.959	0.2491	Yes
ENSMUSG00000023913	Pla2g7	1.07E-68	29.46701967	27	3.958	0.2757	Yes
ENSMUSG00000043953	Ccrl2	7.88E-56	7.891245465	31	3.877	0.2921	Yes
ENSMUSG00000026981	Il1rn	6.18E-180	100.7363338	33	3.735	0.314	Yes
ENSMUSG00000022534	Mefv	3.64E-37	33.73451435	35	3.723	0.3358	Yes
ENSMUSG00000026580	Selp	2.28E-35	11.34401679	38	3.581	0.3534	Yes
ENSMUSG00000004814	Ccl24	4.61E-09	7.520270475	42	3.417	0.3668	Yes
ENSMUSG00000019850	Tnfaip3	1.05E-63	15.09667011	44	3.362	0.3861	Yes
ENSMUSG00000052270	Fpr2	9.85E-46	22.71223892	45	3.354	0.4086	Yes
ENSMUSG00000024401	Tnf	7.43E-128	368.3201252	53	2.991	0.4064	Yes
ENSMUSG00000074115	Saa1	1.17E-41	549.6981917	56	2.92	0.4196	Yes
ENSMUSG00000035352	Ccl12	4.38E-30	22.2673239	57	2.899	0.4391	Yes
ENSMUSG00000032691	Nlrp3	4.94E-63	30.45240139	58	2.866	0.4583	Yes
ENSMUSG00000036931	Nfkbid	1.87E-41	14.39227709	64	2.688	0.4604	Yes
ENSMUSG00000026177	Slc11a1	3.33E-82	36.07569496	65	2.688	0.4784	Yes
ENSMUSG00000053338	Tarm1	1.68E-58	107.2002244	68	2.629	0.4897	Yes
ENSMUSG00000079700	Fpr3	4.67E-09	276.7631472	82	2.377	0.4642	Yes
ENSMUSG00000029380	Cxcl1	8.74E-62	118.7823175	86	2.336	0.4703	Yes
ENSMUSG00000027399	Il1a	1.06E-100	95.66406742	87	2.32	0.4859	Yes
ENSMUSG00000020826	Nos2	6.25E-68	61.91897814	88	2.316	0.5014	Yes
ENSMUSG00000027398	Il1b	7.90E-59	46.71511426	94	2.173	0.5001	Yes
ENSMUSG00000044103	Il1f9	2.50E-25	87.98827565	102	2.035	0.4914	Yes
ENSMUSG00000018930	Ccl4	5.00E-96	230.4394182	106	1.922	0.4948	Yes
ENSMUSG00000000982	Ccl3	4.29E-31	265.3431412	109	1.845	0.5008	Yes
ENSMUSG00000029379	Cxcl3	1.08E-24	1088.523538	112	1.786	0.5064	Yes
ENSMUSG00000058755	Osm	1.93E-43	50.58503118	113	1.785	0.5184	Yes
ENSMUSG00000029371	Cxcl5	0.00027586	25.58258012	116	1.753	0.5237	Yes
ENSMUSG00000026073	Il1r2	1.27E-69	95.9813252	118	1.733	0.5322	Yes
ENSMUSG00000037872	Ackr1	1.79E-09	21.34661068	130	1.643	0.5082	Yes
ENSMUSG00000042265	Trem1	1.43E-34	30.16019343	131	1.631	0.5191	Yes
ENSMUSG00000051439	Cd14	9.47E-48	32.52969399	139	1.539	0.5071	Yes
ENSMUSG00000035373	Ccl7	5.09E-14	84.49006544	142	1.522	0.511	Yes
ENSMUSG00000044701	Il27	7.98E-08	14.75646657	144	1.514	0.5179	Yes
ENSMUSG00000045551	Fpr1	8.31E-21	12.20254813	146	1.498	0.5248	Yes
ENSMUSG00000032487	Ptgs2	5.53E-12	6.263341608	147	1.492	0.5348	Yes
ENSMUSG00000025746	Il6	4.75E-13	128.5313558	155	1.382	0.5218	No
ENSMUSG00000056054	S100a8	1.33E-08	22.40912127	159	1.333	0.5212	No

List 4 continued

ENSMUSG00000056071	S100a9	1.23E-07	15.03851638	161	1.32	0.5268	No
ENSMUSG00000058427	Cxcl2	5.98E-33	3289.780123	165	1.281	0.5259	No
ENSMUSG00000053475	Tnfaip6	1.17E-05	4.947243573	171	1.214	0.5181	No
ENSMUSG00000016529	Il10	3.91E-08	129.1821376	176	1.156	0.5131	No
ENSMUSG00000026984	Il1f6	2.17E-05	135.1112892	183	1.065	0.5012	No
ENSMUSG00000105504	Gbp5	4.62E-06	18.73126225	199	0.919	0.4595	No
ENSMUSG00000034855	Cxcl10	5.60E-11	178.795138	202	0.852	0.4589	No
ENSMUSG00000029417	Cxcl9	5.53E-08	99.62982301	205	0.796	0.4579	No
ENSMUSG00000060183	Cxcl11	1.41E-05	1333.409081	207	0.715	0.4595	No
ENSMUSG00000058914	C1qtnf3	9.86E-07	0.040231638	289	-2.207	0.2163	No
ENSMUSG00000043496	Tril	2.05E-22	0.123317141	310	-2.481	0.1693	No
ENSMUSG00000053647	Gper1	8.88E-05	0.201738905	343	-3.254	0.0892	No

44 genes were leading edge subset genes (blue)

List 5. GSEA, inflammatory response related genes affected by DMOG

gene_id	gene_name	padj (DMOG vs vehicle)	Fold-Change (DMOG vs vehicle)	Rank in gene list	Rank metric score	Running ES	Leading edge subset
ENSMUSG00000043496	Tril	1.52E-18	4.56183954	16	3.197	-0.0231	No
ENSMUSG00000053647	Gper1	0.000177658	4.596918178	101	2.191	-0.2716	No
ENSMUSG00000058914	C1qtnf3	0.005661761	11.04337	150	1.274	-0.4133	No
ENSMUSG00000060183	Cxcl11	0.00131531	0.045257974	164	-0.701	-0.4486	No
ENSMUSG00000029417	Cxcl9	0.00626627	0.111104922	165	-0.706	-0.4425	No
ENSMUSG00000034855	Cxcl10	2.95E-05	0.050853551	171	-0.809	-0.4514	No
ENSMUSG00000105504	Gbp5	0.003314677	0.125942953	178	-0.835	-0.4632	No
ENSMUSG00000016529	Il10	9.73E-06	0.105488266	195	-0.993	-0.5055	No
ENSMUSG00000056071	S100a9	0.002521649	0.202527474	199	-1.054	-0.5059	No
ENSMUSG00000058427	Cxcl2	0.0001281	0.083349823	204	-1.103	-0.509	No
ENSMUSG00000056054	S100a8	8.75E-05	0.132725377	211	-1.17	-0.518	Yes
ENSMUSG00000026984	Il1f6	0.000363903	0.013949402	215	-1.211	-0.517	Yes
ENSMUSG00000025746	Il6	1.08E-05	0.082454048	217	-1.227	-0.5095	Yes
ENSMUSG00000037872	Ackr1	9.90E-05	0.195025572	220	-1.249	-0.505	Yes
ENSMUSG00000042265	Trem1	1.22E-09	0.193619862	224	-1.279	-0.5034	Yes
ENSMUSG00000051439	Cd14	1.65E-05	0.143146627	225	-1.282	-0.4922	Yes
ENSMUSG00000053475	Tnfaip6	1.85E-06	0.181123703	226	-1.296	-0.4809	Yes
ENSMUSG00000029379	Cxcl3	9.13E-09	0.209041893	228	-1.318	-0.4727	Yes
ENSMUSG00000045551	Fpr1	2.02E-11	0.16264979	231	-1.334	-0.4674	Yes
ENSMUSG00000026073	Il1r2	2.96E-11	0.190130736	233	-1.337	-0.459	Yes
ENSMUSG00000035373	Ccl7	1.19E-14	0.1001819	236	-1.344	-0.4536	Yes
ENSMUSG00000044701	Il27	4.35E-06	0.131962006	246	-1.394	-0.4702	Yes
ENSMUSG00000032487	Ptgs2	8.06E-10	0.22992373	249	-1.43	-0.4641	Yes
ENSMUSG00000058755	Osm	3.61E-11	0.146757167	252	-1.452	-0.4578	Yes
ENSMUSG00000000982	Ccl3	6.83E-07	0.109129139	259	-1.52	-0.4637	Yes
ENSMUSG00000044103	Il1f9	3.02E-05	0.153107071	260	-1.523	-0.4504	Yes
ENSMUSG00000074115	Saa1	0.003050162	0.206878911	268	-1.624	-0.4586	Yes
ENSMUSG00000029371	Cxcl5	2.00E-16	0.135063534	269	-1.649	-0.4442	Yes
ENSMUSG00000018930	Ccl4	4.73E-26	0.059414395	276	-1.741	-0.4482	Yes
ENSMUSG00000035352	Ccl12	2.46E-08	0.221482926	284	-1.899	-0.4539	Yes
ENSMUSG00000027398	Il1b	1.67E-24	0.117804928	285	-1.9	-0.4374	Yes
ENSMUSG00000026177	Slc11a1	2.63E-13	0.232333606	286	-1.902	-0.4208	Yes
ENSMUSG00000036931	Nfkbid	1.30E-11	0.218189854	287	-1.906	-0.4042	Yes
ENSMUSG00000029380	Cxcl1	2.36E-15	0.095824333	288	-1.907	-0.3876	Yes
ENSMUSG00000027399	Il1a	1.39E-24	0.090170443	294	-1.986	-0.3863	Yes
ENSMUSG00000020826	Nos2	5.29E-26	0.06849956	300	-2.067	-0.3842	Yes
ENSMUSG00000032691	Nlrp3	4.61E-16	0.203978559	303	-2.094	-0.3723	Yes
ENSMUSG00000053338	Tarm1	3.85E-22	0.12474645	309	-2.182	-0.3693	Yes
ENSMUSG00000019850	Tnfaip3	2.49E-17	0.213885176	318	-2.452	-0.3734	Yes
ENSMUSG00000026981	Il1rn	2.87E-15	0.174795811	321	-2.472	-0.3583	Yes
ENSMUSG00000024401	Tnf	3.58E-27	0.081748552	323	-2.488	-0.3398	Yes
ENSMUSG00000057465	Saa2	3.45E-06	0.159881304	324	-2.495	-0.3181	Yes
ENSMUSG00000004814	Ccl24	7.33E-06	0.212934934	327	-2.528	-0.3024	Yes

List 5 continued

ENSMUSG00000052270	Fpr2	1.55E-20	0.160780849	331	-2.639	-0.289	Yes
ENSMUSG00000079700	Fpr3	1.03E-08	0.050711158	334	-2.737	-0.2716	Yes
ENSMUSG00000022534	Mefv	4.08E-22	0.215845775	336	-2.768	-0.2506	Yes
ENSMUSG00000023913	Pla2g7	2.32E-26	0.225761091	337	-2.802	-0.2262	Yes
ENSMUSG00000028599	Tnfrsf1b	1.65E-24	0.236599915	338	-2.806	-0.2018	Yes
ENSMUSG00000026580	Selp	1.18E-20	0.214382646	340	-2.883	-0.1799	Yes
ENSMUSG00000001131	Timp1	6.55E-18	0.208331927	341	-2.918	-0.1545	Yes
ENSMUSG00000025383	Il23a	4.65E-11	0.144711817	346	-3.116	-0.1401	Yes
ENSMUSG00000021322	Aoah	6.36E-37	0.230843126	350	-3.128	-0.1224	Yes
ENSMUSG00000029082	Bst1	2.24E-35	0.227576107	352	-3.149	-0.0982	Yes
ENSMUSG00000039196	Orm1	1.43E-25	0.224216958	353	-3.172	-0.0706	Yes
ENSMUSG00000040026	Saa3	3.92E-22	0.172660624	356	-3.216	-0.049	Yes
ENSMUSG00000035385	Ccl2	5.44E-35	0.109975304	358	-3.255	-0.0238	Yes
ENSMUSG00000056529	Ptafr	1.33E-28	0.191338705	362	-3.321	-0.0045	Yes
ENSMUSG00000043953	Ccrl2	2.72E-36	0.18513749	365	-3.44	0.0191	Yes

48 genes were leading edge subset genes (in color), and 4 genes were not involved in leading edge subset genes of List 4 (red).
